# Hybrids of Benzenesulfonamide Oxadiazole Derivatives with Dual CA II and COX‑2 Inhibitory Activity Demonstrating Antiglaucoma and Anti-inflammatory Action: Synthesis, In Silico Insights, and In Vitro and In Vivo Bioevaluation

**DOI:** 10.1021/acs.jmedchem.6c01117

**Published:** 2026-07-02

**Authors:** Manal Abdel Fattah Ezzat, Emad M. Seif, Husam Nassar, Alessandro Bonardi, Marta Ferraroni, Ahmed A. Attia, Yomna T. T. Khater, Rabab Ahmed Rasheed, Omnia A. M. Abd El-Ghafar, Heba Abdelrasheed Allam, Andrea Angeli, Matthias Schmidt, Claudiu T. Supuran, Hany S. Ibrahim

**Affiliations:** † Department of Pharmaceutical Chemistry, Faculty of Pharmacy, 63526 Cairo University, Cairo 11562, Egypt; ‡ Department of Pharmaceutical Chemistry, Faculty of Pharmacy, October University for Modern Sciences and Arts University (MSA), Giza 12451, Egypt; § Department of Medicinal Chemistry, Institute of Pharmacy, 9176 Martin-Luther-University of Halle–Wittenberg, Halle (Saale) 06120, Germany; ∥ Department NEUROFARBA—Pharmaceutical and Nutraceutical Section, 9300 University of Firenze, Via Ugo Schiff 6, Sesto Fiorentino I-50019, Firenze, Italy; ⊥ Department of Chemistry “Ugo Schiff”, University of Florence, Via della Lastruccia 3-13, Sesto Fiorentino I-50019, Italy; # Mansoura Ophthalmic Centre, Faculty of Medicine, Mansoura University, Mansoura 35516, Egypt; ¶ Medical Experimental Research Center, Faculty of Medicine, Mansoura University, Mansoura 35516, Egypt; ∇ Department of Medical Histology and Cell Biology, School of Medicine, Badya University, Giza 12573, Egypt; ○ Department of Pharmacology and Toxicology, Faculty of Pharmacy, 312505 Nahda University, Beni-Suef 62764, Egypt; ⧫ NEUROFARBA Department, Sezione di Scienze Farmaceutiche, University of Florence, Via Ugo Schiff 6, Sesto Fiorentino 50019, Florence, Italy; †† Department of Pharmaceutical Chemistry, Faculty of Pharmacy, Egyptian Russian University, Badr City, Cairo 11829, Egypt

## Abstract

In this study, new sulfonamide derivatives **5a–g** and **10a–e** were designed, synthesized, and biologically evaluated for their anti-inflammatory activity. In vitro COX inhibitory assays were performed, and among the synthesized compounds, **5b** and **5d** emerged as the most promising leads, combining COX-2 inhibition with remarkable selectivity (COX-2 IC_50_ = 0.13 and 0.05 μM, SI = 9.25 and 12.02, respectively) and *h*CA II inhibition (*K*_i_ = 39.1 nM and 62.6 nM, respectively) with a high selectivity index over *h*CA I (SI = 933.8 and 704.2, respectively). In vivo evaluations confirmed that compounds **5b** and **5d** (50 mg/kg) possess promising analgesic and anti-inflammatory effects, with rapid onset, sustained duration of action, and a reduced ulcerogenic liability, indicating an improved gastrointestinal safety profile. Additionally, **5b** showed significant and sustained IOP-lowering effects in antiglaucoma animal models. Computational studies and X-ray crystallography were performed as a proof of concept.

## Graphical abstract

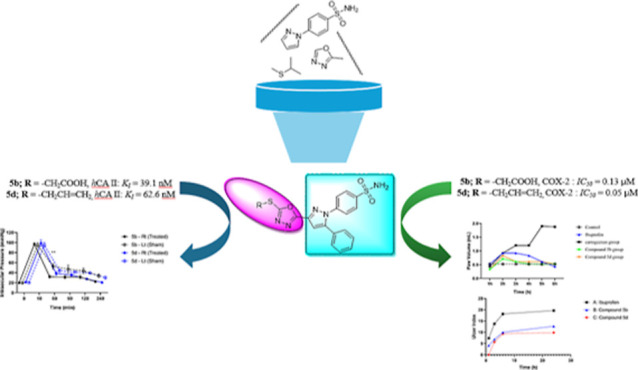

## 1 Introduction

Inflammation is the body’s normal defense mechanism for harmful assaults, including noxious stimuli, tissue injury, or infections.^1^ The most significant class of widely prescribed medications for the management of pain and inflammation are nonsteroidal anti-inflammatory drugs (NSAIDs) through blocking cyclooxygenases (COXs), the primary regulators of arachidonic acid conversion into prostaglandins. Long-term use of traditional NSAIDs is linked to gastrointestinal (GI) irritation, bleeding, and ulceration due to their high selectivity for COX-1 over COX-2. Therefore, developing anti-inflammatory medications that discriminate between therapeutic and adverse effects is a strategy for boosting COX-2 specificity and enhancing safety for long-term prophylaxis of chronic diseases.^2,3^ Attempts to explore selective COX-2 inhibitors have yielded numerous diarylheterocycles and core ring pharmacophore models. Celecoxib **I** and SC-558 **II** (Figure 1) are regarded as typical templates of a pyrazole central scaffold bearing 1,3-diphenyl moieties,^4,5^ followed subsequently by valdecoxib. However, the global withdrawal of rofecoxib (Vioxx) after evidence linking its use to an increased risk of cardiovascular events raised significant concerns regarding the overall cardiovascular safety profile of COX-2 selective inhibitors.^4^

**Figure 1:**
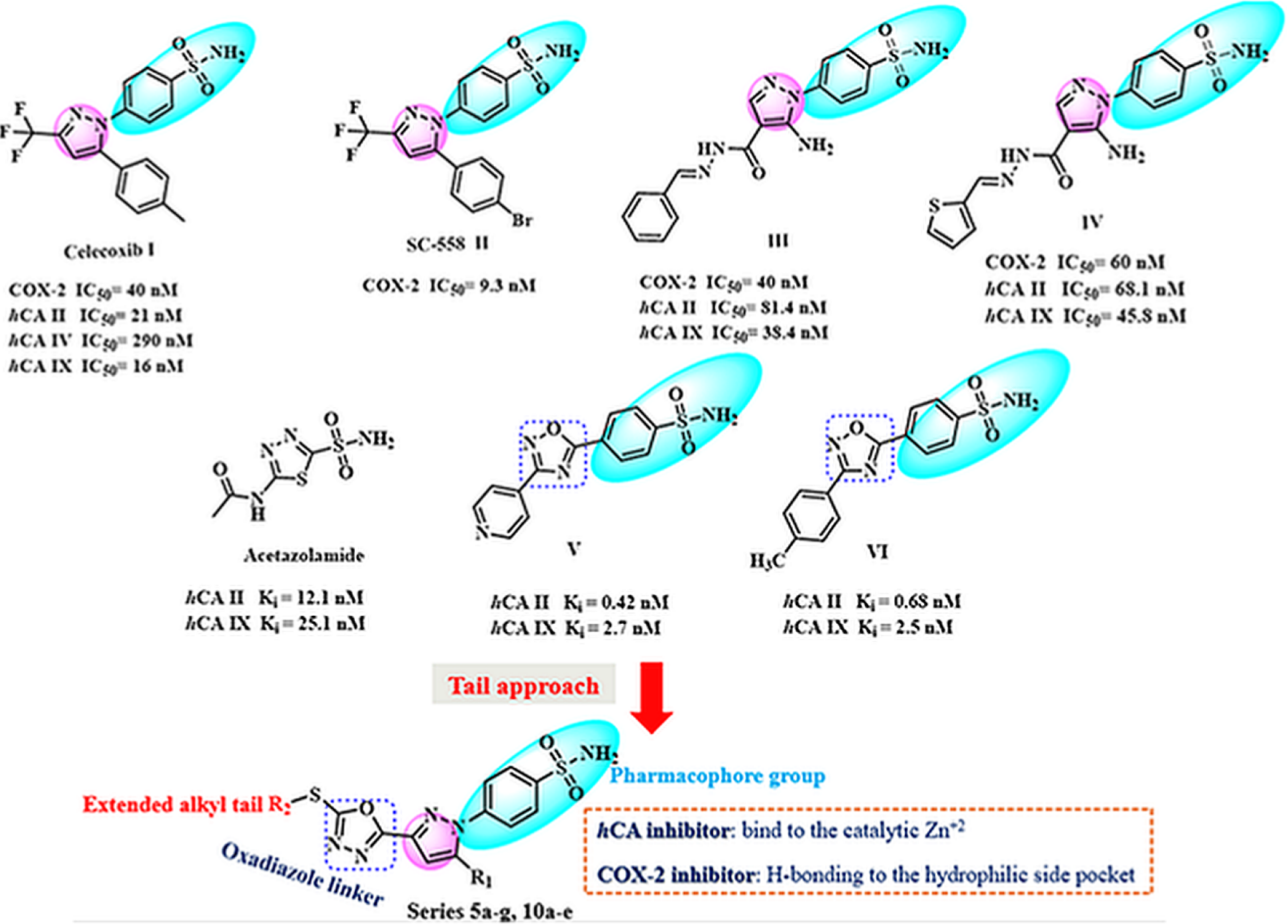
Design of pyrazole-based derivatives **5a–g** and **10a–e** targeting both *h*CA II and COX-2 isoforms.

The current understanding of inflammation extends beyond prostaglandin-mediated pathways to include additional contributing mechanisms, notably tissue acidosis, which plays a significant role in the sensitization of pain and the progression of the inflammatory responses. Therefore, targeting pH-regulating enzymes such as carbonic anhydrases (CAs) represents a complementary strategy that may enhance the anti-inflammatory efficacy when combined with COX-2 inhibition.^6^

Carbonic anhydrases (CAs) are ubiquitous metalloenzymes that exert a dual therapeutic effect: (i) as anti-inflammatory agents, they regulate pH by catalyzing the reversible hydration of carbon dioxide to yield protons and bicarbonate ions;^7,8^ (ii) as antiglaucoma agents, remarkably, the inhibition of human carbonic anhydrase (*h*CA) I, II, and IV isoforms with a minor contribution from CA XII reduces intraocular pressure (IOP) by inhibiting aqueous humor production within the ciliary body of the eye.^9^

The free sulfonamide functionality of CAs does not only act as a catalytic Zn^2+^ binding candidate for the most potent CA inhibitors (CAIs) but also as an important pharmacophoric feature for selective COX-2 inhibitor development (Figure 1).^10^ Celecoxib **I**, a well-known sulfonamide-tethered anti-inflammatory drug, effectively inhibits *h*CA II, exhibiting IC_50_ values in the submicromolar range. Compounds **III** and **IV** (Figure 1) are aminoaryl pyrazole derivatives that have been reported to exhibit dual inhibitory activity against both COX-2 and carbonic anhydrase (CA) enzymes.^10–15^ Sulfonamide-based *h*CAIs’ main drawback, however, is their lack of selectivity. Hence, Supuran’s group established the “tail approach” to overcome the selectivity hurdle and appropriately target their activity toward a particular CA isoform.^16^ The elongated molecule with its new tail (usually on a benzenesulfonamide scaffold) can precisely interact with the nonconserved amino acid residues in the middle/outer rims of the CA’s active region (Figure 2).^17,18^

**Figure 2:**
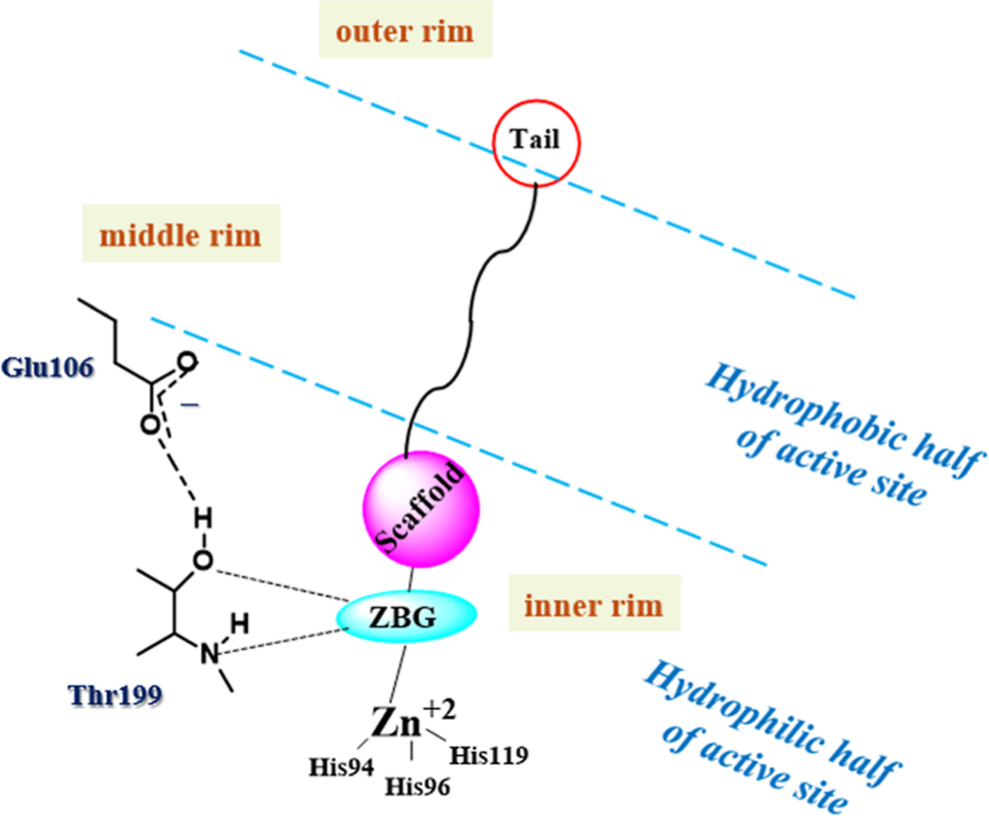
Diagrammatic illustration of the tail approach for designing selective zinc-binding CAIs by the tail approach.

Designing single-entity ligands has emerged as a robust approach to address complex pathologies such as glaucoma, where the concurrent carbonic anhydrase and cyclooxygenase pathway modulation can provide synergistic therapeutic benefits.^19,20^

Motivated by the aforementioned findings, we embarked on the design and synthesis of a series of oxadiazole-bearing (pyrazol-1-yl)­benzenesulfonamide derivatives **5a–g** and **10a–e** that maintain celecoxib’s 4-(pyrazol-1-yl)­benzenesulfonamide pharmacophoric moiety. The integrated free sulfonamide functional group is anticipated not only to bind the catalytic Zn^2+^ of *h*CA but also to induce COX-2 selectivity through the formation of a hydrogen bond with the ligand binding pocket’s hydrophilic amino acid residues. Krasavin et al. developed a series of oxadiazol-5-yl benzenesulfonamides **V** and **VI** as selective inhibitors of *h*CA **II** and **IX** isoforms at subnanomolar levels, surpassing acetazolamide^21^ (Figure 1). Consequently, the inhibitory efficacy was expected to be augmented via incorporation of various extended alkyl tails through an oxadiazole linker, affording the targeted compounds **5a–g** and **10a–e** (Figures 1 and 2).

All of the synthesized compounds were assessed for COX-1 and COX-2 inhibition, in addition to their *h*CA I, II, and XII inhibitory activities. Moreover, the analgesic, anti-inflammatory, and ulcerogenic properties and antiglaucoma potential were estimated for pyrazoles **5b** and **5d**, exhibiting favorable COX-2 potency and selectivity while maintaining acceptable carbonic anhydrase II activity.

## 2 Results and Discussion

### 2.1 Chemistry

Two series of 4-(4-(5-(mercapto/thiosubstituted)-1,3,4-oxadiazol-2-yl)-5-phenyl-1*H*-pyrazol-1-yl)­benzenesulfonamide **5a–g** and 4-(5-amino-4-(5-(thio)-1,3,4-oxadiazol-2-yl)-1*H*-pyrazol-1-yl)­benzenesulfonamide **10a–e** derivatives have been synthesized as illustrated in Schemes 1 and 2, respectively. Scheme 1 illustrates the pathway for the synthesis of derivatives **5a–g**, which started with the reflux of ethyl benzoyl acetate (**1**) with DMF–DMA (formylating/enaminating agent), affording the aminoacrylate enaminone derivative (**2**) (yield = 78%). Then, derivative (**2**) was cyclized with 4-aminosulfonylphenylhydrazine hydrochloride in refluxing absolute ethanol to give the pyrazole-4-carboxylate derivative (**3**) in 69% yield, which was employed in the next hydrazinolysis step through fusion with hydrazine hydrate to produce the hydrazide derivative (**4**) (yield = 56%). Regarding the second series, **10a–e** (Scheme 2), it started with the reflux of ethylcyanoacetate (**6**) with triethyl orthoformate in acetic anhydride, yielding 76% ethyl (ethoxyethylene)­cyanoacetate (**7**). Following this, derivative (**7**) cyclized with 4-aminosulfonylphenylhydrazine in a mixture of acetic acid/water (5:1) under reflux to give the pyrazole-4-carboxylate derivative (**8**) in 68% yield, which was subsequently subjected to hydrazinolysis to form the corresponding hydrazide derivative (**9**) (yield = 57%).^22^ Finally, the target 1,3,4-oxadiazole derivatives **5a–g** (Scheme 1) and **10a–e** (Scheme 2) were afforded by the reaction of the acid hydrazide derivatives **4** and **9**, respectively, with carbon disulfide in DMF at 70 °C for 4 h via a multistep process involving the initial formation of a dithiocarbazate intermediate, followed by intramolecular cyclization and ring closure. Following cyclization, the thiol intermediate is deprotonated by triethylamine (TEA) to form a reactive thiolate, which then undergoes S-alkylation by stirring overnight with alkyl halides to yield the corresponding thioether products.^12^

**Scheme 1:**
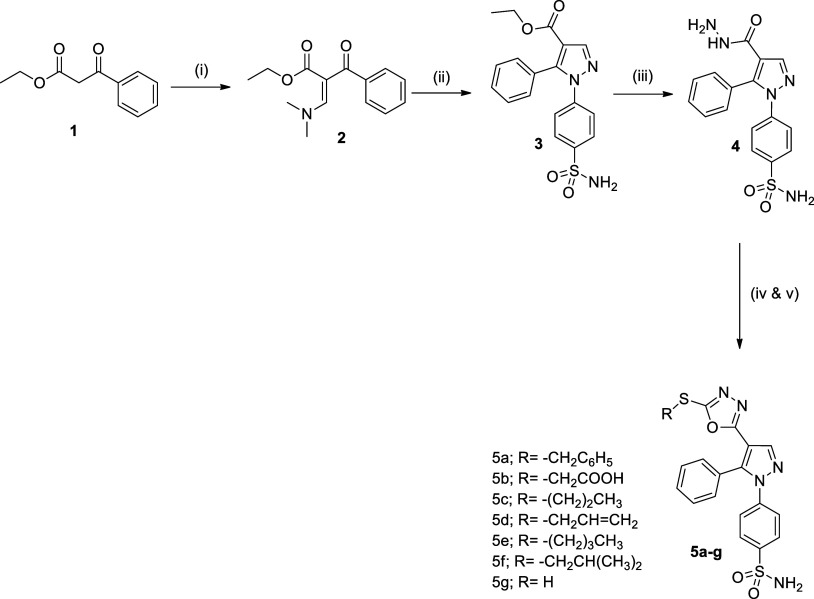
Synthesis of Compounds **5a–g** ^a^

a Reagents and conditions: (i) DMF–DMA, reflux 2 h; (ii) *p*-SO_2_NH_2_C_6_H_4_NHNH_2_·HC1/abs. Ethanol/reflux 4 h; (iii) N_2_H_4_·H_2_O/reflux 3 h; (iv) CS_2_/DMF/stir overnight/rt; (v) RBr/TEA/stir/rt.

**Scheme 2:**
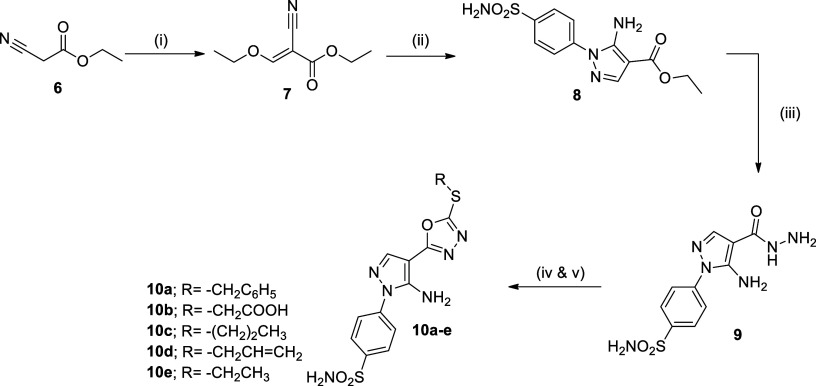
Synthesis of Compounds **10a–e** ^a^

a Reagents and conditions; (i) triethylorthoformate/acetic anhydride/reflux 10 h; (ii) *p*-SO_2_NH_2_C_6_H_4_NHNH_2_·HCl/acetic acid/H_2_O/reflux 4 h; (iii) N_2_H_4_·H_2_O/reflux 4 h; (iv) CS_2_/DMF/stir overnight/rt; (v) RBr/TEA/stir/rt.

^1^H NMR spectra confirmed the structures of the target derivatives **5a–g** and **10a–e**. The benzyl derivatives **5a** and **10a** demonstrated additional singlet signals at δ 4.36 and 4.5 ppm, respectively, of the benzylic CH_2_ protons. Also, the spectrum of derivative **5g** showed a singlet D_2_O exchangeable signal of the mercapto group at δ 14.51 ppm. Moreover, after stirring overnight with the appropriate alkyl halide, the corresponding S-alkylated derivatives **5c–f** and **10c–e** demonstrated the aliphatic signals with a range of δ 0.85–3.28 ppm assigned to these groups. Also, the allylic protons of **5d** and **10d** were confirmed as they showed signals around δ 5.07–5.99 ppm. Furthermore, for acetic acid derivatives **5b** and **10b**, the spectra showed singlet signals of carboxylic CH_2_ at δ 3.79 and 4.18 ppm, respectively. Regarding the ^13^C NMR spectra, they revealed the *S*-alkyl substitution with aliphatic signals between δ 13.18 and 40.58 ppm for derivatives **5c**, **5e**, **5f**, **10c**, and **10e**. Additionally, for the *S*-benzyl substituted derivatives **5a** and **10a**, the spectra showed a signal for the benzylic carbon at δ 36.29 and 36.52 ppm, respectively. Referring to the carboxylic acid derivatives **5b** and **10b**, a signal at 168.93 and 169.50, respectively, is assigned to the carboxylic carbonyl carbon. Also, the *S*-allyl substituted derivatives **5d** and **10d** exhibited three signals in the range of δ 35.06–133.31 ppm attributed to the three allylic carbons.

### 2.2 Biological Evaluation

#### 2.2.1 COX-1 and COX-2 Inhibitory Assay

All the new target sulfonamide derivatives **5a–g** and **10a–e** were in vitro assessed for both their COX-1 and COX-2 inhibitory activities using celecoxib as a reference drug, and the results were displayed as IC_50_ values. In addition, the compounds’ safety profile was investigated and the selectivity indices (SIs) toward COX-2 were determined. As depicted in Table 1, six compounds **5b**, **5c**, **5d**, **5g**, **10c**, and **10e** (IC_50_ ranges between 0.05 and 0.17 μM) had comparable COX-2 inhibitory activity compared to the reference celecoxib (IC_50_ = 0.05 μM), while derivatives **5a** and **10d** revealed moderate potency with IC_50_ values of 0.46 and 0.42 μM, respectively.

**Table 1:**
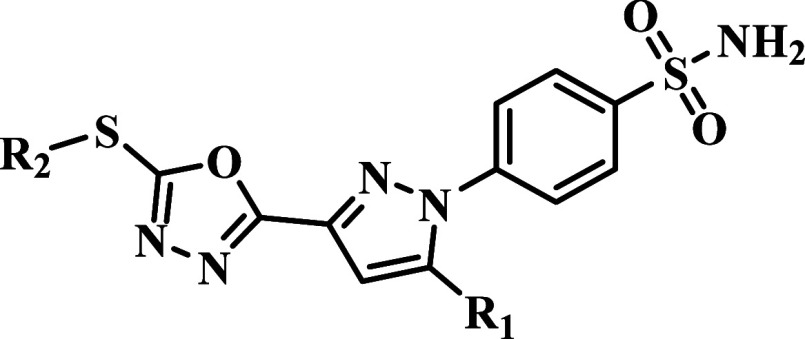
IC_50_ Values of the New Sulfonamide Derivatives **5a–g** and **10a–e** as COX Inhibitors in Comparison with Celecoxib as a Reference Drug

Compound	R_1_	R_2_	COX-1 inhibition [IC_50_ μM ± SD]^a^	COX-2 inhibition [IC_50_ μM ± SD]^a^	SI (COX-1/COX-2)
**5a**	Ph	–CH_2_–C_6_H_5_	0.45 ± 0.20	0.46 ± 0.18	0.97
**5b**	Ph	–CH_2_–COOH	1.19 ± 0.50	0.13 ± 0.05	**9.25**
**5c**	Ph	–CH_2_CH_2_CH_3_	1.19 ± 0.48	0.17 ± 0.06	7.07
**5d**	Ph	–CH_2_CH=CH_2_	0.64 ± 0.26	0.05 ± 0.02	**12.02**
**5e**	Ph	CH2CH2CH2CH3	5.55 ± 1.97	0.62 ± 0.23	8.98
**5f**	Ph	–CH_2_CH(CH_3_)_2_	2.35 ± 0.86	1.21 ± 0.46	1.94
**5g**	Ph	H	1.34 ± 0.62	0.17 ± 0.07	7.95
**10a**	NH_2_	–CH_2_–C_6_H_5_	6.59 ± 2.21	1.54 ± 0.50	4.27
**10b**	NH_2_	–CH_2_–COOH	9.51 ± 3.32	2.65 ± 0.84	3.59
**10c**	NH_2_	–CH_2_CH_2_CH_3_	0.96 ± 0.40	0.15 ± 0.06	6.35
**10d**	NH_2_	–CH_2_CH=CH_2_	2.40 ± 0.88	0.42 ± 0.17	5.71
**10e**	NH_2_	–CH_2_CH_3_	0.29 ± 0.13	0.07 ± 0.03	4.18
**Celecoxib**		-	1.51 ± 0.66	0.05 ± 0.11	29.22

a The data are presented as the average IC_50_ values (mean ± SD) of three independent replicates.

Comparing the COX-1 and COX-2 isozymes’ inhibitory effects, all new sulfonamide derivatives **5a–g** and **10a–e** revealed enhanced COX-2 inhibitory efficacy (IC_50_ ranges between 0.05 and 2.65 μM) in comparison to COX-1 (IC_50_ ranges between 0.29 and 9.51 μM) with good selectivity indices. In particular, 1,5-diphenylpyrazoles **5b**, **5c**, and **5d** demonstrated outstanding selectivity indices (SI ranges between 7.07 and 12.02) rather than their corresponding (5-amino-1*H*-pyrazol-1-yl)­benzenesulfonamide analogues **10b**, **10c**, and **10d** (SI ranges between 3.59 and 6.35) against the COX-2 isoenzyme, except for the 5-(benzylthio)-1,3,4-oxadiazol-2-yl derivative **5a**, which showed a lower selectivity index (SI = 0.97) than its corresponding analogue **10a** (SI = 4.27).

Considering the series **5a–g**, the SAR investigation revealed that COX-2 inhibitory activity was improved in the derivatives **5c** and **5d** (IC_50_ range = 0.17 and 0.05 μM, respectively) with alkylated side chain substitution or with freely ionizable centers in **5b** and **5g** with IC_50_ in the range 0.13–0.17 μM, except for butyl **5e** and isopropyl **5f** derivatives that exhibited lower IC_50_ values of 0.62 μM and 1.21 μM, respectively. Moreover, the introduction of a benzylic side chain led to moderate COX-2 inhibitory activity, as demonstrated by compound **5a** (IC_50_ = 0.46 μM). However, in series **10a–e**, the derivatives with alkylated side chain substitution in **10c–10e** demonstrated greater potency (IC_50_ range = 0.07–0.42 μM) rather than the ionizable carboxylic acid derivative **10b** (IC_50_ = 2.65 μM) or the derivative inserted with a benzylic side chain as **10a** (IC_50_ = 1.54 μM). It is worth mentioning that compounds **5b** and **5d** have emerged as the most promising compounds combining notable COX-2 inhibition with excellent selectivity indices of 9.25 and 12.02, respectively.

Moreover, COX-2 selectivity was strongly affected by both the pyrazole C_5_ substituent (R_1_) and the oxadiazole thio substituent (R_2_). The 1,5-diphenylpyrazole derivatives **5a–g** (R_1_ = Ph) were generally more selective than the corresponding 5-amino analogues **10a–e** (R_1_ = NH_2_), indicating that the bulky phenyl group better fits the larger COX-2 secondary pocket. Compounds **5b**, **5c**, and **5d** showed selectivity indices higher than those of **10b**, **10c**, and **10d**. Among R_2_ substituents, the allyl derivative **5d** showed the highest selectivity (SI = 12.02), followed by the carboxymethyl derivative **5b** (SI = 9.25), suggesting that small flexible alkyl chains and ionizable groups enhance selective COX-2 binding rather than bulky benzylic, long-chain, or branched alkyl substituents. Thus, compounds **5b** and **5d** were the most promising selective COX-2 inhibitors.

#### 2.2.2 Carbonic Anhydrase (CA) Inhibitory Assay

A carbonic anhydrase inhibition assay for all derivatives has been performed against three human carbonic anhydrase (*h*CA) isoforms: *h*CA I, *h*CA II, and *h*CA XII. The method used in the assay was the stopped-flow CO_2_ hydrase assay (Table 2).^23^

**Table 2:**
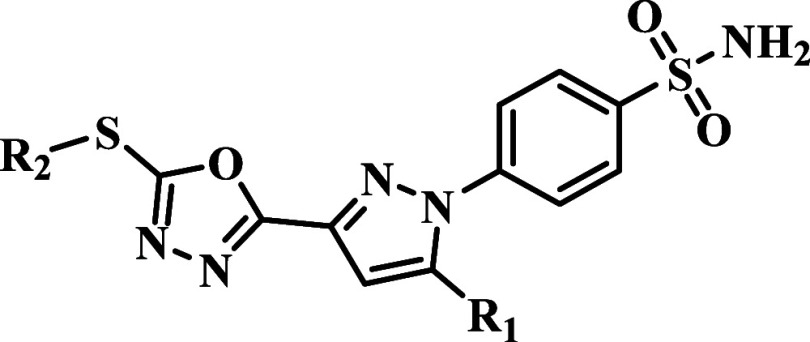
Inhibition Assay for the Synthesized **5a–g** and **10a–e** against *h*CA I, *h*CA II, and *h*CA XII Using Acetazolamide (AAZ) as a Reference Drug

			*K*_i_ (nM)^a^			Selectivity Indices (SI)	
Compound	R_1_	R_2_	*h*CA I	*h*CA II	*h*CA XII	I/II	XII/II
**5a**	Ph	–CH_2_–C_6_H_5_	42,140	43.7	60.9	964.3	1.39
**5b**	Ph	–CH_2_–COOH	36,510	39.1	34.2	933.8	0.87
**5c**	Ph	–CH_2_CH_2_CH_3_	46,630	67.8	61.1	687.8	0.90
**5d**	Ph	–CH_2_CH=CH_2_	44,080	62.6	69.7	704.2	1.11
**5e**	Ph	–CH_2_CH_2_CH_2_CH_3_	50,270	48.2	54.6	1042.9	1.13
**5f**	Ph	–CH_2_CH(CH_3_)_2_	47,390	54.1	75.3	875.9	1.39
**5g**	Ph	H	57,860	50.5	39.2	1145.7	0.78
**10a**	NH_2_	–CH_2_–C_6_H_5_	938.6	85.4	53.4	10.9	0.63
**10b**	NH_2_	–CH_2_–COOH	425.8	61.6	52.9	6.9	0.86
**10c**	NH_2_	–CH_2_CH_2_CH_3_	1387	73.9	74.3	18.8	1.01
**10d**	NH_2_	–CH_2_CH=CH_2_	2546	102.3	66.4	24.9	0.65
**10e**	NH_2_	–CH_2_CH_3_	1798	93.1	61.8	19.3	0.66
**AAZ**	-	-	250.0	12.0	5.7	20.8	0.48

a The mean of 3 different assays, by a stopped-flow technique (errors were in the range of ±5–10% of the reported values).

Compared to the reference compound AAZ, all derivatives exhibited a less favorable inhibitory profile against the off-target isoform *h*CA I as well as against the target isoforms *h*CA II and *h*CA XII while still retaining potency in the low double-digit nanomolar range toward the latter two isoforms. Nevertheless, this modulation resulted in compounds **5a–g** with a markedly improved selectivity relative to AAZ (SI I/II = 687.8–1145.7), together with a slightly enhanced selectivity profile favoring *h*CA XII over *h*CA II (SI XII/II = 0.63–1.39) for all compounds. Such selectivity is particularly important for minimizing the off-target side effects commonly associated with CA inhibitors.

Based on the results of CA inhibition assays, it is observed that replacing the small 5-amino group of **10a–e** derivatives (*h*CA I *K*_i_ range: 425.8 nM (**10b**) to 2546 nM (**10d**)) with a bulky phenyl moiety in **5a**–**g** series (*h*CA I *K*_i_ values above 36,500 nM) drastically decreases the affinity toward *h*CA I, which improves the derivatives’ selectivity.

Interestingly, the acetic acid derivative **10b** with the 5-amino group showed superior potency against *h*CA I (*K*_i_ = 425.8 nM), suggesting a favorable interaction binding mode with *h*CA I and helping design highly selective inhibitors. Moreover, **10b** is unique among the derivatives in its selectivity toward *h*CA XII when compared to *h*CA II.

Conspicuously, the most suited derivatives for *h*CA XII inhibition are those with a 5-phenyl moiety and with polar tails such as the carboxylic acid derivative **5b** (*K*_i_ = 34.2 nM) and mercapto derivative **5g** (*K*_i_ = 39.2 nM).

#### 2.2.3 In Vivo Biological Evaluations

##### 2.2.3.1 Analgesic Activity

The analgesic activities of compounds **5b** and **5d** were assessed over a 90 min period to evaluate both their efficacy and duration of action. As illustrated in Figure 3, both compounds produced a pronounced and statistically significant increase in latency time relative to the vehicle-treated control group (*P* < 0.001).

**Figure 3:**
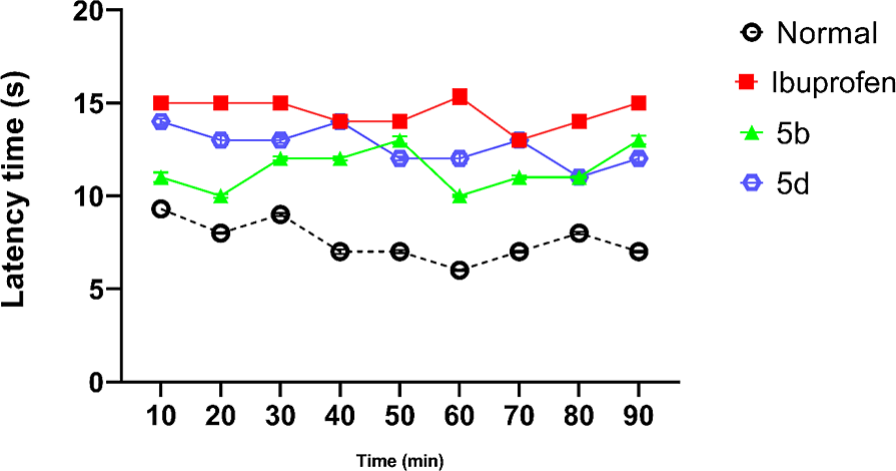
Time-dependent analgesic effects of compounds **5b** and **5d** were evaluated using the hot plate assay. Ibuprofen served as the standard reference drug. Results are presented as mean ± SEM (*n* = 3). Statistical analysis was performed using two-way repeated-measures ANOVA followed by Tukey’s post hoc test to assess significance. The exact *P* values, mean differences, and 95% confidence intervals for all treatment groups across key time points are provided in Table S2 of the Supporting Information.

Compound **5d** demonstrated a rapid onset of action, with significant analgesic effects observed as early as 10 min postadministration (*P* < 0.01). At this early point, its efficacy was statistically compared to that of the reference drug, ibuprofen (*P* > 0.05, Tukey’s test), indicating a similar pharmacodynamic onset. In contrast, compound **5b** exhibited a relatively slower onset of action, with activity that appeared lower than that of Ibuprofen at the 10 min time point (*P* < 0.01). Nevertheless, a highly significant pharmacological effect (*P* < 0.001) was observed at subsequent time intervals under the present experimental conditions.

Notably, both compounds maintained their analgesic effects throughout the entire observation period. At later time points (60–90 min), both **5b** and **5d** continued to display significantly elevated latency times compared to the control group (*P* < 0.001 at 60 min). Collectively, these findings suggest that compounds **5b** and **5d** may possess promising analgesic activity accompanied by a sustained duration of action compared with the reference nonsteroidal anti-inflammatory drug, ibuprofen. Nevertheless, these observations remain preliminary and should be further validated through expanded pharmacological and mechanistic investigations.

##### 2.2.3.2 Anti-inflammatory Activity

The in vivo anti-inflammatory activities of compounds **5b** and **5d** were investigated using the carrageenan-induced rat paw edema model over a 6 h period, with ibuprofen employed as the reference standard. Subplantar administration of carrageenan produced a progressive localized edema in the negative control group, reaching maximal swelling between 3 and 6 h postinjection.

As depicted in Table 3 and Figure 4, both compounds (50 mg/kg) demonstrated promising anti-inflammatory activity, with an apparent early onset of action under the current experimental conditions. At 2 h post carrageenan injection, compounds **5b** and **5d** significantly reduced paw edema relative to the carrageenan-treated group, whereas the reference drug ibuprofen (25 mg/kg) did not exhibit a statistically significant effect at this time point. The most pronounced anti-inflammatory responses were observed at 3 and 4 h, during which both derivatives markedly attenuated paw edema compared with the carrageenan group and displayed considerable anti-inflammatory activity relative to the standard reference treatment. However, comparisons between the tested compounds and ibuprofen should be interpreted cautiously due to differences in the administered dose levels.

**Table 3:** Percentage Inhibition of Carrageenan-Induced Paw Edema in Rats Following Treatment with Compounds **5b** and **5d** ^a^

Treatment	Dose (mg/kg)	1 h	2 h	3 h	4 h	5 h	6 h
**Carrageenan**	1%	-	-	-	-	-	-
**Ibuprofen**	25	0%	0%	23.3%***	30.8%***	68.1%***	77.1%***
**5b**	50	23.3%**	22.8%***	49.2%***	56.6%***	73.0%***	72.3%***
**5d**	50	23.6%**	9.8%***	49.2%***	49.2%***	67.9%***	72.3%***

a Data are presented as percentage inhibition (*n* = 3). Statistical significance was determined based on raw paw volume measurements relative to the carrageenan-induced control group using two-way repeated-measures ANOVA followed by Tukey’s post hoc test. Significance levels are indicated as **P < 0.01 and ***P < 0.001.

**Figure 4:**
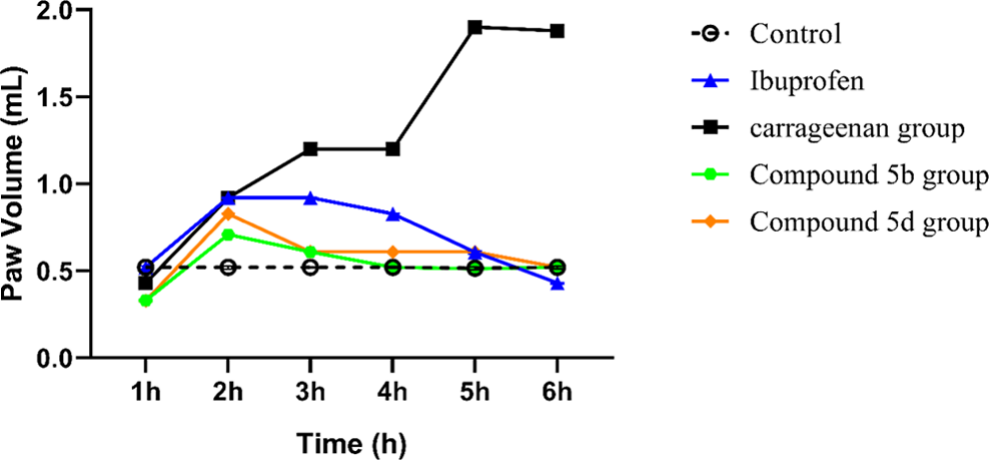
In vivo anti-inflammatory effects of compounds **5b** and **5d** were assessed using the carrageenan-induced rat paw edema model. Paw volume (mL) was recorded at 1, 2, 3, 4, 5, and 6 h following carrageenan administration. Ibuprofen served as the positive reference control. Data are presented as mean ± SEM (*n* = 3). Statistical analysis was conducted using two-way repeated-measures ANOVA followed by Tukey’s multiple comparisons test. The exact *P* values, mean differences (effect size), and 95% confidence intervals for all treatment groups across key time points are provided in Table S3 of the Supporting Information.

This potential anti-inflammatory effect was sustained for the duration of the experiment. At 5 and 6 h, both compounds maintained paw volumes approaching those of the baseline normal control group. Comparative analysis suggested that compound **5b** produced a somewhat greater inhibitory effect on edema at 5 h than compound **5d**; nevertheless, both derivatives demonstrated sustained anti-inflammatory activity throughout the experimental period. Under the present experimental conditions, the tested compounds (50 mg/kg) exhibited anti-inflammatory effects relative to the reference treatment, ibuprofen (25 mg/kg). However, such comparisons should be interpreted cautiously owing to the differences in the administered dose levels.

##### 2.2.3.3 Ulcerogenic Activity

The gastric safety of compounds **5b** and **5d** was systematically assessed over a 24 h period and benchmarked against the reference drug, ibuprofen. As illustrated in Table 4 and Figures 5 and S75, ibuprofen induced a progressive, time-dependent increase in gastric mucosal injury, with the Ulcer Index (UI) rising from 7.33 at 1 h to a maximum of 19.66 at 24 h.

**Table 4:** Assessment of the Ulcerogenic Potential and Gastroprotective Efficacy of Ibuprofen in Comparison with Compounds **5b** and **5d** in Rats across Multiple Time Intervals

Compound	Time (h)	Number of animals with ulcers	% Incidence divided by 10	Average number of ulcers	Average severity	Ulcer Index	% Protection (vs Ibuprofen)
**Control**	1	0/6	0	0	0	0	-
**Ibuprofen**	1	3/6	5	0.67	1.66	7.33	-
**5b**	1	1/6	1.76	0.33	2	4.09	44.20
**5d**	1	0/6	0	0	0	0	100.00
**Control**	3	0/6	0	0	0	0	-
**Ibuprofen**	3	5/6	8.33	2.6	2.8	13.73	-
**5b**	3	2/6	3.33	1.5	2	6.83	50.25
**5d**	3	1/6	1.66	2	2	5.66	58.78
**Control**	6	0/6	0	0	0	0	-
**Ibuprofen**	6	6/6	10	5	3.16	18.16	-
**5b**	6	3/6	5	2.66	2.33	9.99	44.99
**5d**	6	3/6	5	2.33	2	9.33	48.62
**Control**	24	0/6	0	0	0	0	-
**Ibuprofen**	24	6/6	10	5.66	4	19.66	-
**5b**	24	4/6	6.67	3.25	2.75	12.67	35.55
**5d**	24	3/6	5	2.36	2.5	9.86	49.85

**Figure 5:**
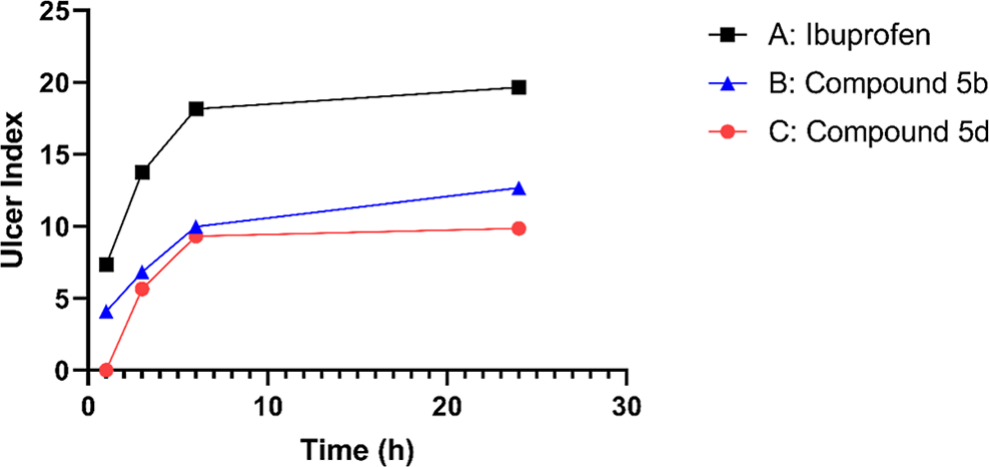
Time-dependent profile of ulcerogenic activity following administration of ibuprofen, **5b**, and **5d** in rats. The Ulcer Index (UI) was determined by integrating the incidence, number, and severity of gastric lesions at 1, 3, 6, and 24 h post-treatment. Each data point represents the mean ulcer index for the respective experimental group (*n* = 6).

In contrast, both synthesized derivatives exhibited a markedly lower ulcerogenic potential. Compound **5d**, in particular, demonstrated pronounced gastroprotective properties, with complete protection observed at 1 h (100% inhibition of ulcer formation). Notably, even after 24 h, compound **5d** maintained a relatively low UI of 9.86, corresponding to a 49.8% reduction in gastric damage compared with ibuprofen.

Similarly, compound **5b** attenuated gastric irritation, affording 50.2% protection at 3 h and 35.5% protection at the 24 h time point. Collectively, these findings indicate that both derivatives possess improved gastric safety profiles relative to the reference drug, with compound **5d** exhibiting superior protective efficacy. Although the obtained results suggest notable biological activity, definitive conclusions regarding the superiority or equivalence to ibuprofen cannot be established at this stage.

##### 2.2.3.4 Antiglaucoma Activity

Compounds **5b** and **5d** were selected for antiglaucoma evaluation based on a balanced dual-enzymatic target profile rather than optimization toward a single enzyme. Given that COX-2 inhibition represents a key determinant of anti-inflammatory activity in this study, priority was assigned to compounds exhibiting superior COX-2 potency and selectivity while maintaining an acceptable carbonic anhydrase II activity.

The intraocular pressure (IOP)-lowering effects of compounds **5b** and **5d** were evaluated by using a rabbit model of glaucoma. Control experiments were conducted by intravitreal injection of 0.05 mL of a hypertonic saline solution (5% NaCl in distilled water) into both eyes. Rabbits with induced elevated IOP received topical administration of 0.05 mL of compound **5b** prepared as 1% solution and 0.1 mL of compound **5d** prepared as 0.05% solution.

Compounds **5b** and **5d** demonstrated intraocular pressure (IOP)-lowering activity in the rabbit model of induced glaucoma, supporting their potential ocular hypotensive effects. Following a substantial increase in IOP after glaucoma induction, topical administration of compound **5b** produced a rapid and significant reduction in IOP, with a noticeable effect observed at 60 min and sustained over the experimental period. The statistically significant difference compared with the sham-treated eye indicates a promising pharmacological activity of this derivative in the tested model.

In contrast, compound **5d** exhibited a slower and less pronounced effect on the IOP. Although a gradual reduction was observed at later time points, the changes did not reach statistical significance, indicating a comparatively weaker efficacy under the tested conditions. Overall, these findings suggest that compound **5b** possesses superior IOP-lowering activity and represents a more promising candidate for further investigation (Figure 6).

**Figure 6:**
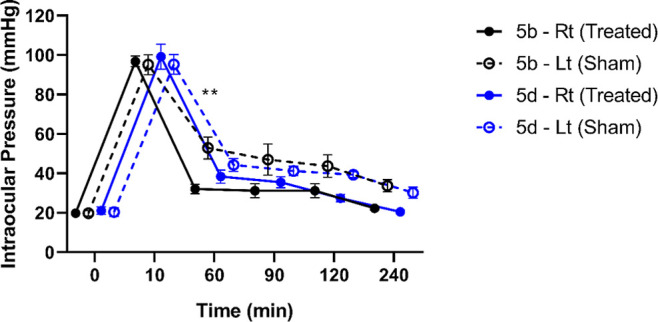
Time-course profile of intraocular pressure (IOP) in a rabbit model of induced glaucoma was evaluated following a single topical administration of derivatives **5b** (black) and **5d** (blue). Data are presented as mean ± SEM (*n* = 4). Solid lines with closed circles denote treated (Rt) eyes, whereas dashed lines with open circles indicate contralateral sham-treated (Lt) eyes. Statistical analysis was performed using two-way ANOVA followed by Bonferroni’s multiple comparisons test. Derivative **5b** induced a significant reduction in IOP relative to the sham-treated eye at 60 min (**P = 0.0061). The exact *P* values, mean differences (effect size), and 95% confidence intervals for all treatment groups across key time points are provided in Table S4 of the Supporting Information.

The present investigation establishes an expanded in vivo pharmacological profile. Compounds **5b** and **5d** demonstrated potent and selective COX-2 inhibition together with significant *h*CA II inhibitory activity, indicating a well-balanced dual-target mechanism. In addition, they exhibited a promising anti-inflammatory, analgesic, with reduced gastric toxicity relative to ibuprofen. Notably, compound **5b** also exhibited significant intraocular pressure-lowering activity in vivo. Collectively, these findings highlight a broader therapeutic potential compared with previously reported pyrazole–sulfonamide derivatives, integrating efficacy with improved safety.

### 2.3 X-ray Crystallographic Analysis

We solved the X-ray crystal structure of *h*CA II bound to derivative **10d** to understand the molecular basis of the CA inhibition. Initial refinement of the structure revealed a well-defined electron density for the inhibitor inside the active site (Figure S54), demonstrating that the benzenesulfonamide group engages the zinc ion in its characteristic deprotonated coordination, typical of this class of inhibitors (Figure 7).^24,25^ In addition, this moiety is stabilized by a hydrogen bond to the Thr199 backbone and hydrophobic contacts with Val121 and Leu198.^24,25^ The amino group in the pyrazole ring contributes to stabilizing the interaction with a hydrogen bond with Thr200. Finally, the thioallyl tail, which exhibits a weaker and less defined electron density, likely because of increased conformational flexibility, is directed into a hydrophobic pocket through an interaction with Phe20.

**Figure 7:**
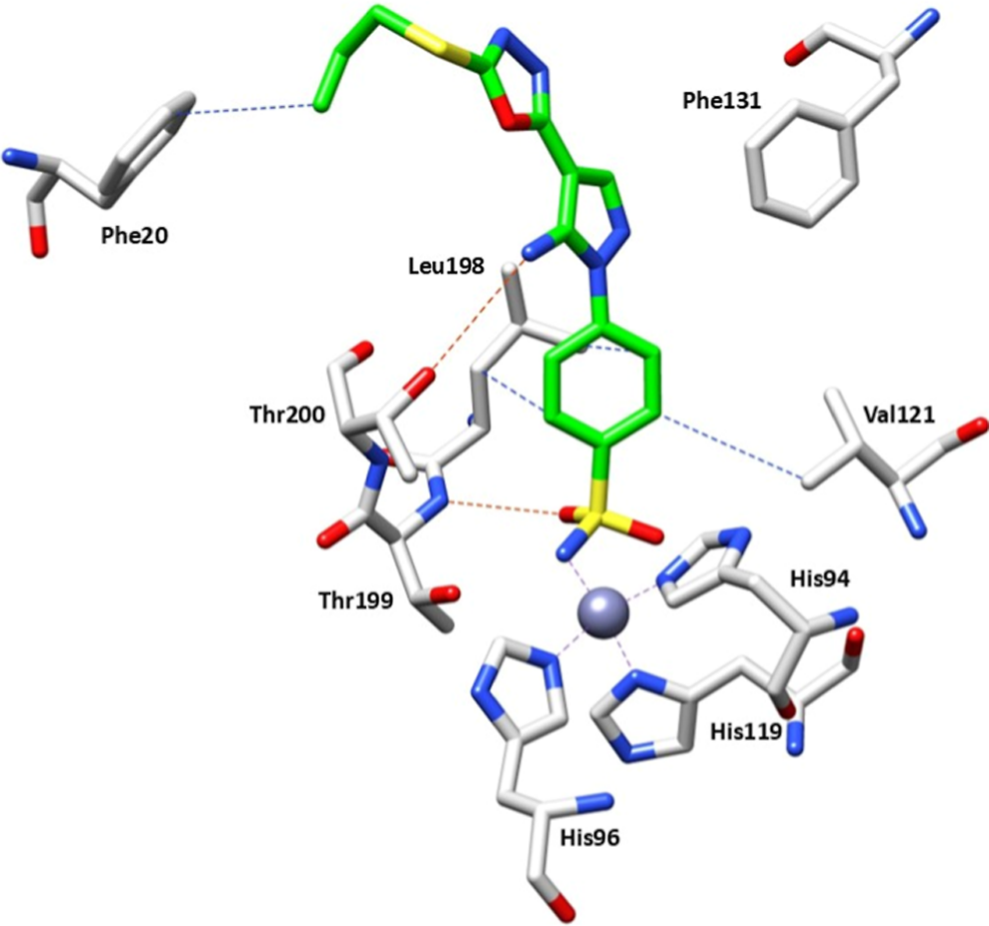
X-ray crystal structure of *h*CA II bound with compound **10d** (green, PDB: 9T4S). Residues involved in the binding of inhibitors are also shown; the gray sphere represents the zinc ion in the active site of the proteins. Van der Waals interactions are shown in blue, and hydrogen bonds are shown in red.

### 2.4 Molecular Modeling

#### 2.4.1 Molecular Docking

Molecular docking was conducted to assess the binding interactions of **5b** and **5d** within *h*CA II and COX-2 binding sites using the Glide docking tool. These two compounds were selected for modeling to provide qualitative, hypothesis-generating support for their experimental inhibitory activity. The docking approach was validated by demonstrating its capacity to reproduce the binding modes of the cocrystallized *h*CA II inhibitor (furosemide in PDB 1Z9Y) and the COX-2 inhibitor (rofecoxib in PDB 5KIR). This was performed by superimposing the crystallographic poses with their top-scored docking poses. The docked poses of furosemide and rofecoxib successfully resembled their crystallographic binding modes with RMSD values of 0.95 and 0.27 Å, respectively, providing impetus to initiate the docking of acetazolamide, celecoxib, **5b**, and **5d** using the same settings. The Glide docking scores of the top-ranked poses of acetazolamide, celecoxib, **5b**, and **5d** docked into *h*CA II and COX-2 are summarized in Table 5.

**Table 5:** Glide Docking Scores Calculated for Top-Ranked Poses in *h*CA II and COX-2 Active Sites

	Glide docking scores (kcal/mol)	
Compound	*h*CA II	COX-2
**Acetazolamide**	–7.30	NA
**Celecoxib**	NA	–9.60
**5b**	–8.05	–9.18
**5d**	–7.48	–10.10

Notably, compounds **5b** and **5d** demonstrated docking scores comparable to those of the reference ligands in both of the enzymes. These docking results were consistent with the observed in vitro inhibitory activity; for instance, compound **5b**, which showed relatively stronger inhibition of *h*CA II, also yielded a more favorable docking score compared to **5d**. Conversely, compound **5d** demonstrated a superior docking score in COX-2, in agreement with its favorable in vitro COX-2 inhibitory activity. These computational predictions support the experimental inhibitory data (Table 5).

Figure 8 illustrates the docked poses of acetazolamide in *h*CA II and celecoxib in COX-2, compared with the top-ranked poses of compounds **5b** and **5d** in both proteins. In *h*CA II, the Zn^2+^ ion exhibited a tetrahedral coordination with the deprotonated sulfonamide nitrogen of acetazolamide, **5b**, and **5d** as well as His94, His96, and His119 through their imidazole nitrogen atoms. Additionally, one sulfonamide oxygen atom in all three compounds formed bifurcated hydrogen bonds with the backbone NH groups of Thr199 and Thr200. The deprotonated NH of acetazolamide was anchored to the hydroxyl group of the Thr199 side-chain via a hydrogen bond, while the thiadiazole moiety was positioned near the hydroxyl group of Thr200, suggesting the possible formation of an additional hydrogen bond. However, in compounds **5b** and **5d**, the pyrazole nitrogen was oriented toward Gln92, forming a hydrogen bond with its side-chain amide NH, while the nitrogen atom of the neighboring oxadiazole ring established an additional hydrogen bond with the side-chain amide NH of Asn67. Furthermore, the phenyl ring attached to the pyrazole moiety in both compounds was positioned adjacent to Phe131, suggesting π–π stacking interactions comparable to that observed for the furan ring of furosemide.

**Figure 8:**
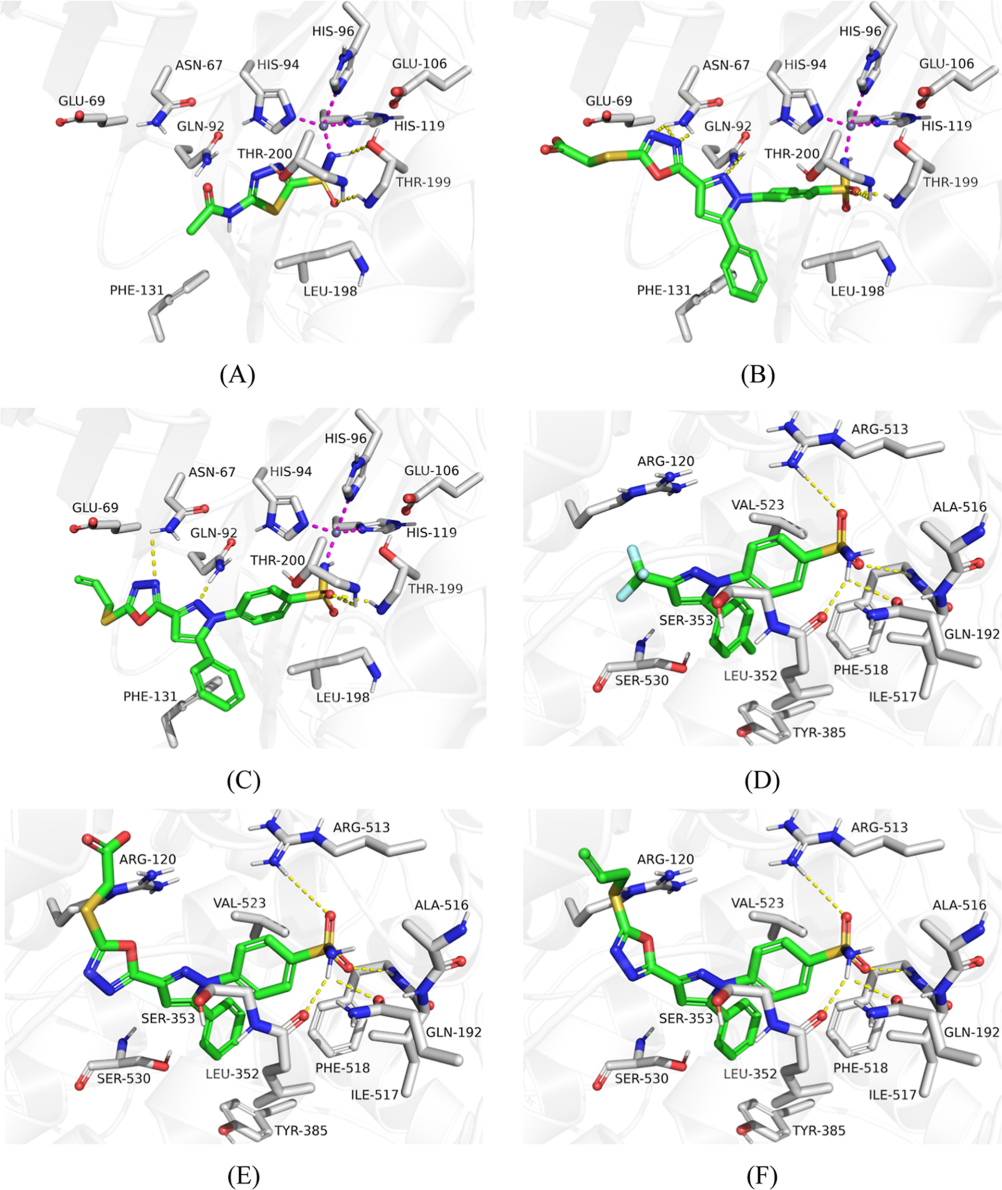
Detailed view of amino acid interactions within the binding sites of *h*CA II (PDB ID: 1Z9Y) and COX-2 (PDB ID: 5KIR) with reference ligands, **5b** and **5d**. (A) *h*CA II–**acetazolamide**, (B) *h*CA II–**5b**, (C) *h*CA II–**5d**, (D) COX-2–**celecoxib**, (E) COX-2–**5b**, and (F) COX-2–**5d**. Hydrogen bonds (≤3.0 Å) are depicted as yellow dashed lines, while Zn^2+^ coordination is depicted as magenta dashed lines.

For compound **5b**, the closest atom–centroid distance is 3.8 Å and the centroid–centroid distance is 5.0 Å, whereas for compound **5d**, the corresponding distances are 3.9 Å and 5.3 Å, respectively. In both compounds, the angle between the ring planes is approximately 80°. These metrics support the formation of T-shaped (edge-to-face) π–π interactions.^26^

In COX-2, the two sulfonamide oxygen atoms of celecoxib, **5b**, and **5d** engaged Arg513 and Phe518 in hydrogen bonding interactions. Additionally, the sulfonamide NH_2_ group of the three compounds formed bifurcated hydrogen bonds with Gln192 and Leu352. In addition, the pyrazole ring of celecoxib as well as the oxadiazole ring of **5b** and **5d** was positioned in proximity to Arg120, indicating possible π–cation interactions. Finally, in all three compounds, the phenyl ring attached to the pyrazole moiety was located within a hydrophobic pocket defined by Leu352, Tyr385, and Val523, suggesting hydrophobic interactions and π–π stacking stabilization.

#### 2.4.2 Molecular Dynamics Simulations

To evaluate the stability of the predicted binding modes obtained from molecular docking, the docking poses in complex with their corresponding receptor were simulated for 200 ns using the Desmond package. Analysis of the simulation trajectories focused on monitoring the Root Mean Square Deviation (RMSD) values and interaction occupancy rates, as illustrated in Figures 9 and 10. The RMSD values of the Cα atoms of *h*CA II and COX-2 fluctuated between 1 and 2.5 Å in all simulated runs. Acetazolamide and celecoxib stabilized at RMSD values of around 1.5–2 Å upon superposition onto the Cα atoms of their cognate receptors. Compound **5b** and **5d** in complex with *h*CA II showed RMSD values ranging between 1 and 2 Å, with few short peaks reaching 3 Å, observed in **5d**. In COX-2, **5b** also maintained RMSD fluctuations between 1 and 2 Å, whereas **5d** displayed slightly higher RMSD values of nearly 2–3 Å (Figure 9).

**Figure 9:**
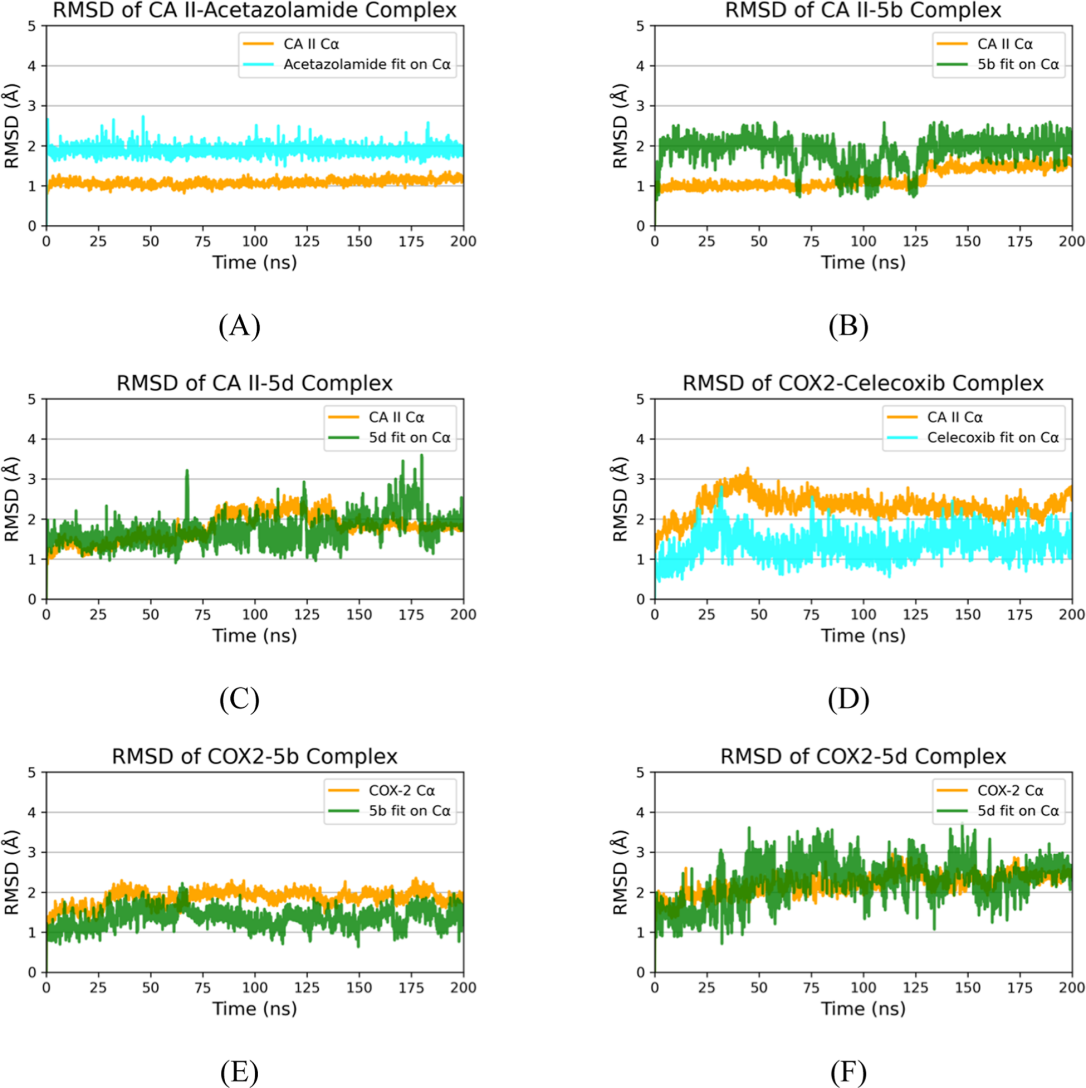
RMSD values throughout 200 ns MD simulations. (A) *h*CA II–**acetazolamide**, (B) *h*CA II–**5b**, (C) *h*CA II–**5d**, (D) COX-2–**celecoxib**, (E) COX-2–**5b**, and (F) COX-2–**5d**.

**Figure 10:**
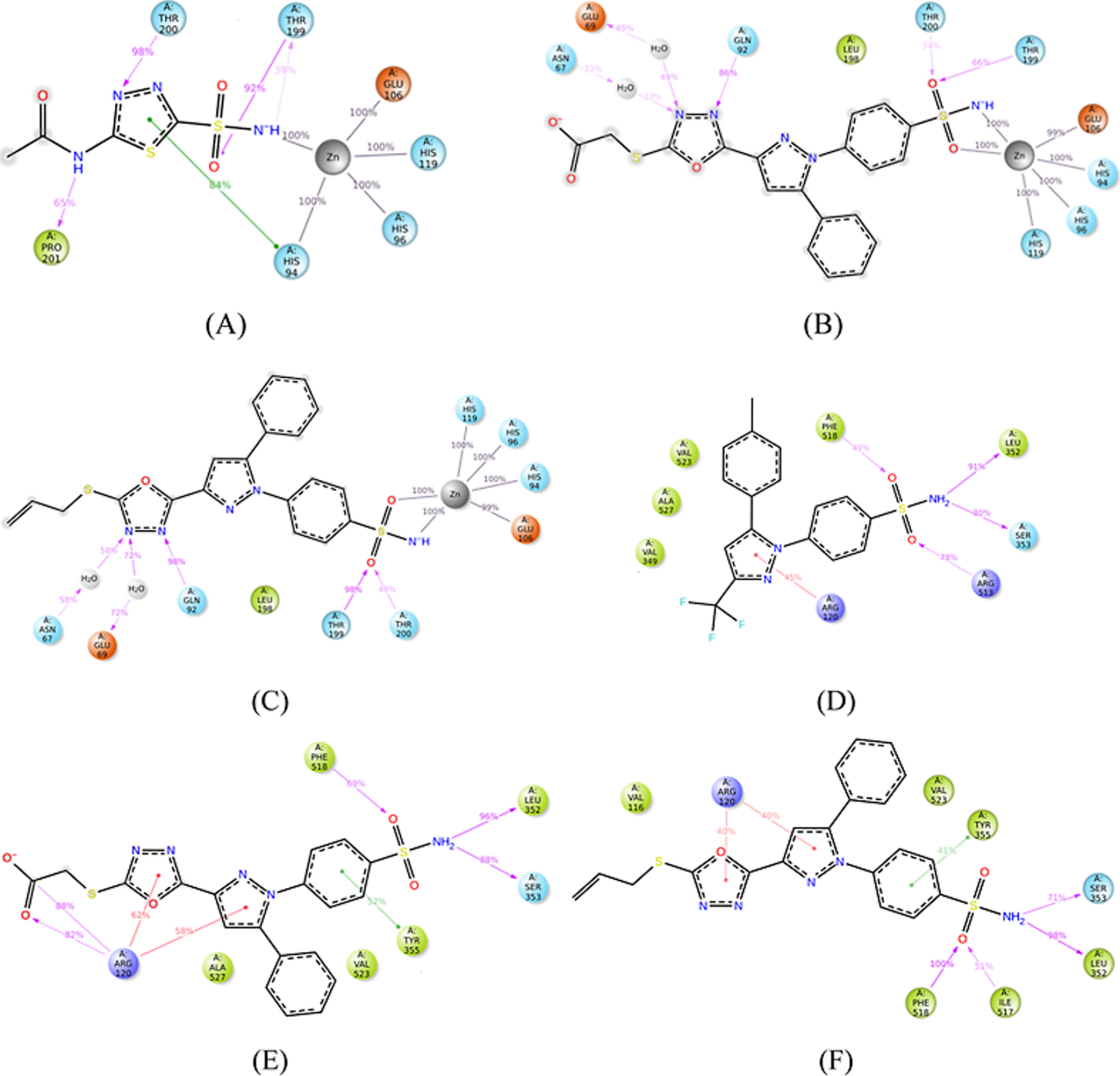
Interaction occupancy rates throughout 200 ns MD simulations. (A) *h*CA II–**acetazolamide**, (B) *h*CA II–**5b**, (C) *h*CA II–**5d**, (D) COX-2–**celecoxib**, (E) COX-2–**5b**, and (F) COX-2–**5d**.

The key interactions with *h*CA II and COX-2 pocket residues observed in molecular docking were largely preserved throughout the simulation trajectories. The Zn^2+^ coordination by the three *h*CA II histidine residues and the deprotonated sulfonamide nitrogen was maintained, while additional coordination interactions were established with the carboxylate oxygen of Glu106. Furthermore, a sustained coordination interaction with the Zn^2+^ atom was observed via one sulfonamide oxygen of compounds **5b** and **5d**, whereas the second sulfonamide oxygen maintained the bifurcated hydrogen bond with Thr199 and Thr200, with occupancy rates between 34% and 98%. In acetazolamide, the two hydrogen bonds formed of the sulfonamide oxygen and the NH group with Thr199 were preserved for 92% and 38% of the simulation time, respectively. In addition, the pyrazole nitrogen and its attached amide NH adopted a favorable orientation toward Thr200 and Thr201, forming two hydrogen bonds that lasted for 98% and 66% of the simulation time, respectively. Similarly, the hydrogen bond with Gln92, mediated by the oxadiazole nitrogen atoms of compounds **5b** and **5d**, was maintained for 86% and 98% of the simulation time, respectively. However, the direct hydrogen bond with Asn67 observed in both compounds was disrupted during the simulation and replaced by two water-mediated hydrogen bonds involving Asn67 and Glu69. Additionally, the π–π stacking interaction with Phe131 observed in all three compounds was not consistently maintained during the simulation, whereas a hydrophobic interaction with Leu198 was observed and remained stable (Figure 10A,B).

In COX-2, the interactions of celecoxib, **5b**, and **5d** with Leu352, Ser353, Ile517, Phe518 (hydrogen bonds), and Arg120 (π–cation) were preserved throughout the simulation runs, with occupancy rates ranging from 40% to 100%. In compound **5b**, two additional hydrogen bonds formed between the carboxylate oxygens of the tail and Arg120 were observed during the simulation. In both compounds, a π–π stacking interaction was established between the phenyl ring of the benzenesulfonamide moiety and Tyr355 and was maintained for approximately half of the simulation time (Figure 10D–F). These simulation results suggest that the proposed binding modes of compounds **5b** and **5d** within the *h*CA II and COX-2 active sites are stable and offer a molecular explanation for their observed structure–activity relationships.

## 3 Conclusion

A series of novel sulfonamide derivatives **5a–g** and **10a–e** were designed, synthesized, and biologically evaluated for their anti-inflammatory activity. In vitro cyclooxygenase (COX-1 and COX-2) inhibitory assays were performed using celecoxib as a reference drug, and most of the synthesized compounds demonstrated enhanced COX-2 selectivity over COX-1. Notably, compounds **5b**, **5c**, **5d**, **5g**, **10c**, and **10e** exhibited promising COX-2 inhibitory activity (IC_50_ = 0.05–0.17 μM) compared to celecoxib (IC_50_ = 0.05 μM). Among them, compounds **5b** and **5d** emerged as the most promising candidates, displaying high selectivity indices (SI = 9.25 and 12.02, respectively). Moreover, carbonic anhydrase inhibition studies demonstrated that compound **5b** acted as a selective inhibitor of the *h*CA II isoform. Based on their superior in vitro profiles, compounds **5b** and **5d** were further evaluated in vivo, and the results demonstrated promising analgesic and anti-inflammatory activities, characterized by a relatively rapid onset and prolonged duration of action. Importantly, ulcerogenic studies demonstrated a markedly improved gastric safety profile for **5b** and **5d**; however, these findings are considered as preliminary and require further pharmacological confirmation. Collectively, compounds **5b** and **5d** demonstrated encouraging pharmacological profiles under the current experimental conditions. Nevertheless, additional comprehensive studies are necessary to validate these preliminary findings and to further establish their therapeutic potential, safety profile, and underlying mechanisms of action. Furthermore, in a rabbit model of glaucoma, compound **5b** exhibited a statistically significant and sustained reduction in intraocular pressure, surpassing that of compound **5d** and underscoring its potential as an ocular hypotensive agent. However, the absence of experimental evaluation against the *h*CA IV isoform, which is known to contribute to aqueous humor regulation and represents a relevant target in glaucoma therapy, should be acknowledged as a limitation of the present study. Therefore, the antiglaucoma interpretation and the proposed ocular hypotensive mechanism of compound **5b** require further validation through *h*CA IV inhibition studies.

Finally, integrated molecular docking and molecular dynamics studies were performed to gain insights into the binding conformations and key molecular interactions between the synthesized compounds and the target enzymes. In summary, compound **5b** exhibited favorable efficacy and safety profiles, with enhanced antiglaucoma activity.

## 4 Experimental Section

### 4.1 Chemistry

All reactions were monitored by TLC using silica gel F_254_ plates (Merck, Germany), and the spots were observed using a UV light with a wavelength of 254 nm. The NMR spectra were recorded on Bruker in deuterated chloroform CDCl_3_ or deuterated dimethylsulfoxide (DMSO-*d*_6_) at 400 MHz for ^1^H NMR and 100 MHz for ^13^C MR on Bruker AVANCE III 400 MHz FT-NMR spectrometers, the Microanalytical Unit, Faculty of Pharmacy, Cairo University. Chemical Shifts were quoted in δ as parts per million (ppm). As for the proton magnetic resonance, D_2_O was carried out for NH and OH exchangeable protons. HPLC purity was measured by UV absorbance at 254 nm using MeOH/H_2_O/0.05% TFA. The HPLC consisted of a LiChrosorb RP-18 (5 μm) 100-4.6 Merck column (Merck, Darmstadt, Germany), two LC-10AD pumps, an SPD-M10A VP PDA detector, and an SIL-HT autosampler, all from the manufacturer Shimadzu (Kyoto, Japan). All compounds are >95% pure by HPLC analysis. The absorption spectra were recorded with an SPD-M10A diode array detector Shimadzu spectrophotometer (Kyoto, Japan). HRMS-ESI (high-resolution mass spectrometry) was measured on an Orbitrap Fusion Tribrid mass spectrometer (Thermo Fisher Scientific, San Jose, CA, USA).

#### 4.1.1 General

The following intermediates have been synthesized using the previously mentioned procedures:^12,22^ ethyl 2-benzoyl-3-(dimethylamino)­acrylate (**2**), ethyl 5-phenyl-1-(4-sulfamoylphenyl)-1*H*-pyrazole-4-carboxylate (**3**), 4-(4-(hydrazinecarbonyl)-5-phenyl-1*H*-pyrazol-1-yl)­benzenesulfonamide (**4**), ethyl 2-cyano-3-ethoxyacrylate (**7**), ethyl 5-amino-1-(4-sulfamoylphenyl)-1*H*-pyrazole-4-carboxylate (**8**), and 4-(5-amino-4-(hydrazinecarbonyl)-1*H*-pyrazol-1-yl)­benzenesulfonamide (**9**). All instruments and spectral data are included in the Supporting Information (S7–S64).

#### 4.1.2 General Procedure for Synthesis of Compounds **5a–g** and **10a–e**

In a 50 mL round flask, acid hydrazide 4 or 9 (1.0 mmol) was added to DMF (2.0 mL), followed by the addition of carbon disulfide (3.0 mmol), and stirred for 15 min at room temperature. Then, the reaction mixture temperature was raised to 70 °C for 4 h until the oxadiazole ring closure was complete; derivative **5g** was afforded. After that, the reaction mixture was cooled to room temperature, and triethylamine (4 mmol) was added. The alkyl halide or bromoacetic acid (1.2 mmol) was then added and stirred overnight at room temperature. The reaction was monitored using TLC with the elution system dichloromethane/methanol. The reaction mixture was poured on ice/water, and the precipitate formed was filtered and recrystallized from methanol.

##### 4.1.2.1 4-{4-[5-(Benzylthio)-1,3,4-oxadiazol-2-yl]-5-phenyl-1*H*-pyrazol-1-yl}­benzenesulfonamide (**5a**)

Yield: 72%; ^1^H NMR (400 MHz, DMSO-*d*_6_): δ 4.36 (s, 2H, –CH_2_−), 7.29 (s, 5H, Ar-H), 7.45–7.52 (m, 9H, 7H of Ar-H and 2H of NH_2_, D_2_O exchangeable), 7.81 (d, *J* = 8.52 Hz, 2H, Ar-H), 8.42 (s, 1H, Ar-H); ^13^C NMR (100 MHz, DMSO-*d*_6_): δ 36.29 (−CH_2_−), 107.35, 126.12 (2C), 127.05 (2C), 128.05, 128.22 (2C), 129.01, 129.04 (2C), 129.33, 130.33, 130.93 (2C), 132.99, 136.88, 140.65, 141.49, 142.98, 143.91, 160.73, 162.54 (Ar-Cs); For C_24_H_19_N_5_O_3_S_2_ (489.57) Calc.: C, 58.88; H, 3.91; N, 14.31. Found: C, 59.04; H, 4.08; N, 14.56; HRMS (ESI) for C_24_H_20_N_5_O_3_S_2_: calcd, 490.1002; found, 490.1003 [M + H]^+^. HPLC: rt 14.03 min (purity 96.66%).

##### 4.1.2.2 2-{(5-[5-Phenyl-1-(4-sulfamoylphenyl)-1*H*-pyrazol-4-yl]-1,3,4-oxadiazol-2-yl)­thio}­acetic Acid (**5b**)

Yield: 63%; ^1^H NMR (400 MHz, DMSO-*d*_6_): δ 3.79 (s, 2H, –CH_2_), 7.42–7.47 (m, 10H, 7H of Ar-H and 3H of OH and NH_2_, D_2_O exchangeable), 7.81 (d, *J* = 8.0 Hz, 2H, Ar-H), 8.42 (s, 1H, Ar-H); ^13^C NMR (100 MHz, DMSO-*d*_6_): δ 45.69 (−CH_2_−), 107.50, 126.09 (2C), 127.04 (2C), 128.04, 128.95 (2C), 130.23, 130.89 (2C), 140.63, 141.54, 142.84, 143.88, 160.04, 163.96 (Ar-Cs), 168.93 (C=O); For C_19_H_15_N_5_O_5_S_2_ (457.48) Calc.: C, 49.88; H, 3.30; N, 15.31. Found: C, 50.06; H, 3.47; N, 15.54; HRMS (ESI) for C_19_H_16_N_5_O_5_S_2_: calcd, 458.0587; found, 458.0590 [M + H]^+^. HPLC: rt 10.46 min (purity 94.55%).

##### 4.1.2.3 4-{5-Phenyl-4-[5-(propylthio)-1,3,4-oxadiazol-2-yl]-1*H*-pyrazol-1-yl}­benzenesulfonamide (**5c**)

Yield: 86%; ^1^H NMR (400 MHz, DMSO-*d*_6_): δ 0.90 (t, *J* = 7.3 Hz, 3H, –SCH_2_CH_2_CH_3_), 1.60 (h, *J* = 7.2 Hz, 2H, −SCH_2_CH_2_CH_3_), 3.05 (t, *J* = 7.1 Hz, 2H, −SCH_2_CH_2_CH_3_), 7.41–7.49 (m, 9H, 7H of Ar-H and 2H of NH_2_, D_2_O exchangeable), 7.81 (d, *J* = 8.6 Hz, 2H, Ar-H), 8.43 (s, 1H, Ar-H); ^13^C NMR (100 MHz, DMSO-*d*_6_): δ 13.18 (propyl-C), 22.93 (propyl-C), 34.27 (propyl-C), 107.49, 126.07 (2C), 127.05 (2C), 128.10, 128.99 (2C), 130.26, 130.86 (2C), 140.6, 141.5, 142.93, 143.90, 160.52, 163.19 (Ar-Cs); For C_20_H_19_N_5_O_3_S_2_ (441.52) Calc.: C, 54.41; H, 4.34; N, 15.86. Found: C, 54.60; H, 4.58; N, 16.02; HRMS (ESI) for C_20_H_20_N_5_O_3_S_2_: calcd, 442.1002; found, 442.1004 [M + H]^+^. HPLC: rt 12.89 min (purity 95.08%).

##### 4.1.2.4 4-{4-[5-(Allylthio)-1,3,4-oxadiazol-2-yl]-5-phenyl-1*H*-pyrazol-1-yl}­benzenesulfonamide (**5d**)

Yield: 74%; ^1^H NMR (400 MHz, DMSO-*d*_6_): δ 3.74 (d, *J* = 6.92 Hz, 2H, −SCH_2_CH=CH_2_), 5.07–5.12 (m, $1\frac{1}{2}H$ , –SCH_2_CH=CH_2_), 5.16 (d, *J* = 0.96 Hz, 1/2H, –SCH_2_CH=CH_2_), 5.80–5.87 (m, 1H, −SCH_2_CH=CH_2_), 7.44–7.48 (m, 9H, 7H of Ar-H and 2H of NH_2_, D_2_O exchangeable), 7.81 (d, *J* = 8.6 Hz, 2H, Ar-H), 8.45 (s, 1H, Ar-H); ^13^C NMR (100 MHz, DMSO-*d*_6_): δ 35.06 (allyl-C), 107.42, 119.58 (allyl-C), 126.11 (2C), 127.05 (2C), 128.08, 129.0 (2C), 130.30, 130.91 (2C), 133.14 (allyl-C), 140.64, 141.50, 142.98, 143.92, 160.79, 162.41 (Ar-Cs); For C_20_H_17_N_5_O_3_S_2_ (439.51) Calc.: C, 54.65; H, 3.90; N, 15.93. Found: C, 54.92; H, 3.69; N, 16.09; HRMS (ESI) for C_20_H_18_N_5_O_3_S_2_: calcd, 440.0845; found, 440.0848 [M + H]^+^. HPLC: rt 12.34 min (purity 96.39%).

##### 4.1.2.5 4-{4-[5-(Butylthio)-1,3,4-oxadiazol-2-yl]-5-phenyl-1*H*-pyrazol-1-yl}­benzenesulfonamide (**5e**)

Yield: 82%; ^1^H NMR (400 MHz, DMSO-*d*_6_): δ 0.85 (t, *J* = 6.72 Hz, 3H, –SCH_2_CH_2_CH_2_CH_3_), 1.30–1.35 (m, 2H, –SCH_2_CH_2_CH_2_CH_3_), 1.58 (t, *J* = 6.56 Hz, 2H, −SCH_2_CH_2_CH_2_CH_3_), 3.07 (t, *J* = 6.44 Hz, 2H, −SCH_2_CH_2_CH_2_CH_3_), 7.43–7.47 (m, 9H, 7H of Ar-H and 2H of NH_2_, D_2_O exchangeable), 7.81 (d, *J* = 7.8 Hz, 2H, Ar-H), 8.43 (s, 1H, Ar-H); ^13^C NMR (100 MHz, DMSO-*d*_6_): δ 13.75 (butyl-C), 21.41 (butyl-C), 31.49 (butyl-C), 32.1 (butyl-C), 107.47, 126.07 (2C), 127.05 (2C), 128.08, 128.98 (2C), 130.28, 130.85 (2C), 140.57, 141.50, 142.92, 143.88, 160.51, 163.22 (Ar-Cs); For C_21_H_21_N_5_O_3_S_2_ (455.55) Calc.: C, 55.37; H, 4.65; N, 15.37. Found: C, 55.29; H, 4.59; N, 15.58; HRMS (ESI) for C_21_H_22_N_5_O_3_S_2_: calcd, 456.1158; found, 456.1160 [M + H]^+^. HPLC: rt 13.66 min (purity 100%).

##### 4.1.2.6 4-{4-[5-(Isobutylthio)-1,3,4-oxadiazol-2-yl]-5-phenyl-1*H*-pyrazol-1-yl}­benzenesulfonamide (**5f**)

Yield: 88%; ^1^H NMR (400 MHz, DMSO-*d*_6_): δ 0.90 (d, *J* = 6.12 Hz, 6H, –SCH_2_CH­(CH_3_)_2_), 1.81–1.85 (m, 1H, −SCH_2_CH­(CH_3_)_2_), 2.97 (d, *J* = 6.24 Hz, 2H, −SCH_2_CH­(CH_3_)_2_), 7.43–7.47 (m, 9H, 7H of Ar-H and 2H of NH_2_, D_2_O exchangeable), 7.81 (d, *J* = 7.88 Hz, 2H, Ar-H), 8.44 (s, 1H, Ar-H); ^13^C NMR (100 MHz, DMSO-*d*_6_): δ 21.57 (isobutyl-2C), 28.56, 40.58 (isobutyl-2C), 107.45, 126.09 (2C), 127.04 (2C), 128.08, 129.01 (2C), 130.30, 130.86 (2C), 140.59, 141.49, 142.94, 143.89, 160.50, 163.33; For C_21_H_21_N_5_O_3_S_2_ (455.55) Calc.: C, 55.37; H, 4.65; N, 15.37. Found: C, 55.48; H, 4.74; N, 15.60; HRMS (ESI) for C_21_H_22_N_5_O_3_S_2_: calcd, 456.1158; found, 456.1161 [M + H]^+^. HPLC: rt 13.54 min (purity 100%).

##### 4.1.2.7 4-{4-(5-Mercapto-1,3,4-oxadiazol-2-yl)-5-phenyl-1*H*-pyrazol-1-yl}­benzenesulfonamide (**5g**)

Yield: 86%; ^1^H NMR (400 MHz, DMSO-*d*_6_): δ 7.40–7.46 (m, 9H, 7H of Ar-H and 2H of NH_2_, D_2_O exchangeable), 7.80 (d, *J* = 8.6 Hz, 2H, Ar-H), 8.41 (s, 1H, Ar-H), 14.51 (s, 1H, SH, D_2_O exchangeable); ^13^C NMR (100 MHz, DMSO-*d*_6_): δ 106.80, 126.18 (2C), 127.05 (2C), 127.76, 129.02 (2C), 130.36, 130.82 (2C), 140.62, 141.41, 143.09, 143.99, 156.47, 177.13 (Ar-Cs); For C_17_H_13_N_5_O_3_S_2_ (399.44) Calc.: C, 51.12; H, 3.28; N, 17.53. Found: C, 51.35; H, 3.40; N, 17.79. HPLC: rt 10.58 min (purity 95.37%).

##### 4.1.2.8 4-{5-Amino-4-[5-(benzylthio)-1,3,4-oxadiazol-2-yl]-1*H*-pyrazol-1-yl}­benzenesulfonamide (**10a**)

Yield: 72%; ^1^H NMR (400 MHz, DMSO-*d*_6_): δ 4.50 (s, 2H, −CH_2_−), 6.52 (s, 2H NH_2_, D_2_O exchangeable), 7.23–7.36 (m, 3H, Ar-H), 7.43 (d, *J* = 7.5 Hz, 2H, Ar-H), 7.50 (s, 2H, NH_2_, D_2_O exchangeable), 7.79 (dd, *J* = 8.6, 2.3 Hz, 2H, Ar-H), 7.94 (td, *J* = 7.7, 2.2 Hz, 3H, Ar-H); ^13^C NMR (100 MHz, DMSO-*d*_6_): δ 36.52 (−CH_2_−), 89.19, 123.90 (2C), 127.52 (2C), 128.22, 129.06 (2C), 129.52 (2C), 137.21, 139.04, 140.83, 143.10, 147.52, 160.31, 161.58 (Ar-Cs); For C_18_H_16_N_6_O_3_S_2_ (428.49) Calc.: C, 50.45; H, 3.76; N, 19.61. Found: C, 50.32; H, 3.94; N, 19.89; HRMS (ESI) for C_18_H_17_N_6_O_3_S_2_: calcd, 429.0798; found, 429.0801 [M + H]^+^. HPLC: rt 12.82 min (purity 97.21%).

##### 4.1.2.9 2-{(5-[5-Amino-1-(4-sulfamoylphenyl)-1*H*-pyrazol-4-yl]-1,3,4-oxadiazol-2-yl)­thio}­acetic Acid (**10b**)

Yield: 63%; ^1^H NMR (400 MHz, DMSO-*d*_6_): δ 4.18 (s, 2H, −CH_2_−), 6.53 (s, 2H, NH_2_, D_2_O exchangeable), 7.50 (s, 2H, NH_2_, D_2_O exchangeable), 7.82 (d, *J* = 8.7 Hz, 2H, Ar-H), 7.93–8.00 (m, 3H, Ar-H), 13.16 (s, 1H, OH, D_2_O exchangeable); ^13^C NMR (100 MHz, DMSO-*d*_6_): δ 34.79 (−CH_2_−), 89.12, 123.91 (2C), 127.51 (2C), 139.0, 140.83, 143.12, 147.50, 160.32, 161.44 (Ar-Cs), 169.50 (C=O); For C_13_H_12_N_6_O_5_S_2_ (396.40) Calc.: C, 39.39; H, 3.05; N, 21.20. Found: C, 39.60; H, 3.21; N, 21.04; HRMS (ESI) for C_13_H_13_N_6_O_5_S_2_: calcd, 397.0383; found, 397.0385 [M + H]^+^. HPLC: rt 9.04 min (purity 99.66%).

##### 4.1.2.10 4-{5-Amino-4-[5-(propylthio)-1,3,4-oxadiazol-2-yl]-1*H*-pyrazol-1-yl}­benzenesulfonamide (**10c**)

Yield: 86%; ^1^H NMR (400 MHz, DMSO-*d*_6_): δ 1.01 (t, *J* = 7.4 Hz, 3H, –SCH_2_CH_2_CH_3_), 1.78 (h, *J* = 7.3 Hz, 2H, −SCH_2_CH_2_CH_3_), 3.26 (t, *J* = 7.1 Hz, 2H, −SCH_2_CH_2_CH_3_), 6.54 (s, 2H, NH_2_, D_2_O exchangeable), 7.51 (s, 2H, NH_2_, D_2_O exchangeable), 7.83 (d, *J* = 8.7 Hz, 2H, Ar-H), 7.96–8.02 (m, 3H, Ar-H); For C_14_H_16_N_6_O_3_S_2_ (380.45) Calc.: C, 44.20; H, 4.24; N, 22.09. Found: C, 44.39; H, 4.53; N, 22.30; HRMS (ESI) for C_14_H_17_N_6_O_3_S_2_: calcd, 381.0798; found, 381.0800 [M + H]^+^. HPLC: rt 13.68 min (purity 100%).

##### 4.1.2.11 4-{4-[5-(Allylthio)-1,3,4-oxadiazol-2-yl]-5-amino-1*H*-pyrazol-1-yl}­benzenesulfonamide (**10d**)

Yield: 74%; ^1^H NMR (400 MHz, DMSO-*d*_6_): δ 3.94 (dt, *J* = 7.0, 1.1 Hz, 2H, SCH_2_CH=CH_2_), 5.17 (ddd, *J* = 9.9, 1.7, 0.9 Hz, 1H, SCH_2_CH=CH_2_), 5.34 (dq, *J* = 16.9, 1.4 Hz, 1H, SCH_2_CH=CH_2_), 5.99 (ddt, *J* = 17.0, 10.0, 7.0 Hz, 1H, SCH_2_CH=CH_2_), 6.54 (s, 2H, NH_2_, D_2_O exchangeable), 7.51 (s, 2H, NH_2_, D_2_O exchangeable), 7.82 (d, *J* = 8.7 Hz, 2H, Ar-H), 7.98 (d, *J* = 8.7 Hz, 3H, Ar-H); ^13^C NMR (100 MHz, DMSO-*d*_6_): δ 35.38 (allyl-C), 89.24, 119.72 (allyl-C), 123.89 (2C), 127.51 (2C), 133.31 (allyl-C), 139.04, 140.83, 143.11, 147.53, 160.14, 161.64 (Ar-Cs); For C_14_H_14_N_6_O_3_S_2_ (378.43) Calc.: C, 44.43; H, 3.73; N, 22.21. Found: C, 44.67; H, 3.92; N, 22.47; HRMS (ESI) for C_14_H_15_N_6_O_3_S_2_: calcd, 379.0641; found, 379.0643 [M + H]^+^. HPLC: rt 11.49 min (purity 99.34%).

##### 4.1.2.12 4-{5-Amino-4-[5-(ethylthio)-1,3,4-oxadiazol-2-yl]-1*H*-pyrazol-1-yl}­benzenesulfonamide (**10e**)

Yield: 86%; ^1^H NMR (400 MHz, DMSO-*d*_6_): δ 1.41 (t, *J* = 7.3 Hz, 3H, −CH_2_CH_3_), 3.28 (q, *J* = 7.3 Hz, 2H, −CH_2_CH_3_), 6.54 (s, 2H, NH_2_, D_2_O exchangeable), 7.51 (s, 2H, NH_2_, D_2_O exchangeable), 7.80–7.86 (m, 2H, Ar-H), 7.94–8.00 (m, 3H, Ar-H); ^13^C NMR (100 MHz, DMSO-*d*_6_): δ 15.35 (ethyl-C), 27.25 (ethyl-C), 89.28, 123.85 (2C), 127.51 (2C), 139, 140.85, 143.09, 147.5, 160.84, 161.41 (Ar-Cs); For C_13_H_14_N_6_O_3_S_2_ (366.42) Calc.: C, 42.61; H, 3.85; N, 22.94. Found: C, 42.83; H, 3.97; N, 23.17; HRMS (ESI) for C_13_H_15_N_6_O_3_S_2_: calcd, 367.0641; found, 367.0643 [M + H]^+^. HPLC: rt 11.23 min (purity 98.45%).

### 4.2 Biological Evaluation

#### 4.2.1 Determination of the COX-1 and COX-2 Inhibitory Activities

The efficacy of the synthesized sulfonamide derivatives **5a–g** and **10a–e** to inhibit COX-1 and COX-2 was assessed in vitro following previously established protocols.^27,28^

#### 4.2.2 Carbonic Anhydrase (CA) Inhibition Assay

An Applied Photophysics stopped-flow instrument was used to evaluate the ability of the synthesized sulfonamide derivatives **5a–g** and **10a–e** to inhibit CA-catalyzed CO_2_ hydration.^23^ Phenol red (0.2 mM) was used as a pH indicator and monitored at its absorbance maximum (557 nm). Measurements were performed in 20 mM HEPES buffer (pH 7.4) containing 20 mM Na_2_SO_4_ to maintain a constant ionic strength. The initial rates of the CA-catalyzed CO_2_ hydration reaction were followed for a period of 10–100 s. CO_2_ concentrations ranged from 1.7 to 17 mM for determining the kinetic parameters and inhibition constants.^28^ Enzyme concentrations ranged between 5 and 12 nM. For each inhibitor, at least six traces of the initial 5–10% of the reaction were carried out to determine the initial velocity. Uncatalyzed rates were assessed similarly and subtracted from the total observed rates. Stock solutions of the inhibitors (0.1 mM) were prepared in distilled–deionized water, and serial dilutions were subsequently performed in assay buffer to obtain final concentrations ranging down to 0.01 nM. Inhibitor and enzyme solutions were preincubated together for 15 min at room temperature to allow for the formation of the enzyme–inhibitor complex. Inhibition constants (*K*_i_) were determined by nonlinear least-squares fitting using GraphPad Prism 9 (San Diego, CA, USA) and the Cheng–Prusoff equation^29^ and represent the mean from at least three different determinations. Human *h*CA I and *h*CA II were purchased from Sigma-Aldrich, whereas recombinant *h*CA IX was produced in-house as previously described.^30^

#### 4.2.3 In Vivo Studies

All experimental procedures were conducted in accordance with the National Institutes of Health (NIH) Guidelines for the Care and Use of Laboratory Animals and in strict compliance with the ARRIVE (Animal Research: Reporting In Vivo Experiments) guidelines. Ethical approval for the study was obtained from the Research Ethics Committee of the Faculty of Pharmacy, Cairo University, Egypt (REC-FOPCU; Approval No. PC3428) Study, design, and treatment are illustrated in the Supporting Information (S84–S94).

The experimental procedures for the hot plate test to assess analgesic activity,^31^ the paw edema assay to evaluate anti-inflammatory activity,^32^ the acute ulcerogenic assay,^33^ and IOP reduction in a rabbit model of induced glaucoma^34^ were conducted in accordance with previously reported methodologies. All animals were randomly allocated to the respective experimental groups prior to treatment administration, and all pharmacological evaluations, observations, and data analyses were conducted in a blinded manner to reduce the potential experimental bias and ensure the reliability of the obtained results.

### 4.3 Crystallization and X-ray Data Collection

Crystals of *h*CAII were obtained using the hanging drop vapor diffusion method using a 24-well Linbro plate. 2 μL of a 10 mg/mL solution of *h*CA II in Tris–HCl 20 mM pH 8.0 was mixed with 2 μL of a solution of 1.5 M sodium citrate and 0.1 M Tris pH 8.0 and equilibrated against the same solution at 296 K. The complex was prepared by soaking *h*CA II native crystals in the mother liquor solution containing the inhibitors at a concentration of 10 mM for 2 days. A crystal was flash-frozen at 100 K using a solution obtained by adding 15% (v/v) glycerol to the mother liquor solution as a cryoprotectant. Data on the **10d**–CAII crystal was collected using synchrotron radiation at the ID30B beamline at ESRF (Grenoble, France) with a wavelength of 0.87313 Å and a EIGER2 X 9M detector. Data was integrated and scaled using the program XDS.^35^ Data processing statistics are shown in the Supporting Information (S65–S67).

#### 4.3.1 Structure Determination

The crystal structure of *h*CA II (PDB accession code: 9T4S) without solvent molecules and other heteroatoms was used to obtain initial phases using Refmac5.^36^ 5% of the unique reflections were selected randomly and excluded from the refinement data set for the purpose of Rfree calculations. The initial |*F*_o_ – *F*_c_| difference electron density maps unambiguously showed the inhibitor molecules. The inhibitor was introduced in the model with 1.0 occupancy. Refinements proceeded using normal protocols of positional and isotropic atomic displacement parameters alternating with manual building of the models using COOT.^37^ The quality of the final models was assessed with COOT and RAMPAGE.^38^ Crystal parameters and refinement data are summarized in the Supporting Information (S95–S102). Atomic coordinates were deposited in the Protein Data Bank (PDB accession code: 9T4S). Graphical representations were generated with Chimera.^39^

### 4.4 Molecular Modeling

#### 4.4.1 Protein and Ligand Preparation

The crystal structures of the *h*CA II and COX-2 were retrieved from the Protein Data Bank (PDB) and prepared using the protein preparation wizard module in the Schrödinger Suite (v2025). *h*CA II in complex with furosemide (PDB ID: 1Z9Y) and COX-2 in complex with rofecoxib (PDB ID: 5KIR) were selected to evaluate the binding modes of **5b** and **5d** within the binding sites of both proteins. The procedures of protein preparation involved adding hydrogen atoms, missing side chains, and missing loops while removing water molecules and ions (except zinc in the case of *h*CA II) from the protein structures. The protonation states and tautomeric forms of amino acids were optimized using the PROPKA tool at pH 7.0.^40^ Subsequently, a restrained energy minimization of the prepared protein structures was performed using the OPLS4 force field.^41^ The structures of the cognate inhibitors of *h*CA II and COX-2, along with acetazolamide, celecoxib, and the two test compounds (**5b** and **5d**), were prepared using the LigPrep module in the Schrödinger Suite (v2025) and energy-minimized using the OPLS4 force field. Possible ionization states were generated at a physiological pH of 7.0 ± 1.0 using the Epik module.^40^

#### 4.4.2 Molecular Docking

Molecular docking into the binding sites of *h*CA II and COX-2 was carried out using the Standard Precision (SP) mode of the Glide ligand docking module. Receptor grid generation was performed using the cocrystallized ligands—furosemide for *h*CAII and rofecoxib for COX-2—as the centroids, with an inner grid box size of 10 × 10 × 10 Å. To assess the ability of the docking protocol to reproduce the experimentally observed binding mode, redocking of the cocrystallized inhibitors was conducted. The root-mean-square deviation (RMSD) values of the top-scoring poses, relative to the crystallographic conformations, were found to be below 1.0 Å, indicating a high level of accuracy in pose prediction. For each compound, a maximum of 10 docking poses were generated, with all other parameters maintained at their default settings. The top-scoring Glide poses in complex with their respective proteins were subsequently subjected to 200 ns molecular dynamics (MD) simulations to evaluate the stability of their binding modes.

#### 4.4.3 Molecular Dynamics Simulations

The stability of the binding modes obtained from molecular docking was assessed by performing MD simulations for 200 ns. The Desmond simulation package was employed to set up the systems and run the MD simulations.^42^ The systems were solvated using the TIP3P water model in a periodic orthorhombic box with a 15 Å buffer distance from the protein surface in all directions and neutralized with either Na^+^ or Cl^–^ ions.^43^ Ions were placed 5 Å away from ligand atoms to avoid their interference in the ligand–protein interactions. For all the simulation runs, the OPLS4 force field and *NVT* and *NPT* (number of particles (*N*), volume (*V*), pressure (*P*), and temperature (*T*)) ensembles were utilized. Prior to performing the production simulation, the default Desmond protocol for energy minimization and model relaxation was applied. Initially, solvent molecules and ions were energy-minimized while restraining the protein–ligand complex, followed by minimization of the entire system. The system was then equilibrated in multiple stages for 12 ps per each stage: an initial *NVT* equilibration at 10 K with small restraints, followed by *NPT* equilibration with gradual restraint removal. A final unrestrained *NPT* equilibration ensured system stability before initiating the simulation production run. A cutoff of 9 Å was used to smoothly truncate the Lennard-Jones interactions and short-range Coulombic interactions. The Particle-Mesh Ewald (PME) summation was used to calculate the long-range electrostatic interactions.^44^ Finally, 200 ns MD simulations with a trajectory interval of 100 ps were carried out at a temperature of 300 K and a pressure of 1.01325 bar in the *NPT* ensemble using a Nose–Hoover chain thermostat and a Martyna–Tobias–Klein barostat.^45,46^ The trajectories were saved at 2 fs intervals for further analysis using the Simulation Interaction Diagram (SID) tool implemented in the Desmond MD package. SID was used to analyze the interactions between the compound and the protein. The stability of the complexes was monitored by examining the protein Cα and ligand RMSD over time using frame zero as a reference.

## Supplementary Material













## Notes

Spectral data (NMR, HRMS, and HPLC purity spectra); in vitro and in vivo biological assays data; X-ray crystallography; and molecular docking (PDF)
Molecular formula strings of compounds celecoxib, **SLC-558**, **II**, **III**, **IV**, **V**, **VI**, **5a–g**, **10a–e**, **AAZ**, and **ibuprofen** (CSV)
Co-crystallized ligand (**10d**) in CA II (PDB: 9T4S) (PDB)
Docking pose for compound **5b** and **5d** within COX-2 (PDB ID: 5KIR) and CAII (PDB ID: 1Z9Y) (PDB)
Docking pose for compound **5b** within COX-2 (PDB ID: 5KIR) (PDB)
Docking pose for compound **5d** within COX-2 (PDB ID: 5KIR) (PDB)

## Abbreviations

Å: angstrom
AAZ: acetazolamide
CA: carbonic anhydrase
CAI: CA inhibitor
COX: cyclooxygenase
DMSO: dimethyl sulfoxide
IOP: intraocular pressure
RMSD: root-mean-square deviation
SI: selectivity index
ZBGs: zinc binding groups
