# Supplementary material for: Hybrids of Benzenesulfonamide Oxadiazole Derivatives with Dual CA II and COX‑2 Inhibitory Activity Demonstrating Antiglaucoma and Anti-inflammatory Action: Synthesis, In Silico Insights, and In Vitro and In Vivo Bioevaluation
Source: J Med Chem. 2026 Jul 2;69(14):17243–59. doi: 10.1021/acs.jmedchem.6c01117 (PMC13403231; doi:10.1021/acs.jmedchem.6c01117)
Supplement: Supplementary file 1 [file jm6c01117_si_001.pdf]

Supporting Information (SI)

Hybrids of benzenesulfonamide oxadiazole derivatives with dual CA II and COX-2 inhibitory activity demonstrating anti-glaucoma and anti-inflammatory action: Synthesis, *in silico* insights, *in vitro* and *in vivo* bio-evaluation.

Manal Abdel Fattah Ezzat [1,§,*], Emad M. Seif [2,§], Husam Nassar,[3] Alessandro Bonardi[4], Marta Ferraroni[5], Ahmed A. Attia [6], Yomna T. T. Khater [7], Rabab Ahmed Rasheed [8], Omnia AM Abd El-Ghafar [9], Heba Abdelrasheed Allam [1], Andrea Angeli[10], Matthias Schmidt[3], Claudiu T. Supuran [4,*], Hany S. Ibrahim [3,11,*]

[1]*Department of Pharmaceutical Chemistry, Faculty of Pharmacy, Cairo University, Cairo 11562, Egypt*
[2]*Department of Pharmaceutical Chemistry, Faculty of Pharmacy, October University for Modern Sciences and Arts University (MSA), Giza 12451, Egypt*
[3]*Department of Medicinal Chemistry, Institute of Pharmacy, Martin-Luther-University of Halle-Wittenberg, 06120 Halle (Saale), Germany*
[4]*Department NEUROFARBA─Pharmaceutical and Nutraceutical Section, University of Firenze, via Ugo Schiff 6, Sesto Fiorentino I-50019, Firenze, Italy*
[5]*Department of Chemistry "Ugo Schiff", University of Florence, Via della Lastruccia 3-13, I-50019, Sesto Fiorentino, Italy*
[6]*Mansoura Ophthalmic Centre, Faculty of Medicine, Mansoura University, Mansoura 35516, Egypt*
[7]*Medical Experimental Research Center, Faculty of Medicine, Mansoura University, Mansoura 35516, Egypt*
[8]*Department of Medical Histology and Cell Biology, School of Medicine, Badya University, Giza 12573, Egypt*
[9]*Department of Pharmacology and Toxicology, Faculty of Pharmacy, Nahda University, Beni-Suef 62764, Egypt*
[10]*NEUROFARBA Department, Sezione di Scienze Farmaceutiche, University of Florence, Via Ugo Schiff 6, 50019, Sesto Fiorentino, Florence, Italy*
[11]*Department of Pharmaceutical Chemistry, Faculty of Pharmacy, Egyptian Russian University, Badr City, Cairo 11829, Egypt*
[§] **Authors are equally contributed**

Corresponding Authors (H.S. Ibrahim) (hany.ibrahim@pharmazie.uni-halle.de); (C.T. Supuran) (claudiu.supuran@unifi.it); (M.A.F. Ezzat) (manal.salem@pharma.cu.edu.eg).

Topic	Page
**1. ^{1}H and ^{13}C NMR charts of the final compounds**	**7**
**Figure S1.** ^{1}H NMR spectrum of compound **5a**	**8**
**Figure S2.** ^{13}C NMR spectrum of compound **5a**	**9**
**Figure S3.** ^{1}H NMR spectrum of compound **5b**	**10**
**Figure S4.** ^{13}C NMR spectrum of compound **5b**	**11**
**Figure S5.** ^{1}H NMR spectrum of compound **5c**	**12**
**Figure S6.** ^{1}H NMR spectrum of compound **5c (D_2O)**	**13**
**Figure S7.** ^{13}C NMR spectrum of compound **5c**	**14**
**Figure S8.** ^{1}H NMR spectrum of compound **5d**	**15**
**Figure S9.** ^{1}H NMR spectrum of compound **5d (D_2O)**	**16**
**Figure S10.** ^{13}C NMR spectrum of compound **5d**	**17**
**Figure S11.** ^{1}H NMR spectrum of compound **5e**	**18**
**Figure S12.** ^{1}H NMR spectrum of compound 5e **(D_2O)**	**19**
**Figure S13.** ^{13}C NMR spectrum of compound **5e**	**20**
**Figure S14.** ^{1}H NMR spectrum of compound **5f**	**21**
**Figure S15.** ^{1}H NMR spectrum of compound **5f (D_2O)**	**22**
**Figure S16.** ^{13}C NMR spectrum of compound **5f**	**23**
**Figure S17.** ^{1}H NMR spectrum of compound **5g**	**24**
**Figure S18.** ^{13}C NMR spectrum of compound **5g**	**25**
**Figure S19.** ^{1}H NMR spectrum of compound **10a**	**26**
**Figure S20.** ^{13}C NMR spectrum of compound **10a**	**27**

Figure S21. ^{1}H NMR spectrum of compound **10b** **28**

Figure S22. ^{1}H NMR spectrum of compound **10b** **29**

Figure S23. ^{13}C NMR spectrum of compound **10b** **30**

Figure S24. ^{1}H NMR spectrum of compound **10c** **31**

Figure S25. ^{1}H NMR spectrum of compound **10c** **32**

Figure S26. ^{1}H NMR spectrum of compound **10d** **33**

Figure S27. ^{1}H NMR spectrum of compound **10d** **34**

Figure S28. ^{1}H NMR spectrum of compound **10d** **35**

Figure S29. ^{13}C NMR spectrum of compound **10d** **36**

Figure S30. ^{1}H NMR spectrum of compound **10e** **37**

Figure S31. ^{1}H NMR spectrum of compound **10e** **38**

Figure S32. ^{13}C NMR spectrum of compound **10e** **39**

2. HRMS SPECTRA OF THE FINAL COMPOUNDS **40**

Figure S33. HRMS spectrum of compound **5a** **431**

Figure S34. HRMS spectrum of compound **5b** **432**

Figure S35. HRMS spectrum of compound **5c** **43**

Figure S36. HRMS spectrum of compound **5d** **434**

Figure S37. HRMS spectrum of compound **5e** **45**

Figure S38. HRMS spectrum of compound **5f** **46**

Figure S39. HRMS spectrum of compound **10a** **47**

Figure S40. HRMS spectrum of compound **10b** **48**

Figure S41. HRMS spectrum of compound **10c** **49**

Figure S42. HRMS spectrum of compound **10d** **50**

Figure S43. HRMS spectrum of compound **10e** **51**

3. HPLC PURITY CHARTS **52**

Figure S44. HPLC chromatogram of compound **5a** **53**

Figure S45. HPLC chromatogram of compound **5b** **54**

Figure S46. HPLC chromatogram of compound **5c** **55**

Figure S47. HPLC chromatogram of compound **5d** **56**

Figure S48. HPLC chromatogram of compound **5e** **57**

Figure S49. HPLC chromatogram of compound **5f** **58**

Figure S50. HPLC chromatogram of compound **5g** **59**

Figure S51. HPLC chromatogram of compound **10a** **60**

Figure S52. HPLC chromatogram of compound **10b** **61**

Figure S53. HPLC chromatogram of compound **10c** **62**

Figure S54. HPLC chromatogram of compound **10d** **63**

Figure S55. HPLC chromatogram of compound **10e** **64**

4. X-RAY CRYSTALLOGRAPHIC ANALYSIS **65**

Figure S56. Electron density of inhibitor 10d (green, PDB: 9T4S) bound to zinc (grey) in hCA II active site. 2Fo-Fc maps and contoured to the 1.0 σ level. Error! Bookmark not defined.

Table S1. Summary of Data Collection and Atomic Model Refinement Statistics for hCAII Error! Bookmark not defined.

5. BIOLOGICAL EVALUATION **68**

Figure S57. *In vitro* COX-1 inhibitory Activity of target compounds and celecoxib standard **79**

Figure S58. *In vitro* COX-2 inhibitory Activity of target compounds and celecoxib standard **82**

Figure S59. Inhibition plots of the most representative compounds 5b and 5d against hCA I, II, and XII. **83**

Table S2. Detailed statistical analysis for the analgesic study. **84**

Table S3. Detailed statistical analysis for carrageenan-induced Paw Edema study. **87**

Figure S60. Ulcerogenic effect of A): control group; B): Ibuprofen group; C): Compound **5b** group; D): Compound **5d** group in rats after 24 hrs of their administration. **89**

Table S4. Detailed statistical analysis for intraocular pressure assay. **91**

Table S5. Baseline body weights and randomization allocation scheme for rabbits in the exploratory IOP assay (*n* =4 per group). **92**

Table S6. Individual raw IOP measurements for rabbits in group A over time. **92**

Table S7. Mean and standard deviation (SD) of IOP measurements over time for rabbits in group A (*n*=4). **93**

Table S8. Individual raw IOP measurements for rabbits in group B over time. **93**

Table S9. Mean and standard deviation (SD) of IOP measurements over time for rabbits in group B (*n*=4). **94**

6. MOLECULAR MODELING 95

Figure S61. Validation of the docking protocol using furosemide (PDB 1Z9Y) and rofecoxib (PDB 5KIR) **96**

Figure S62. Docked poses of *h*CAII–furosemide and COX-2–rofecoxib **96**

Figure S63. MD simulations of docked furosemide and rofecoxib poses **96**

Figure S64. Interaction occupancy rates of furosemide and rofecoxib with amino acid residues **97**

**Figure S65.** Snapshots of furosemide and rofecoxib at 0 ns and 200 ns of the simulation trajectories	**97**
**Figure S66.** Root Mean Square Fluctuations (RMSF)	**98**
**Figure S67.** Snapshots of acetazolamide, celecoxib, 5b and 5d at 0 ns and 200 ns of the simulation trajectories	**99**
**Figure S68.** RMSF of acetazolamide, celecoxib, **5b** and **5d**	**100**
**Table S10.** *h*CAII-5b Interactions and Contact Distances within the Active Site	**100**
**Table S11.** *h*CAII-5d Interactions and Contact Distances within the Active Site	**100**
**Table S12.** COX2-5b Interactions and Contact Distances within the Active Site	**101**
**Table S13.** COX2-5d Interactions and Contact Distances within the Active Site	**101**
**Figure S69.** The spatial relationship between the phenyl ring of Phe131 in *h*CAII and the aromatic rings of (A) furosemide, (B) compound 5b and (C) compound 5d. The closet atom-centroid distance is colored pink while the centroid-centroid distance is colored blue. The angle between the planes of the rings is approximately 80°. These metrics support the formation of a T-shaped (edge to face) π- π stacking interactions.	**102**

1. ^{1}H and ^{13}C NMR charts of the final compounds

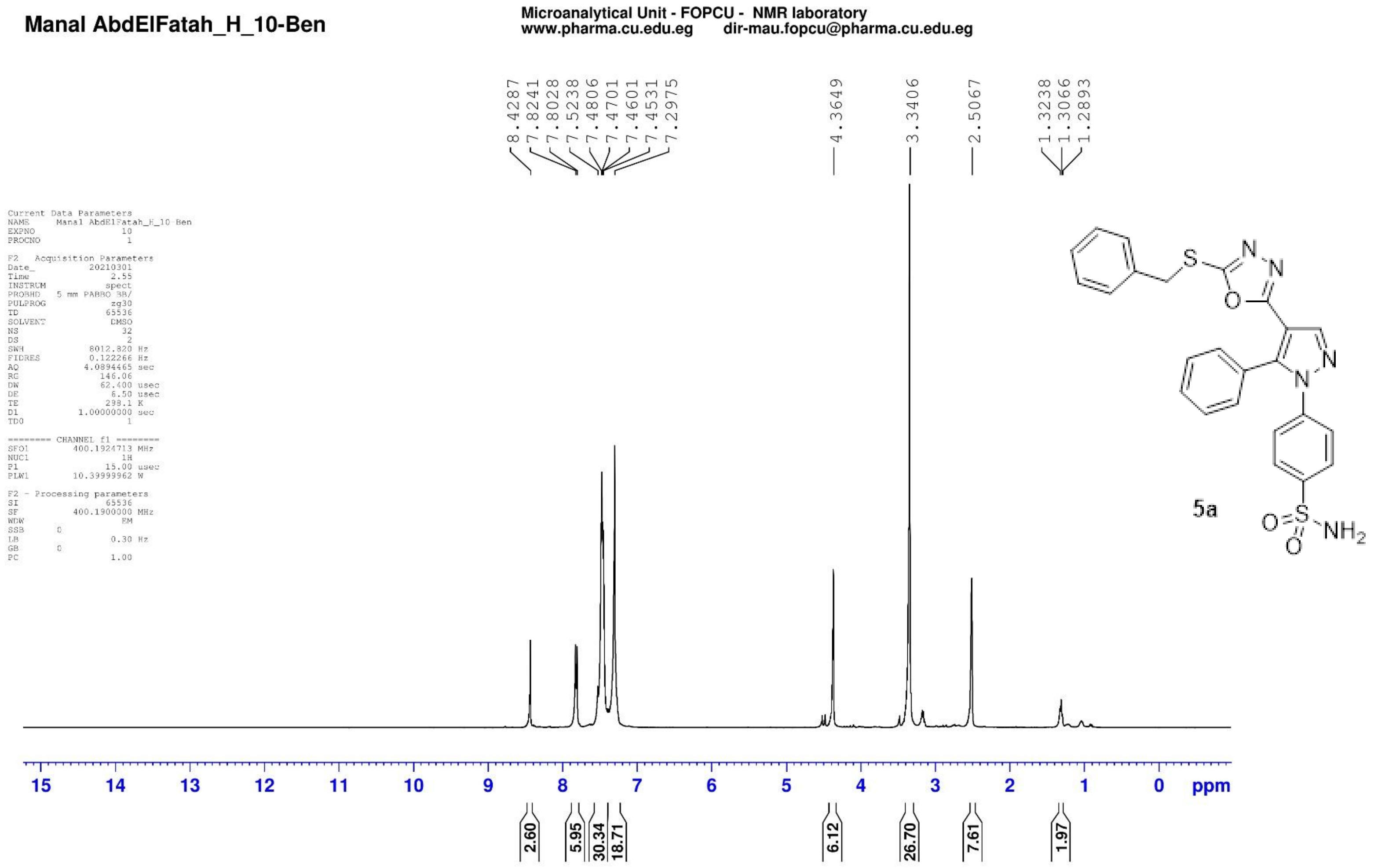

Figure S1. ^{1}H NMR spectrum of compound **5a**

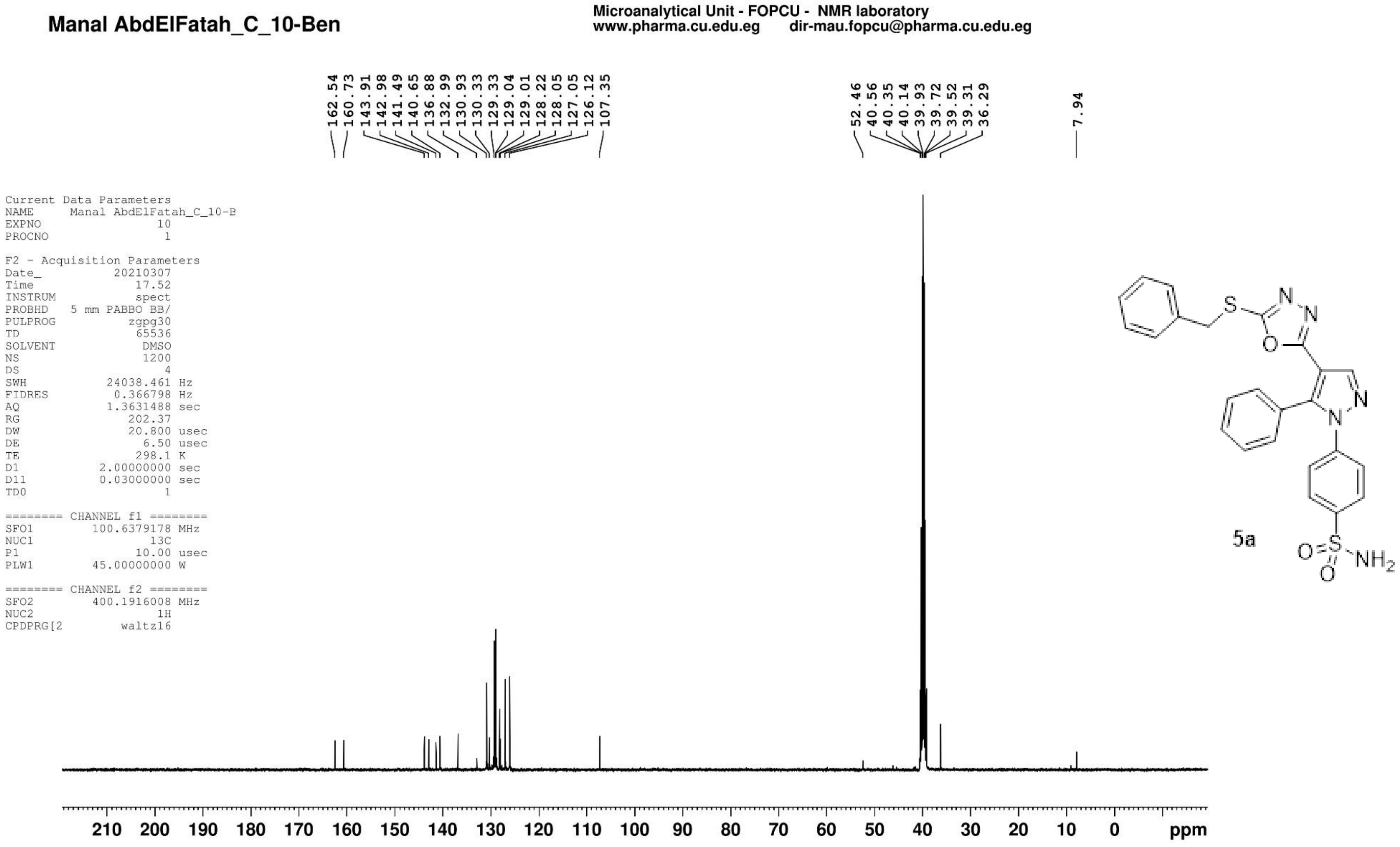

Figure S2. ^{13}C NMR spectrum of compound **5a**

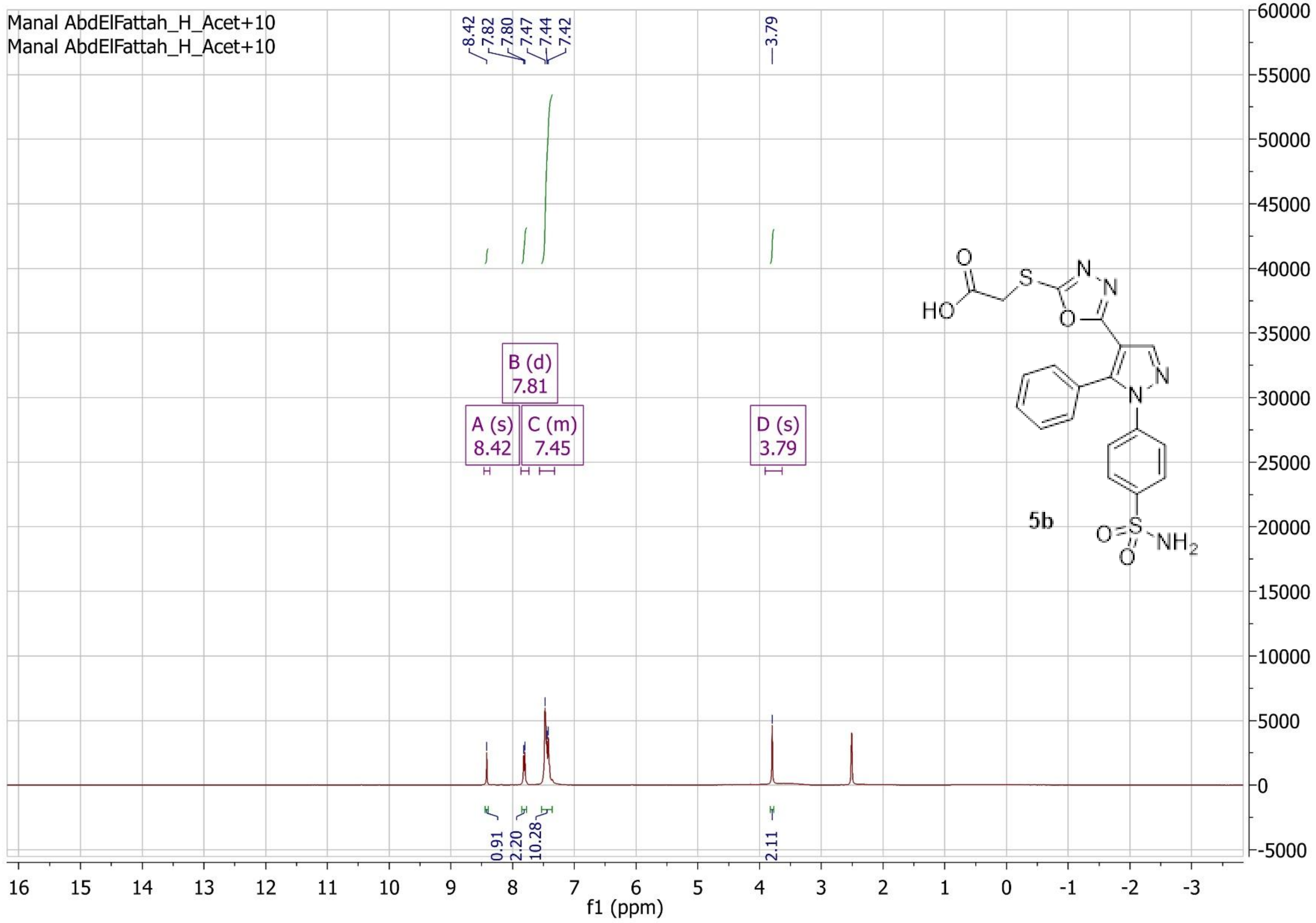

Figure S3. ^{1}H NMR spectrum of compound **5b**

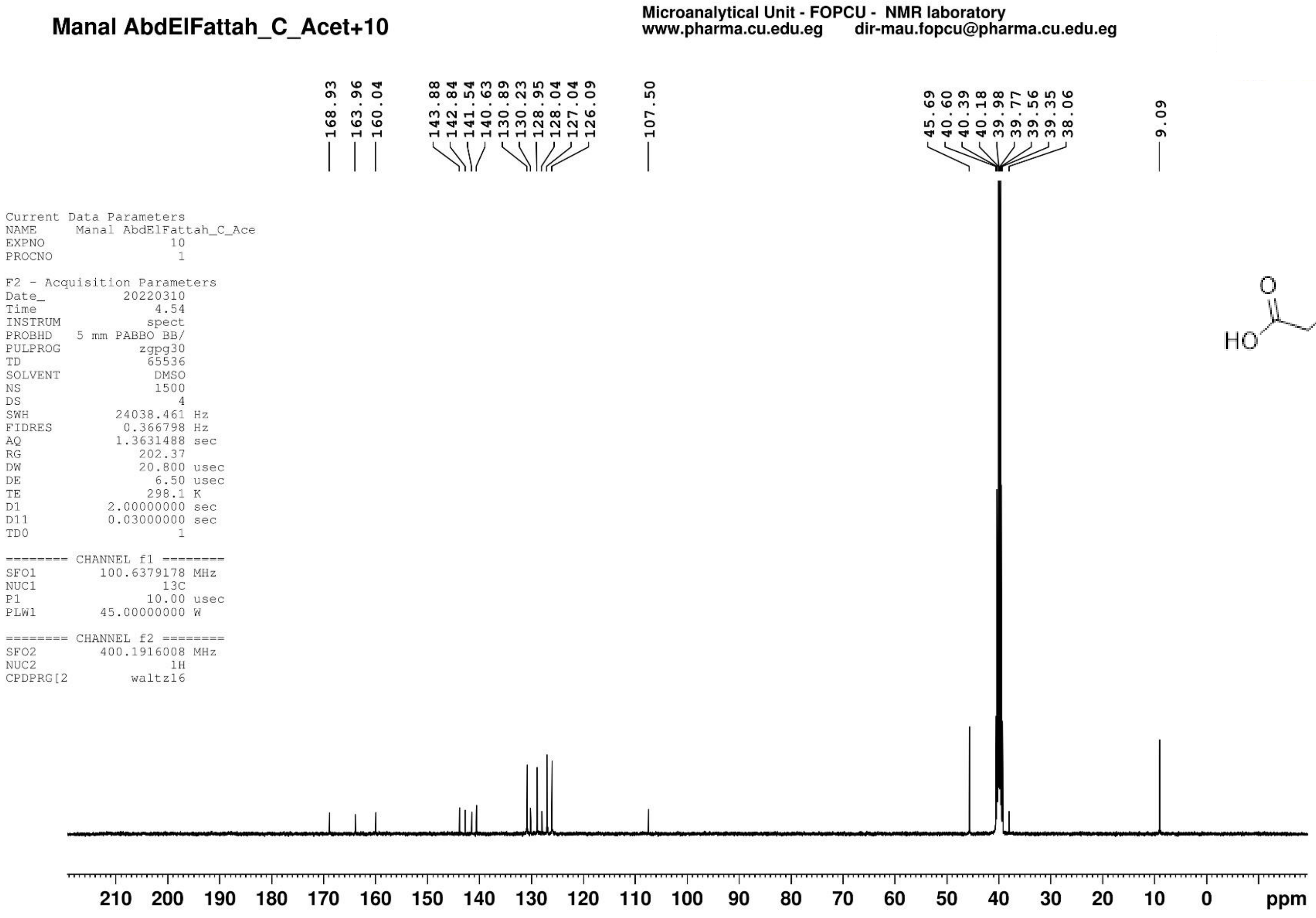

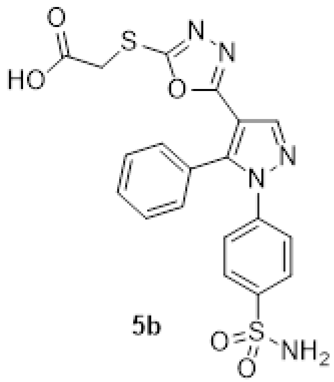

5b

Figure S4. ^{13}C NMR spectrum of compound **5b**

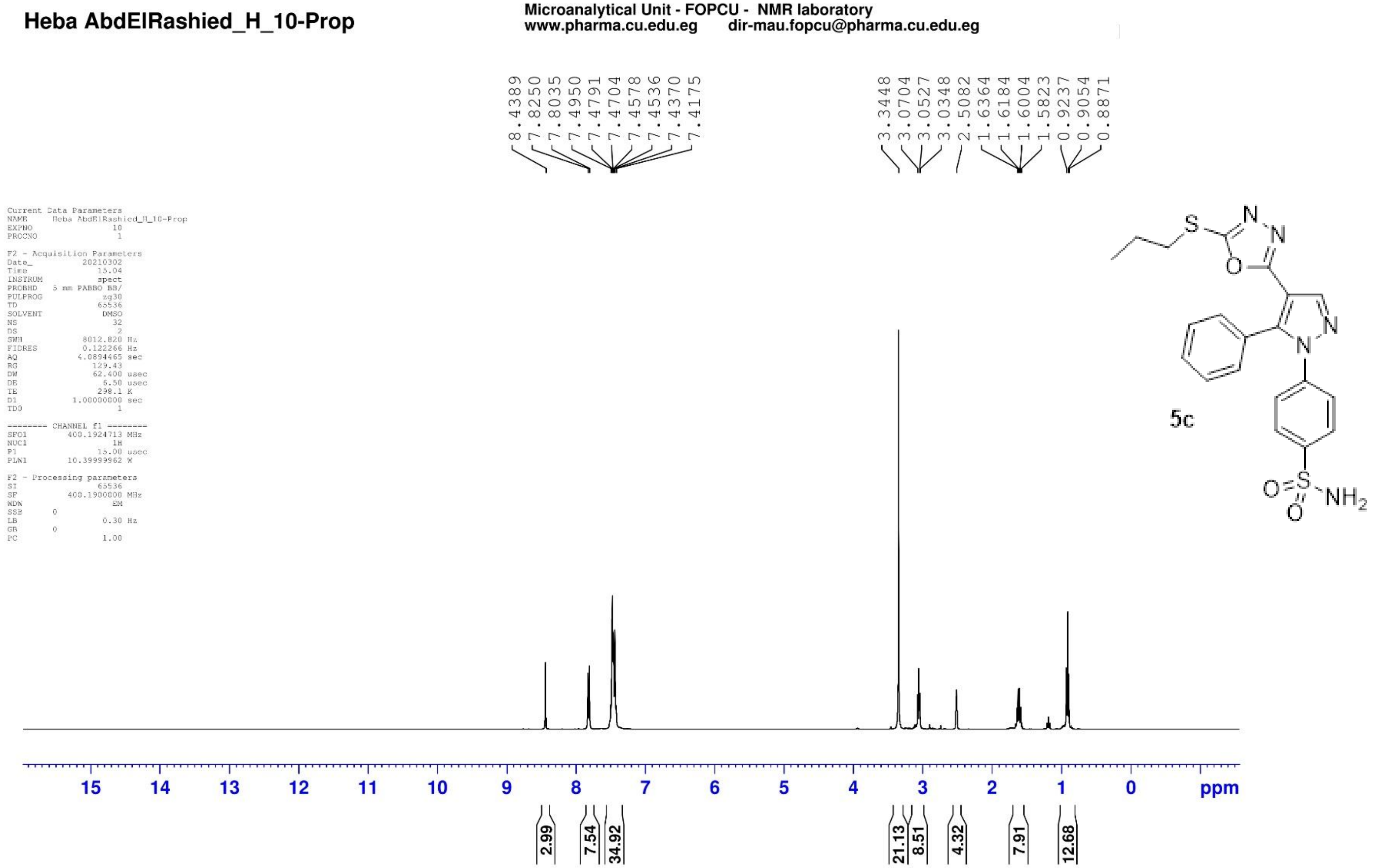

Figure S5. ^{1}H NMR spectrum of compound **5c**

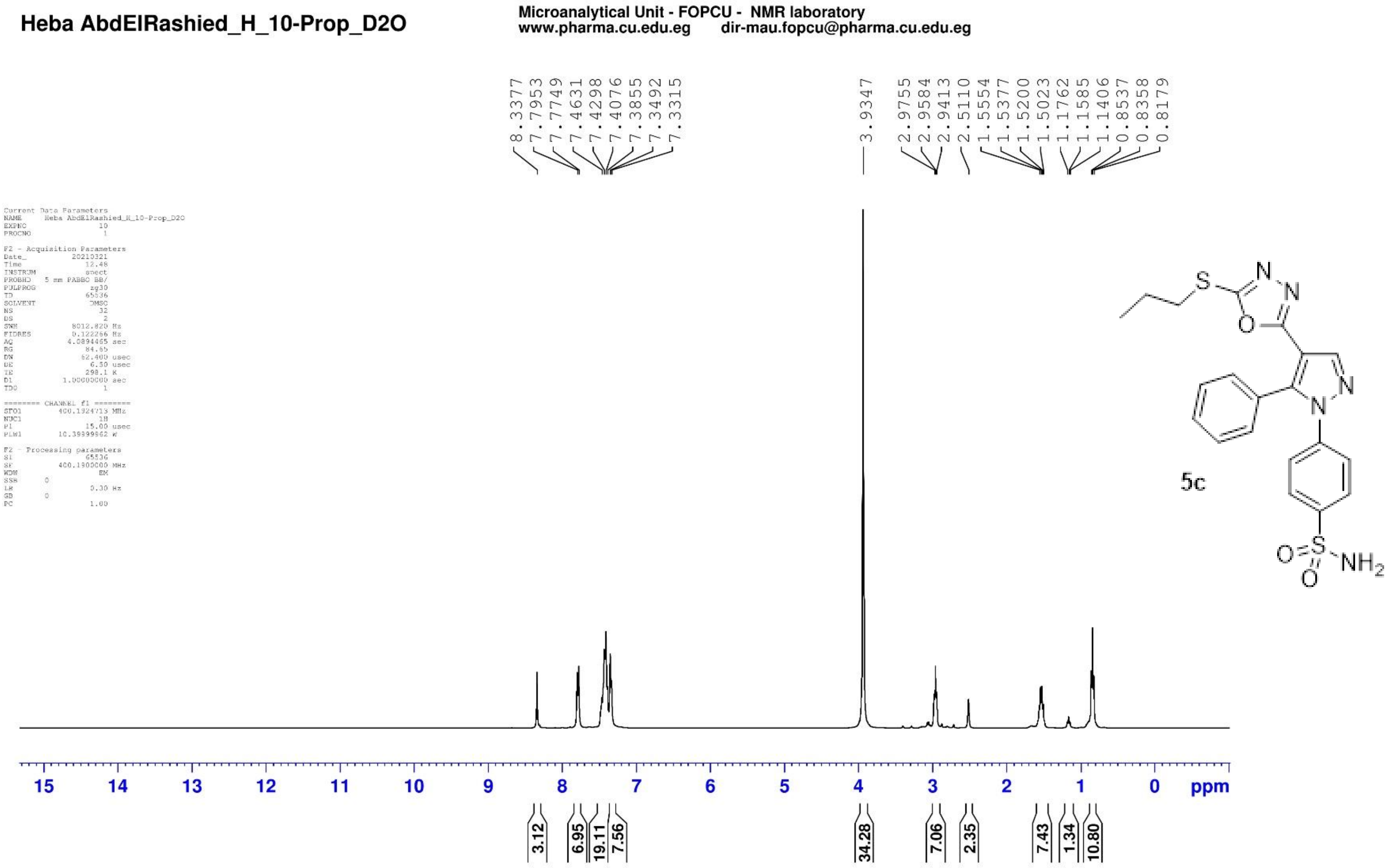

Figure S6. ^{1}H NMR spectrum of compound **5c (D_2O)**

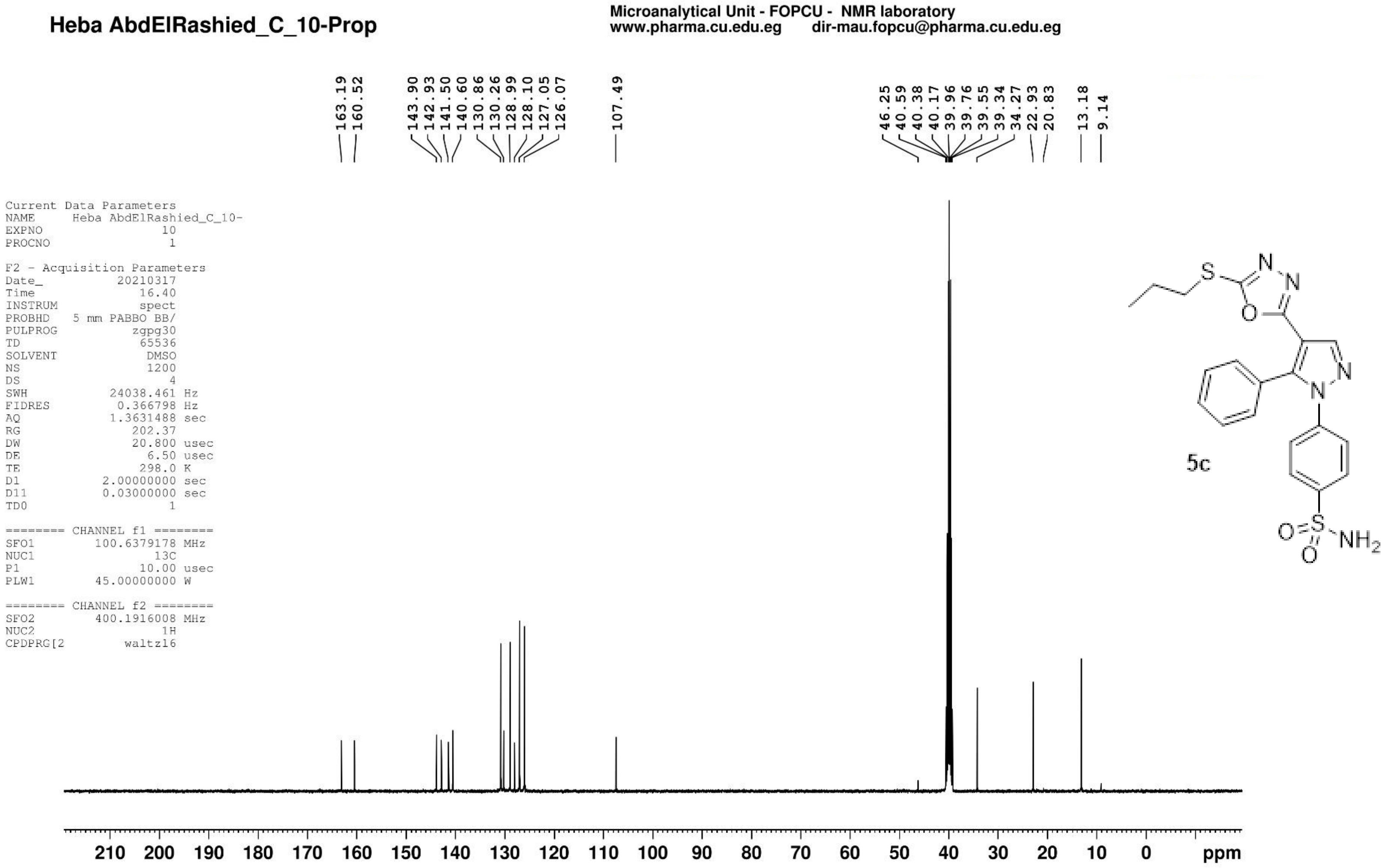

Figure S7. ^{13}C NMR spectrum of compound **5c**

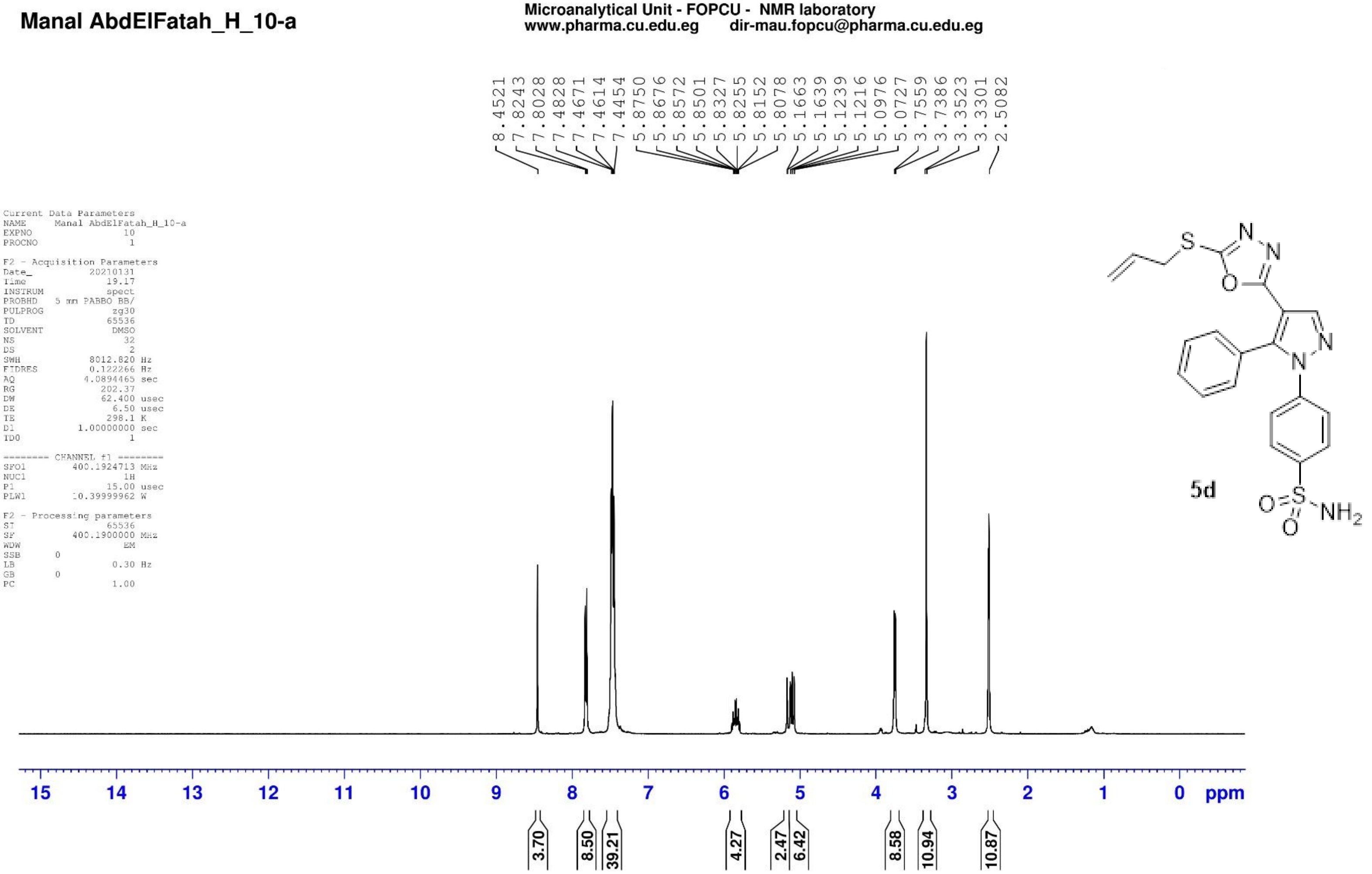

Figure S8. ^{1}H NMR spectrum of compound **5d**

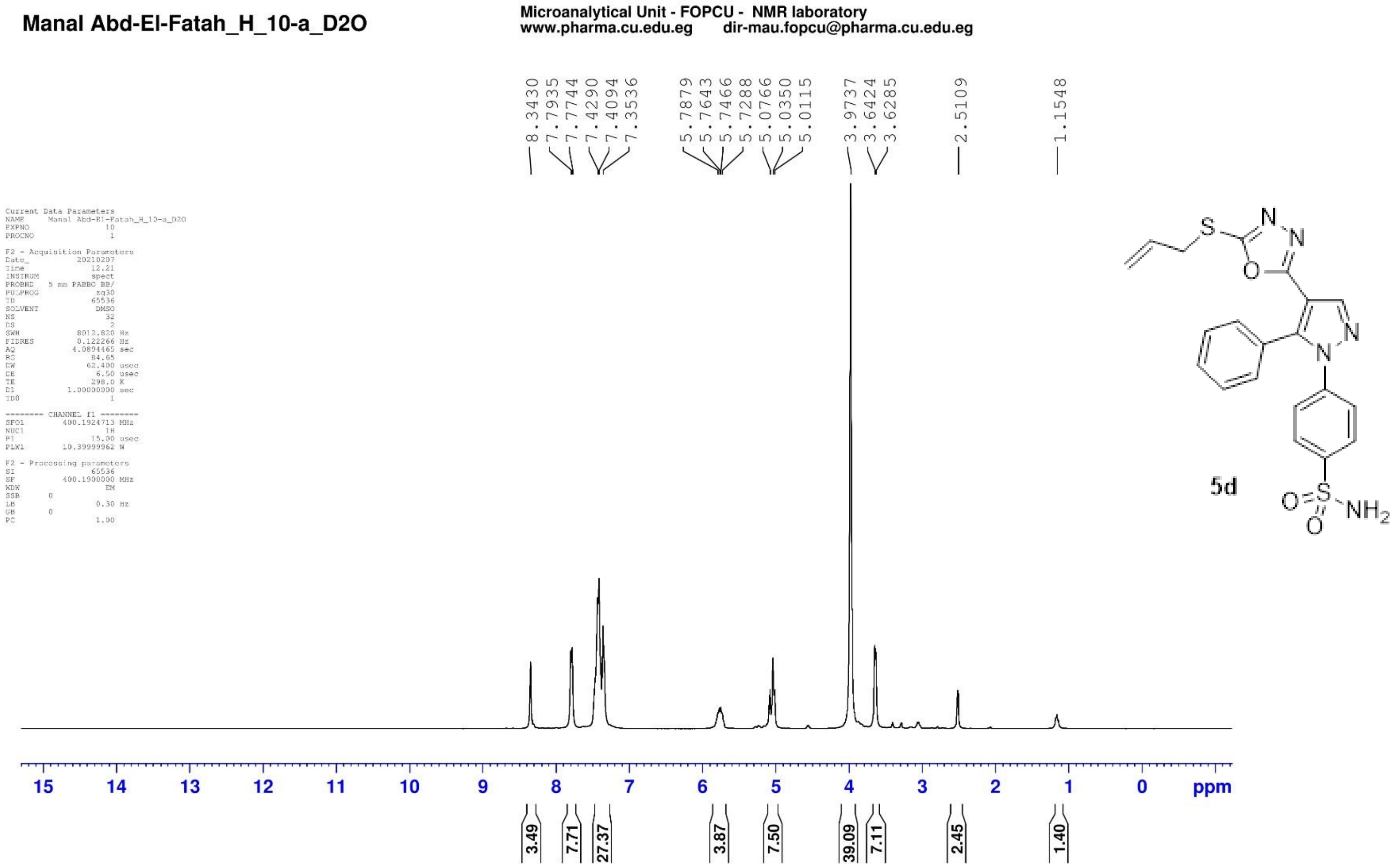

Figure S9. ^{1}H NMR spectrum of compound **5d (D_2O)**

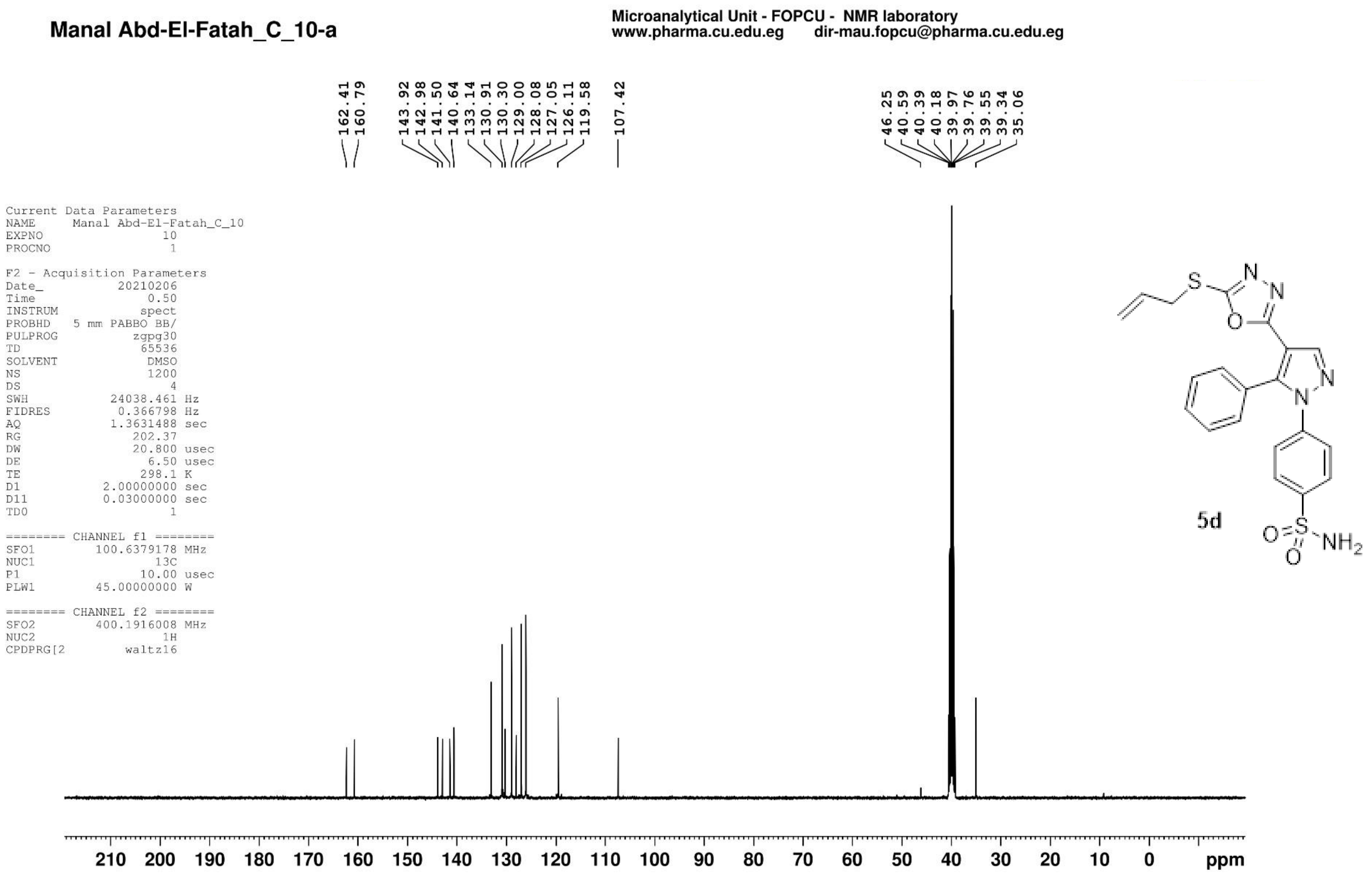

Figure S10. ^{13}C NMR spectrum of compound **5d**

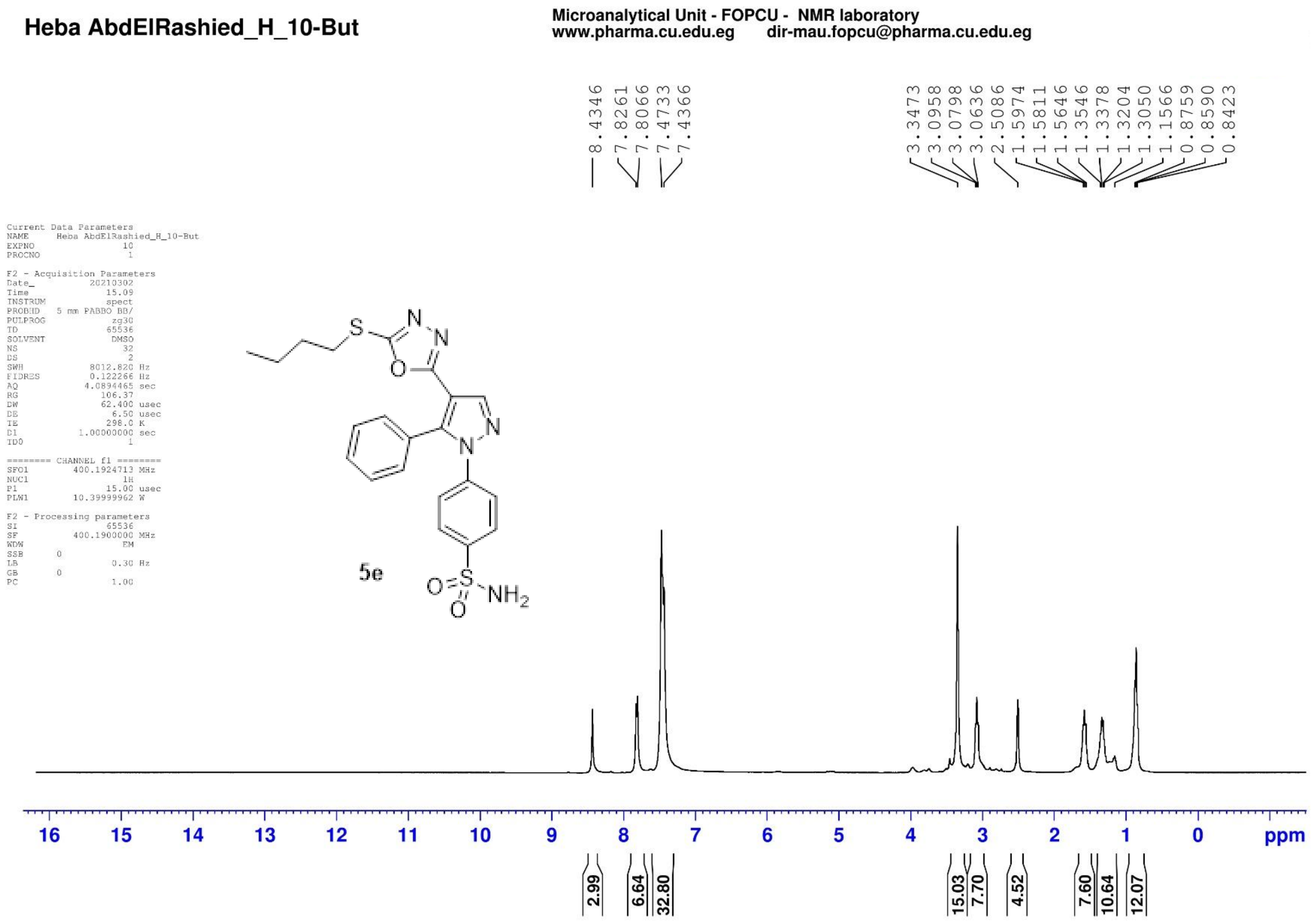

Figure S11. ^{1}H NMR spectrum of compound **5e**

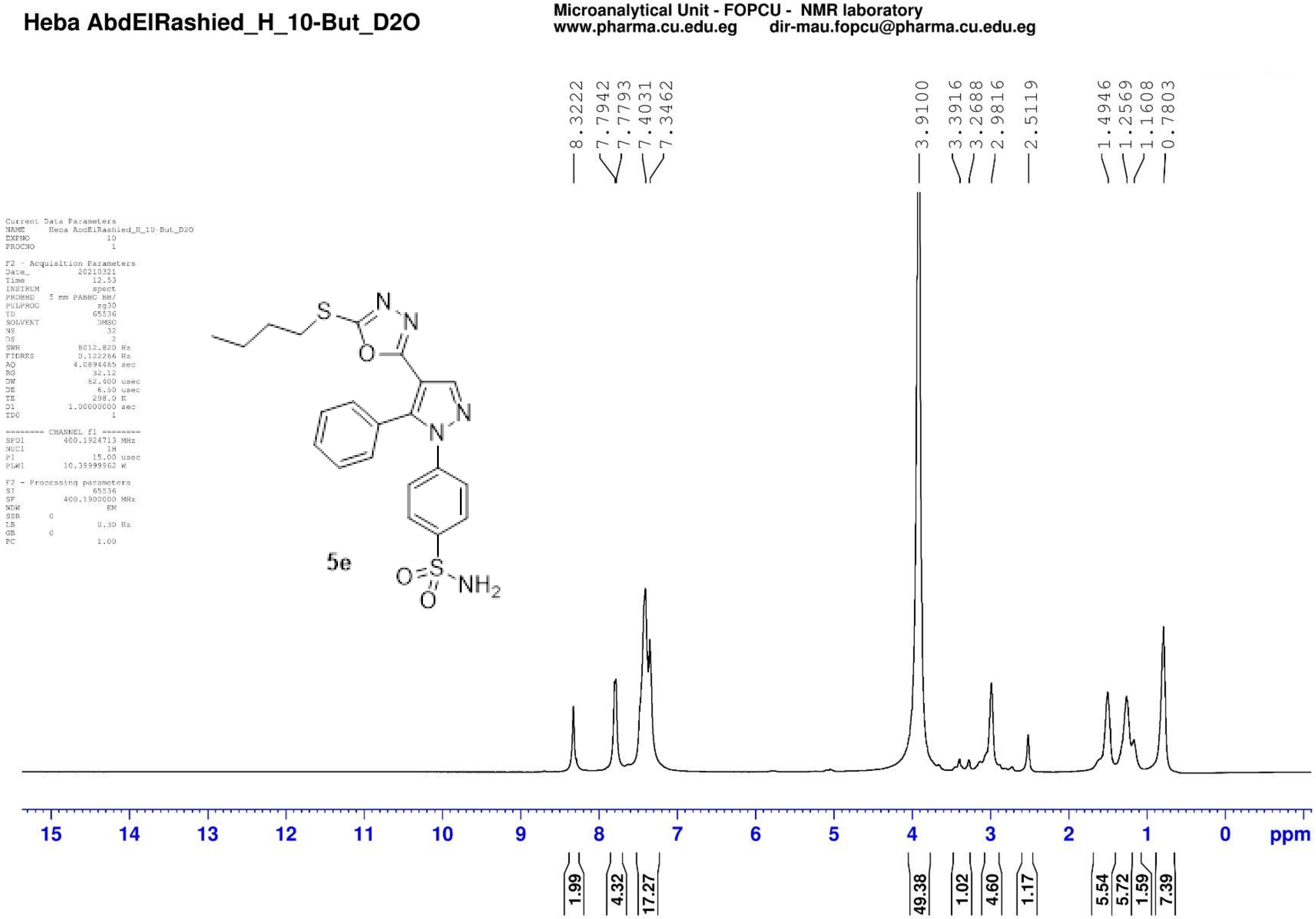

Figure S12. ^{1}H NMR spectrum of compound 5e **(D$_2$O)**

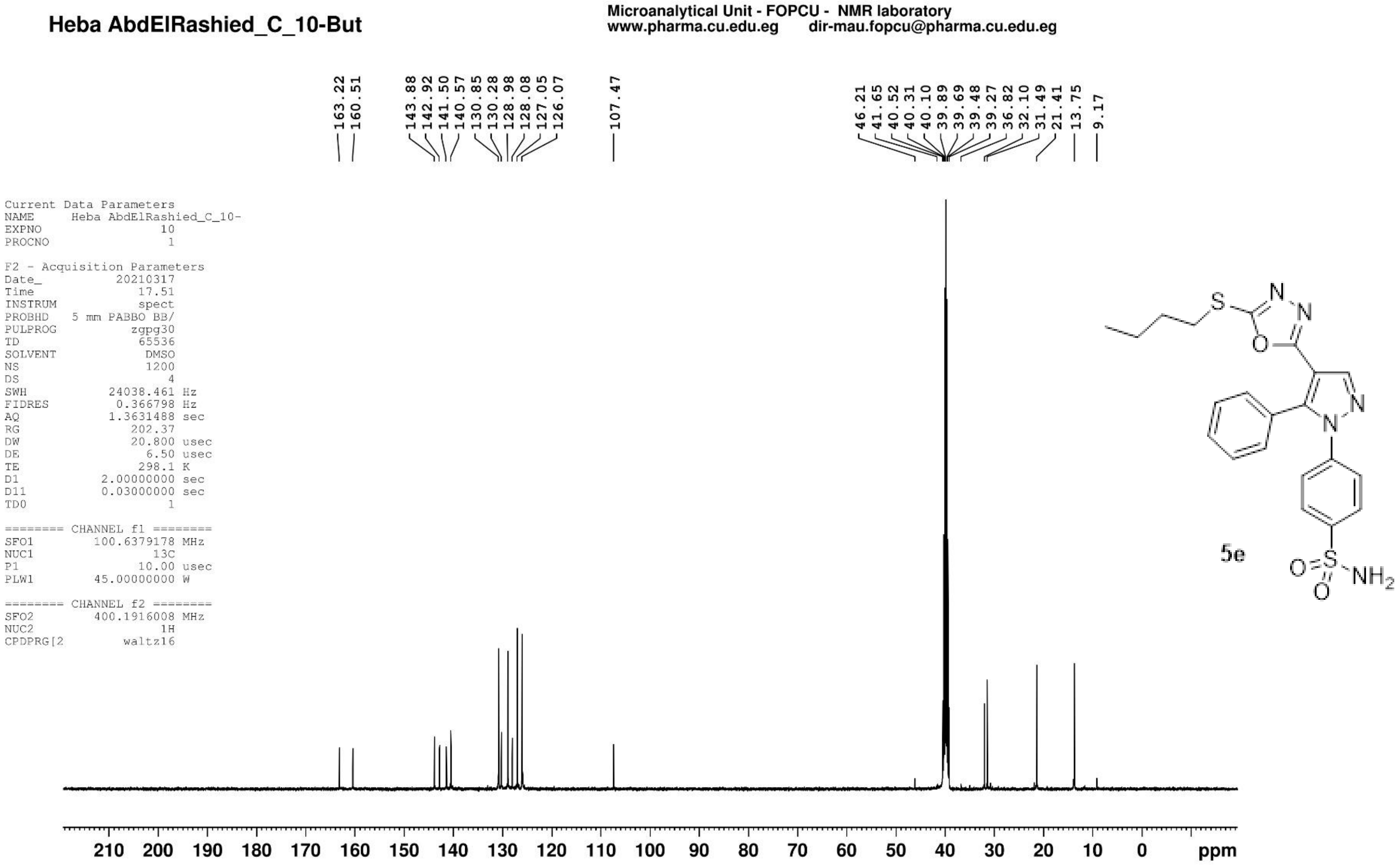

Figure S13. ^{13}C NMR spectrum of compound **5e**

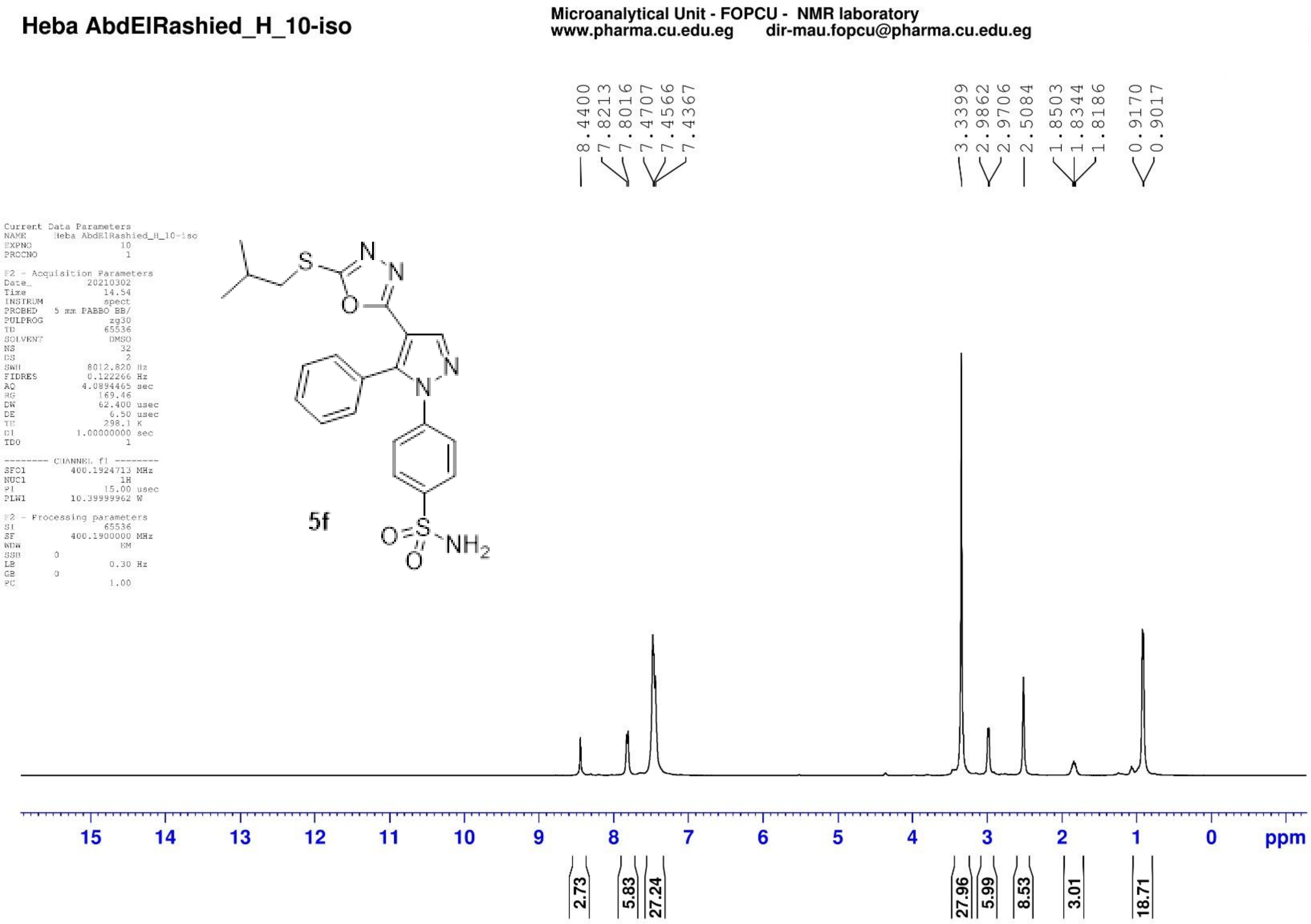

Figure S14. ^{1}H NMR spectrum of compound **5f**

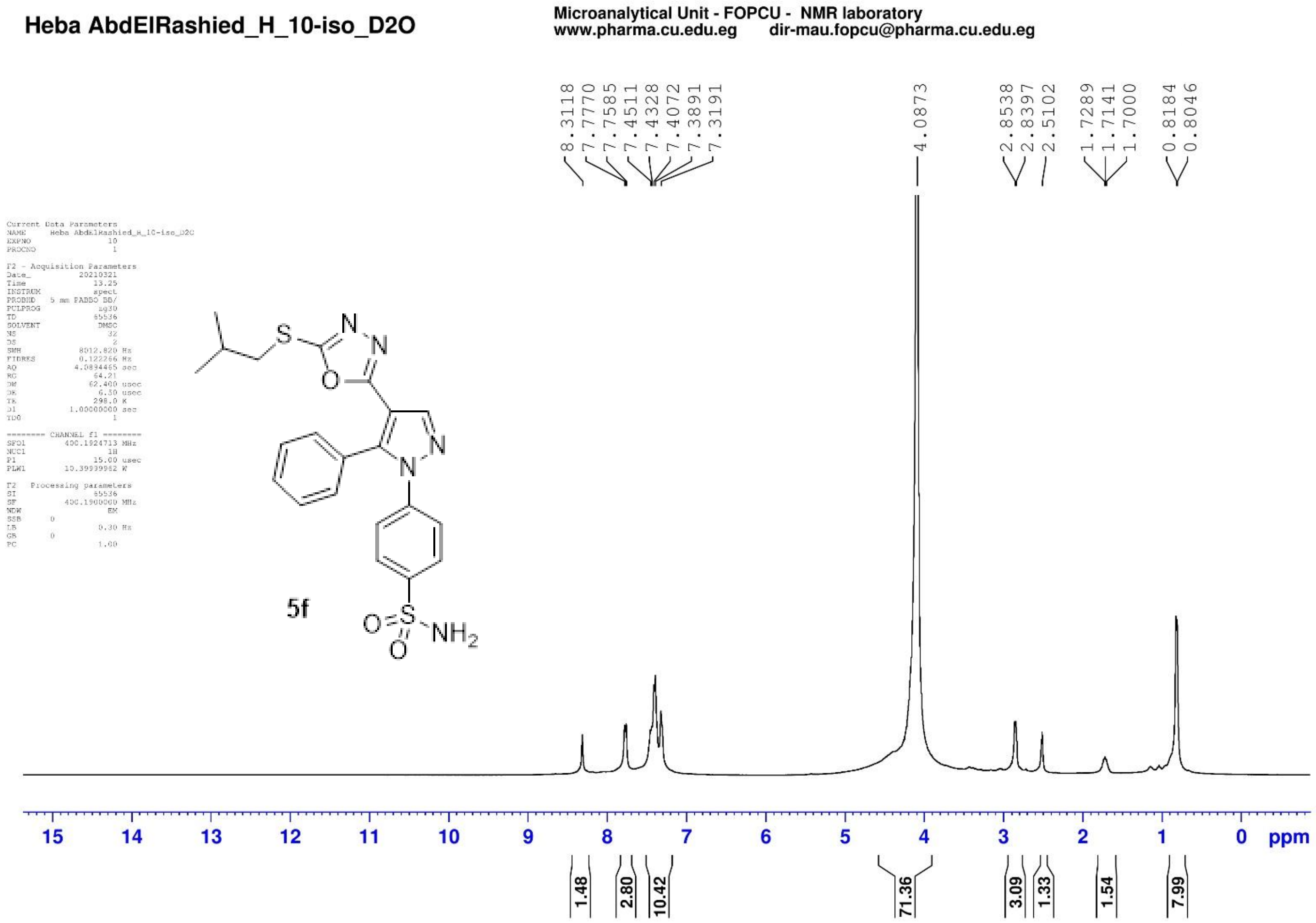

Figure S15. ^{1}H NMR spectrum of compound **5f (D_2O)**

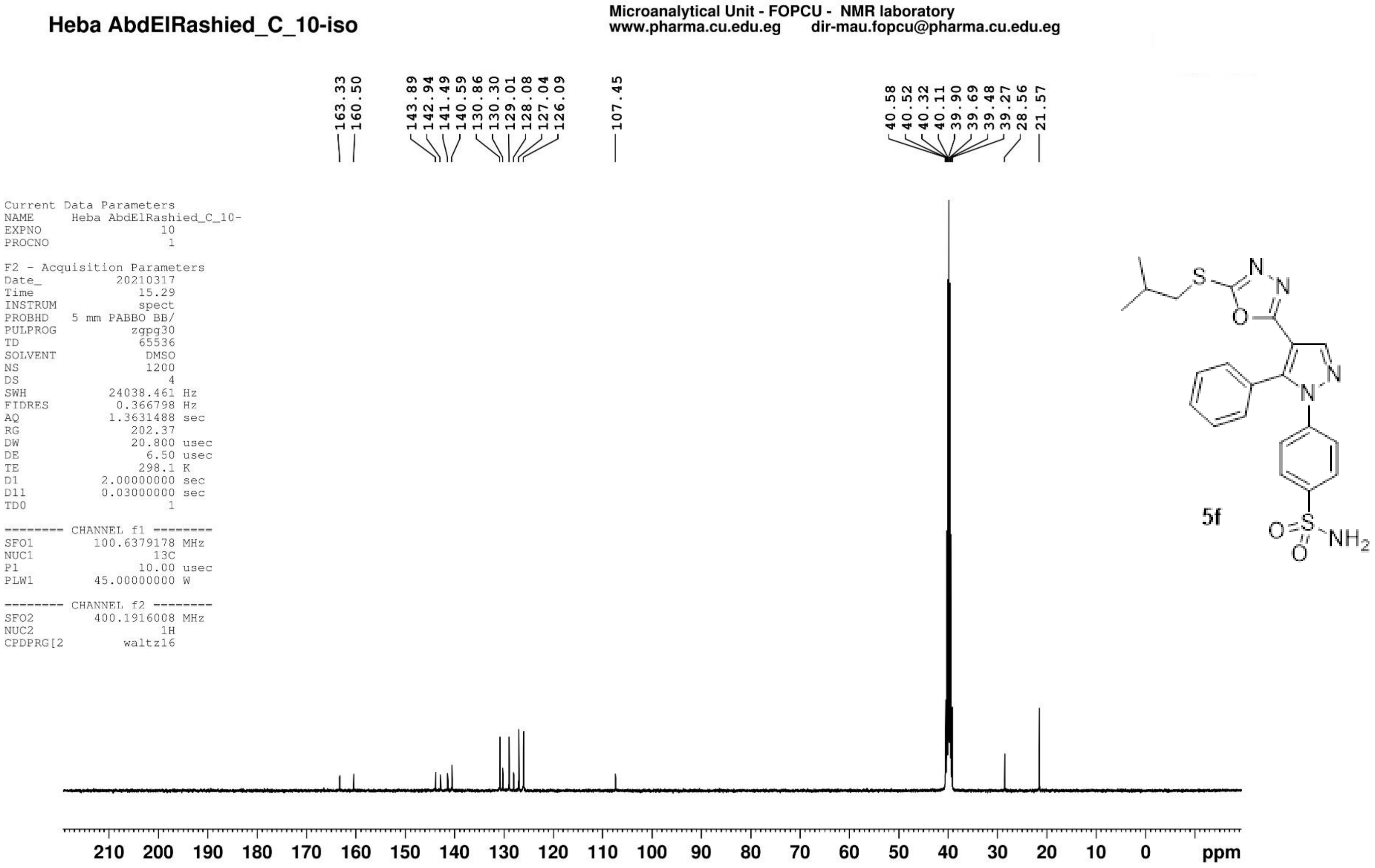

Figure S16. ^{13}C NMR spectrum of compound **5f**

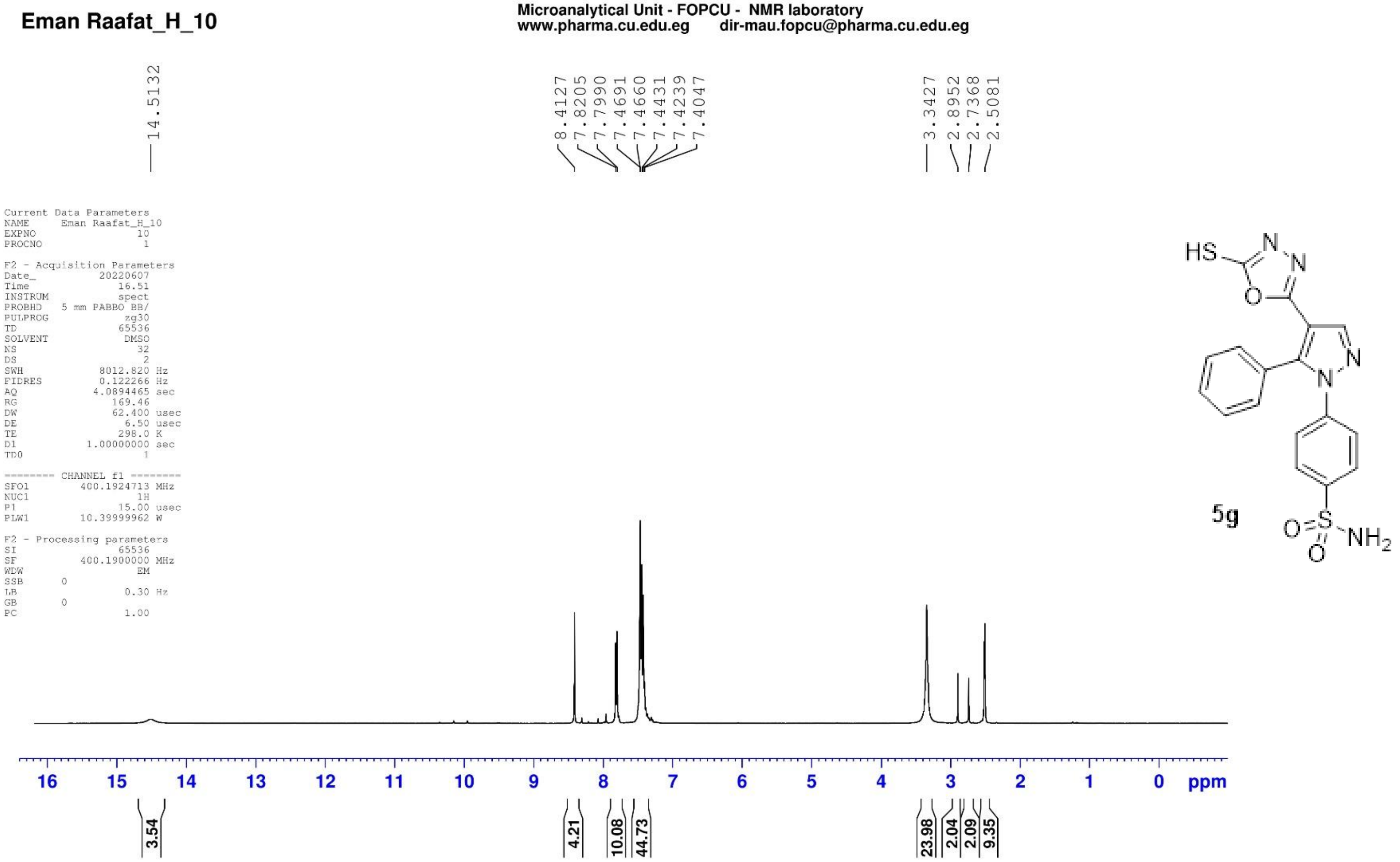

Figure S17. ^{1}H NMR spectrum of compound **5g**

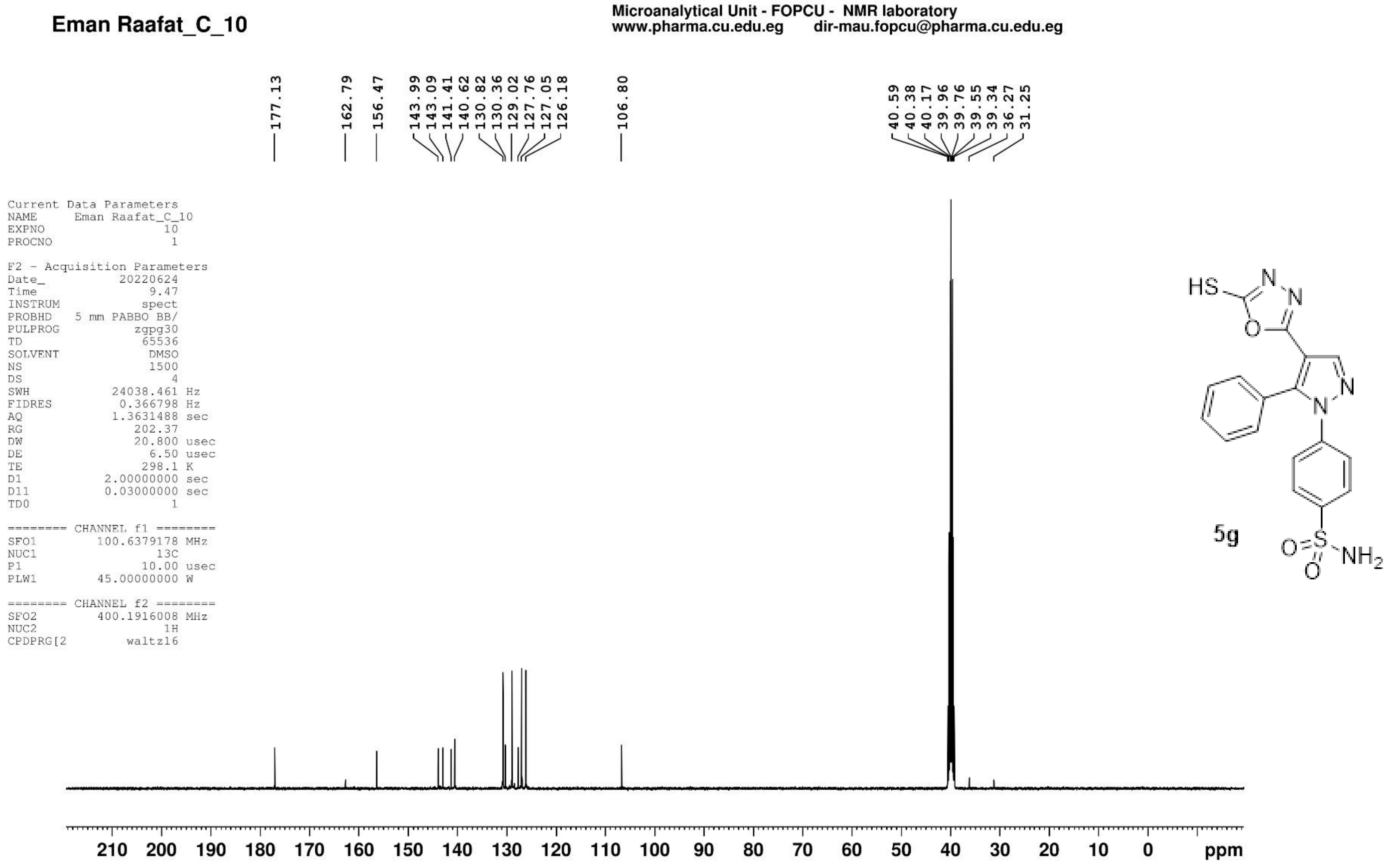

Figure S18. ^{13}C NMR spectrum of compound **5g**

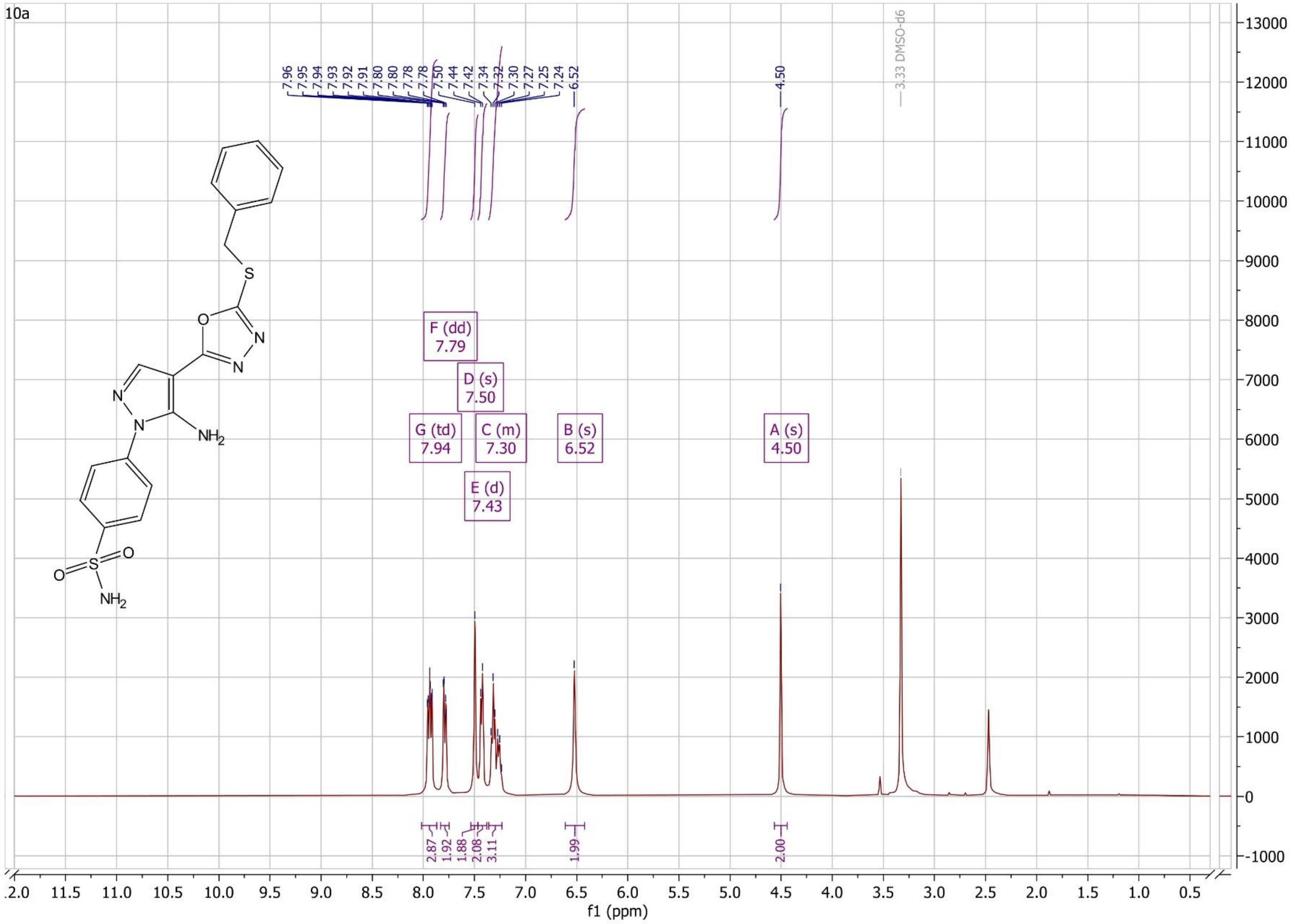

Figure S19. ^{1}H NMR spectrum of compound **10a**

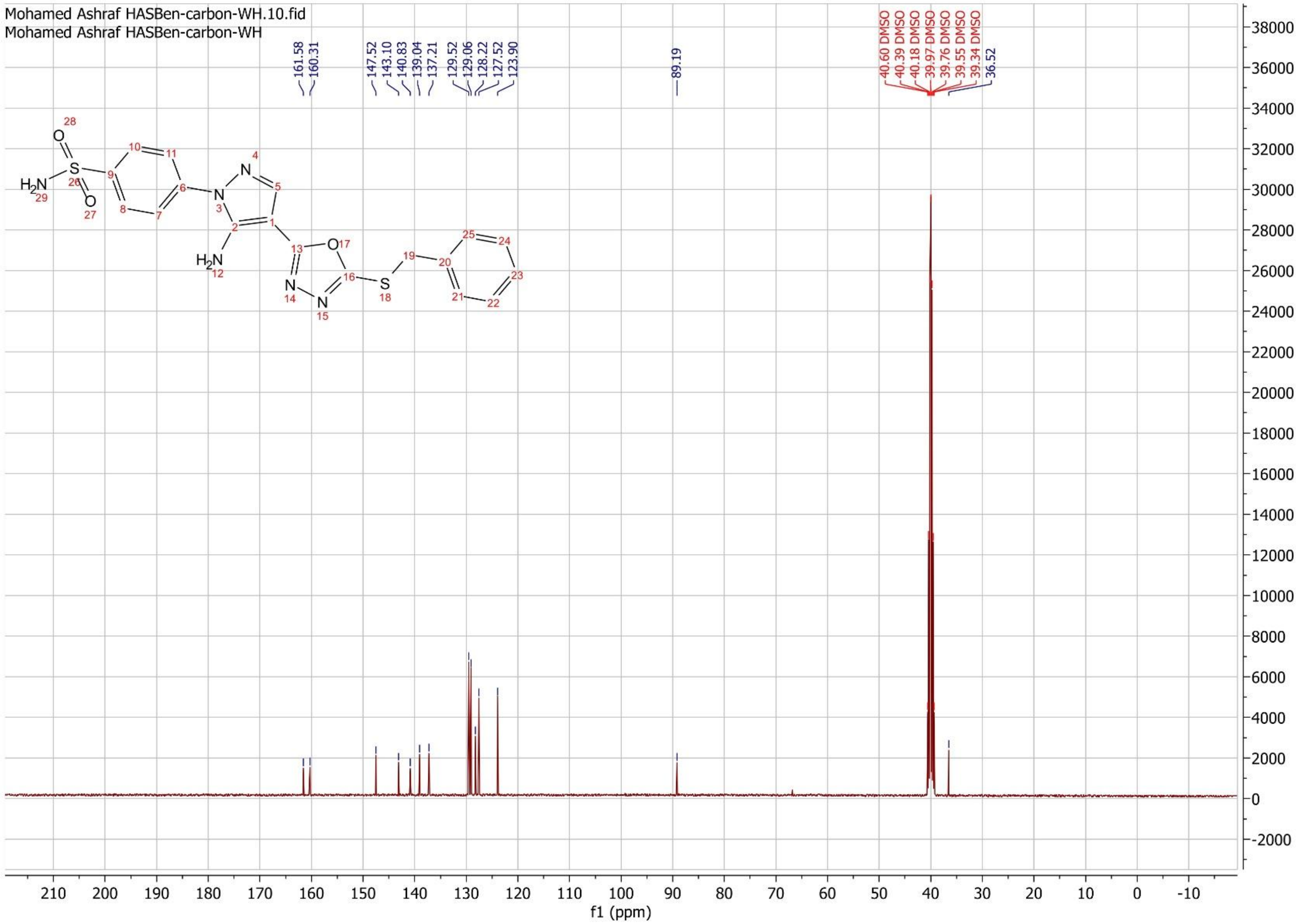

Figure S20. ^{13}C NMR spectrum of compound **10a**

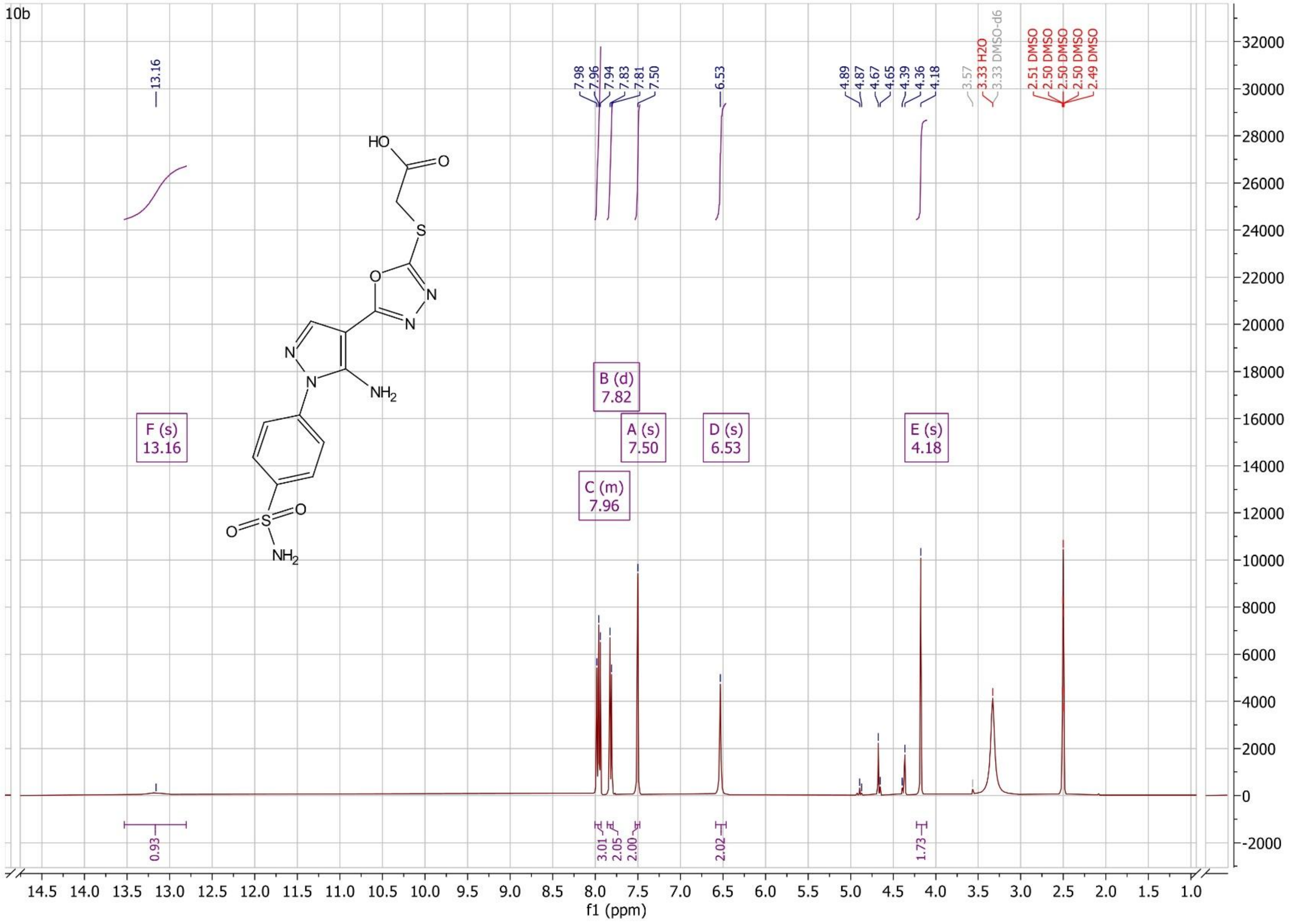

Figure S21. ^{1}H NMR spectrum of compound **10b**

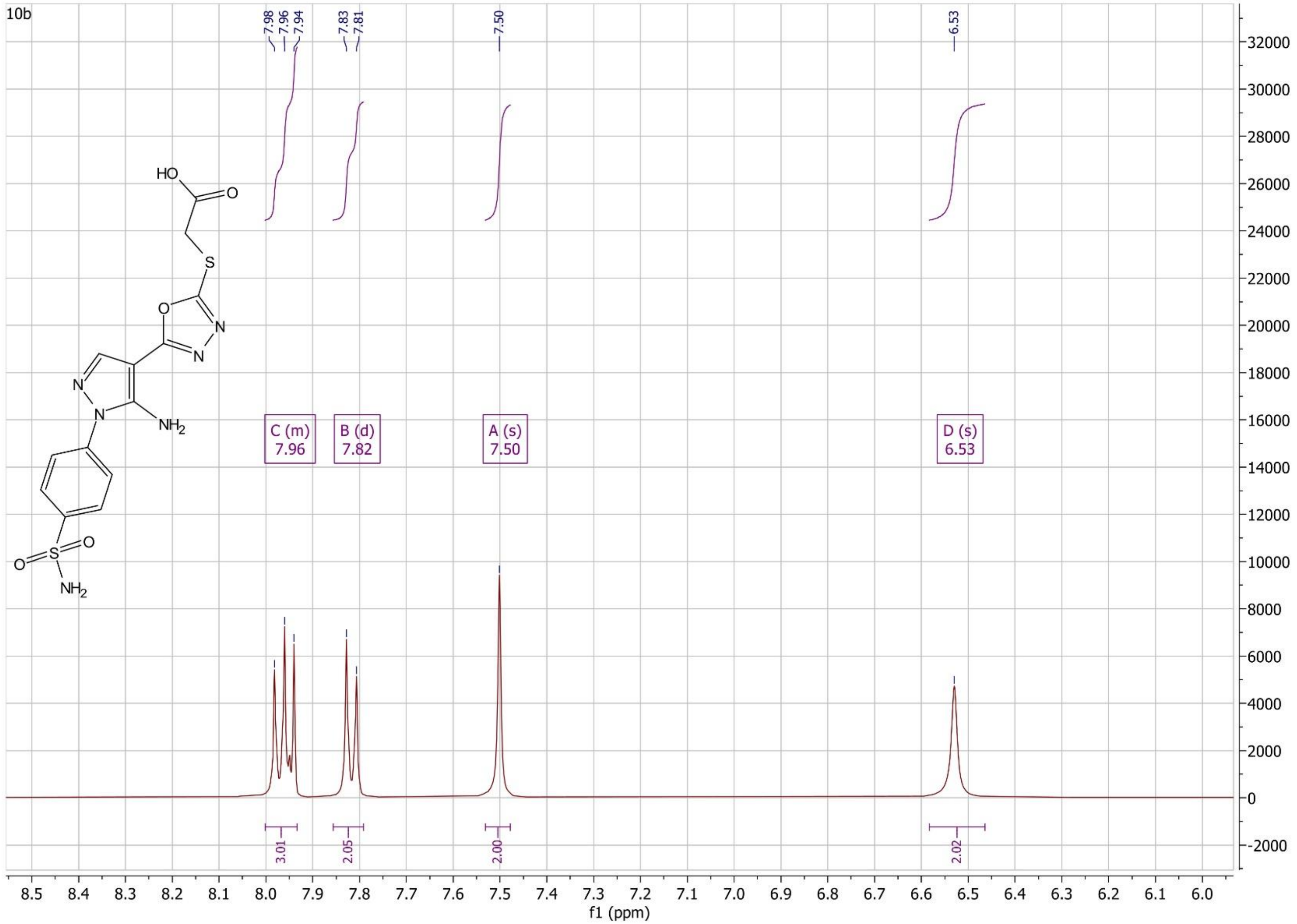

Figure S22. ^{1}H NMR spectrum of compound **10b**

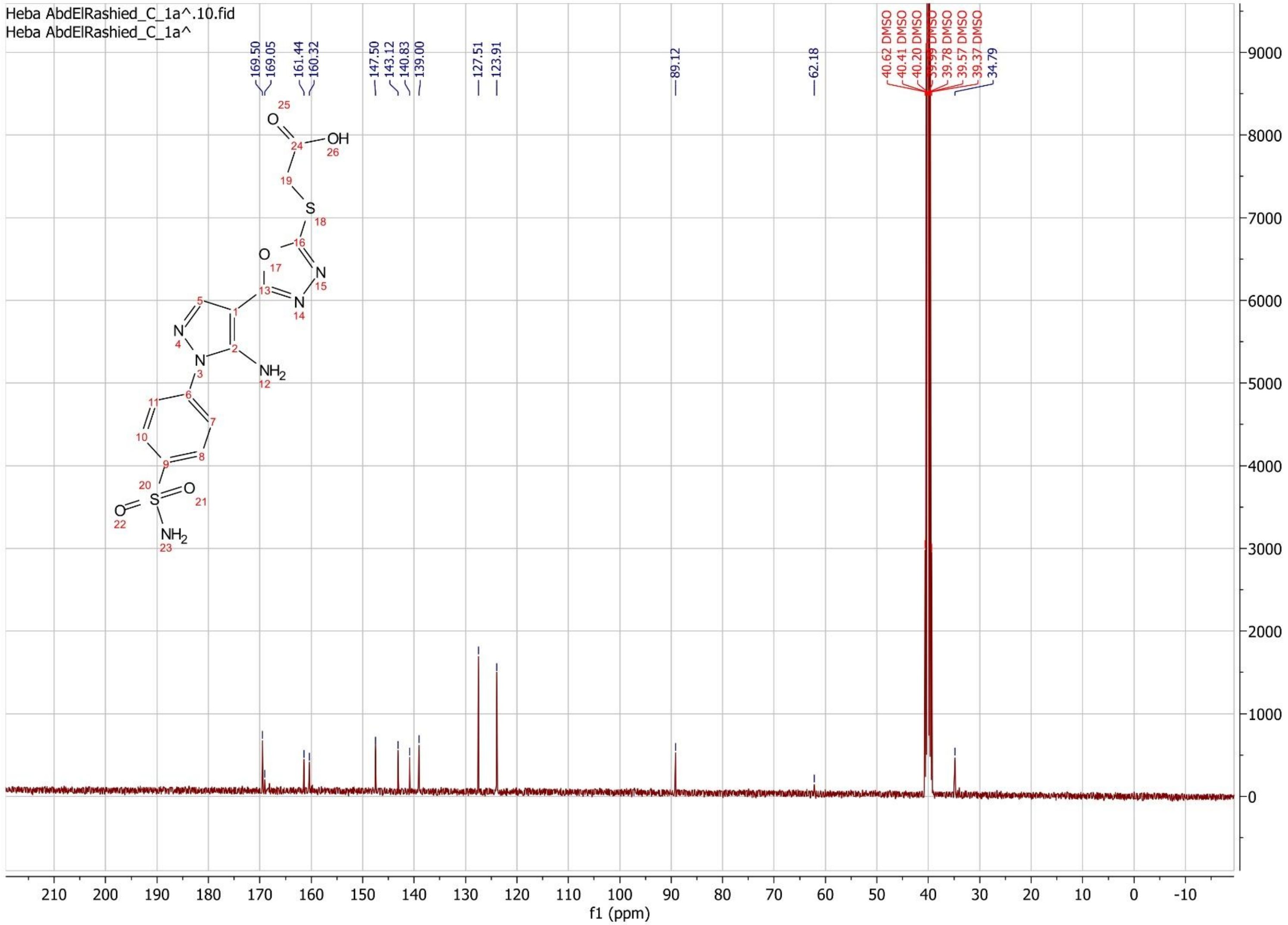

Figure S23. ^{13}C NMR spectrum of compound **10b**

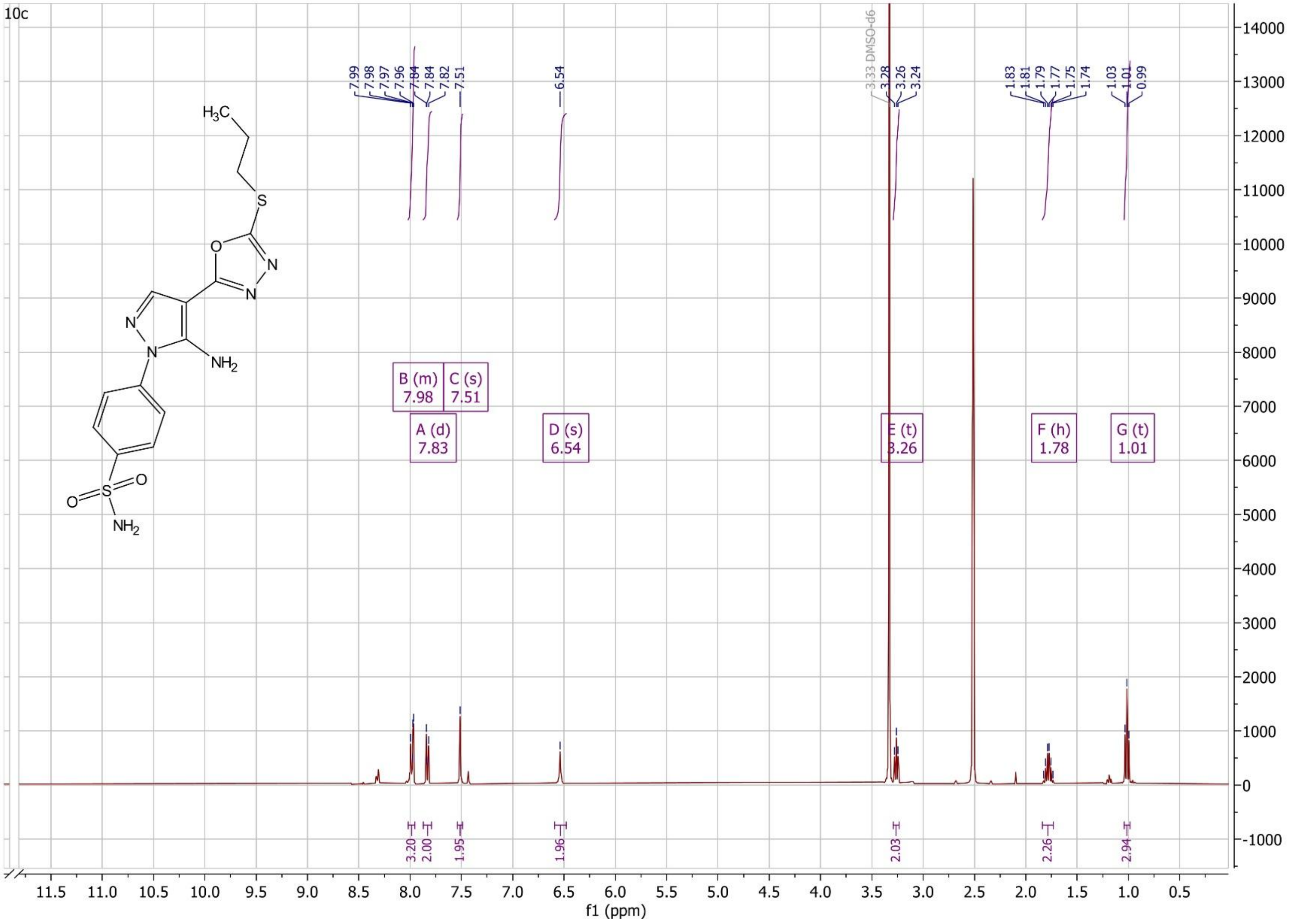

Figure S24. ^{1}H NMR spectrum of compound **10c**

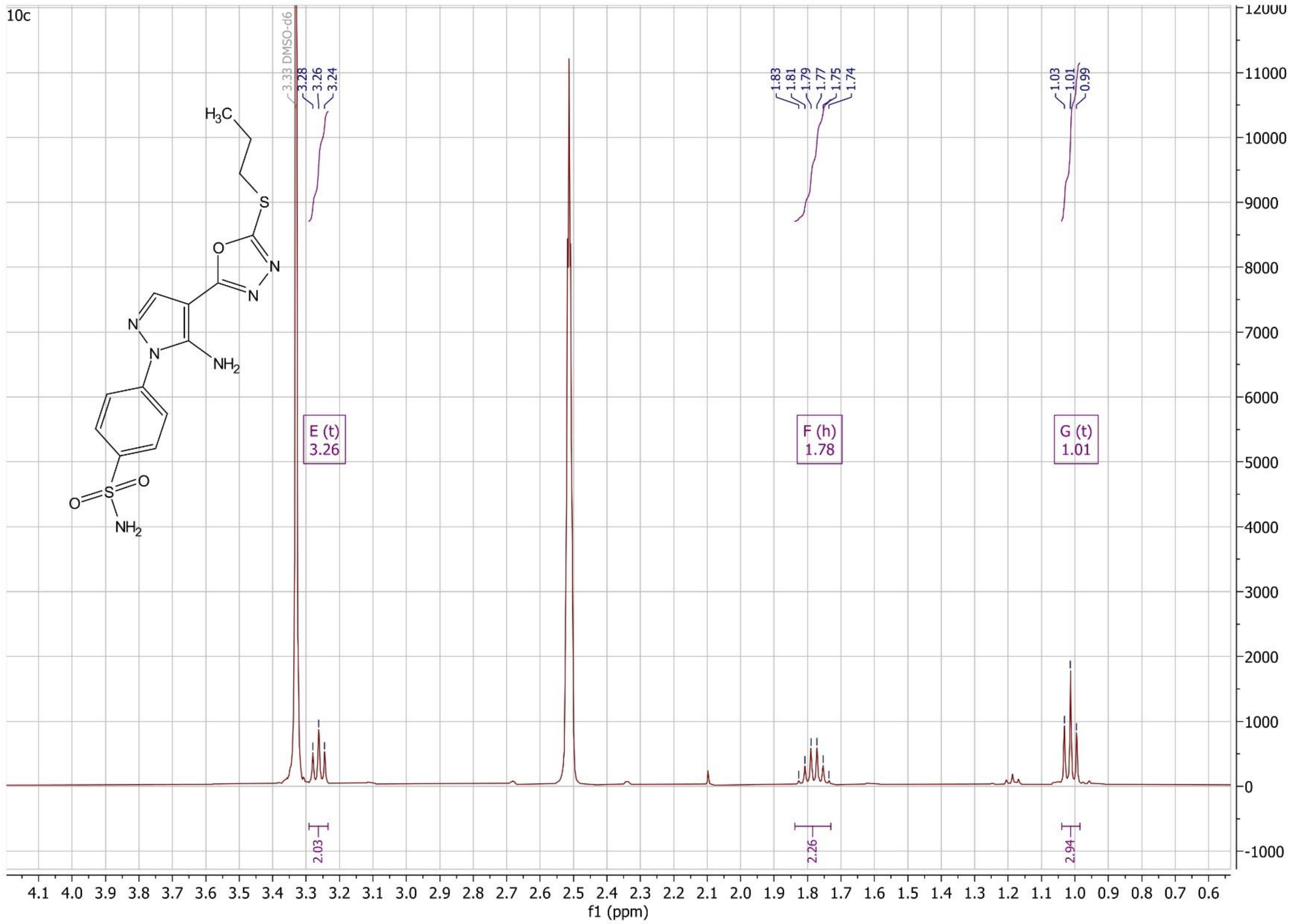

Figure S25. ^{1}H NMR spectrum of compound **10c**

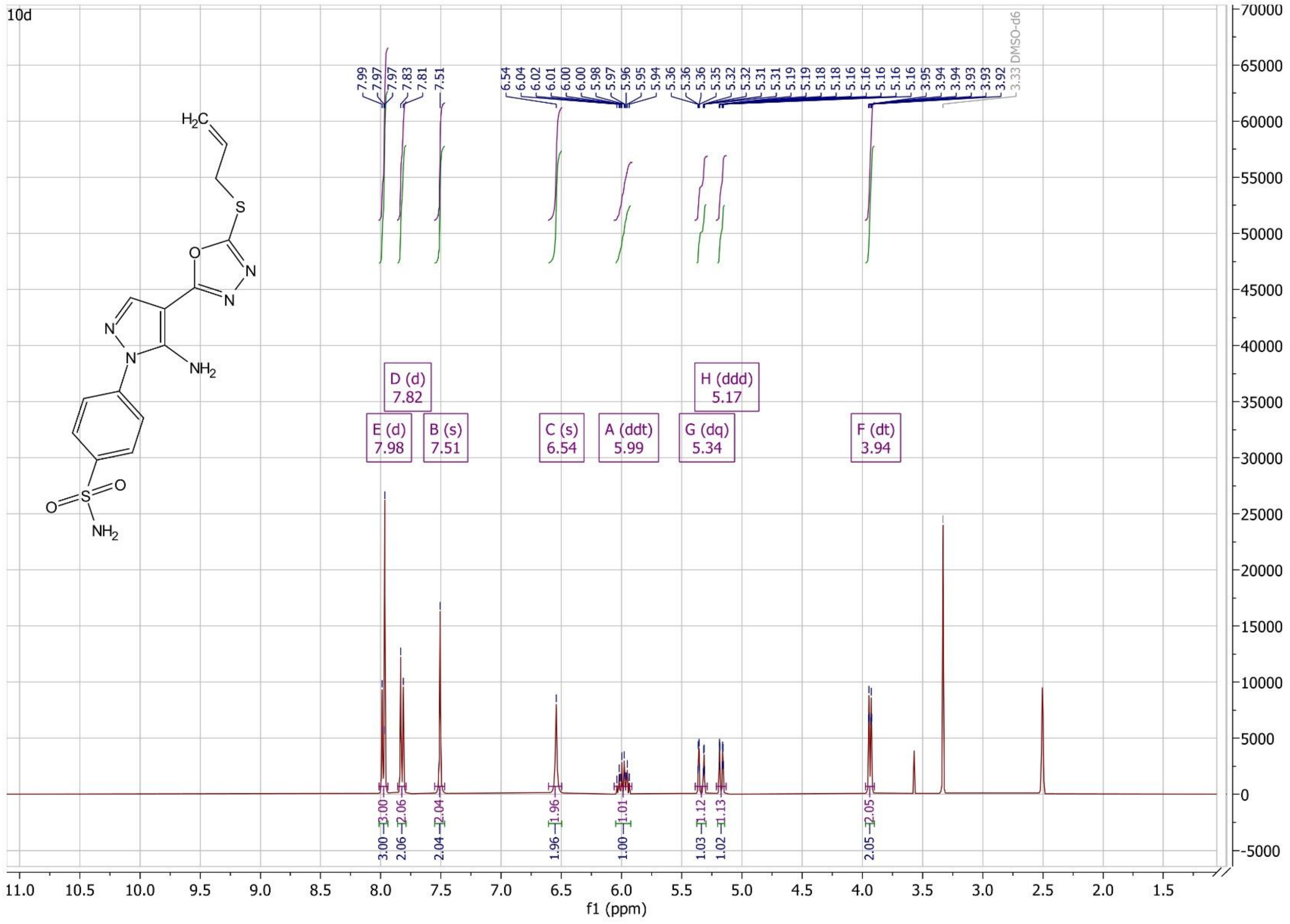

Figure S26. ^{1}H NMR spectrum of compound **10d**

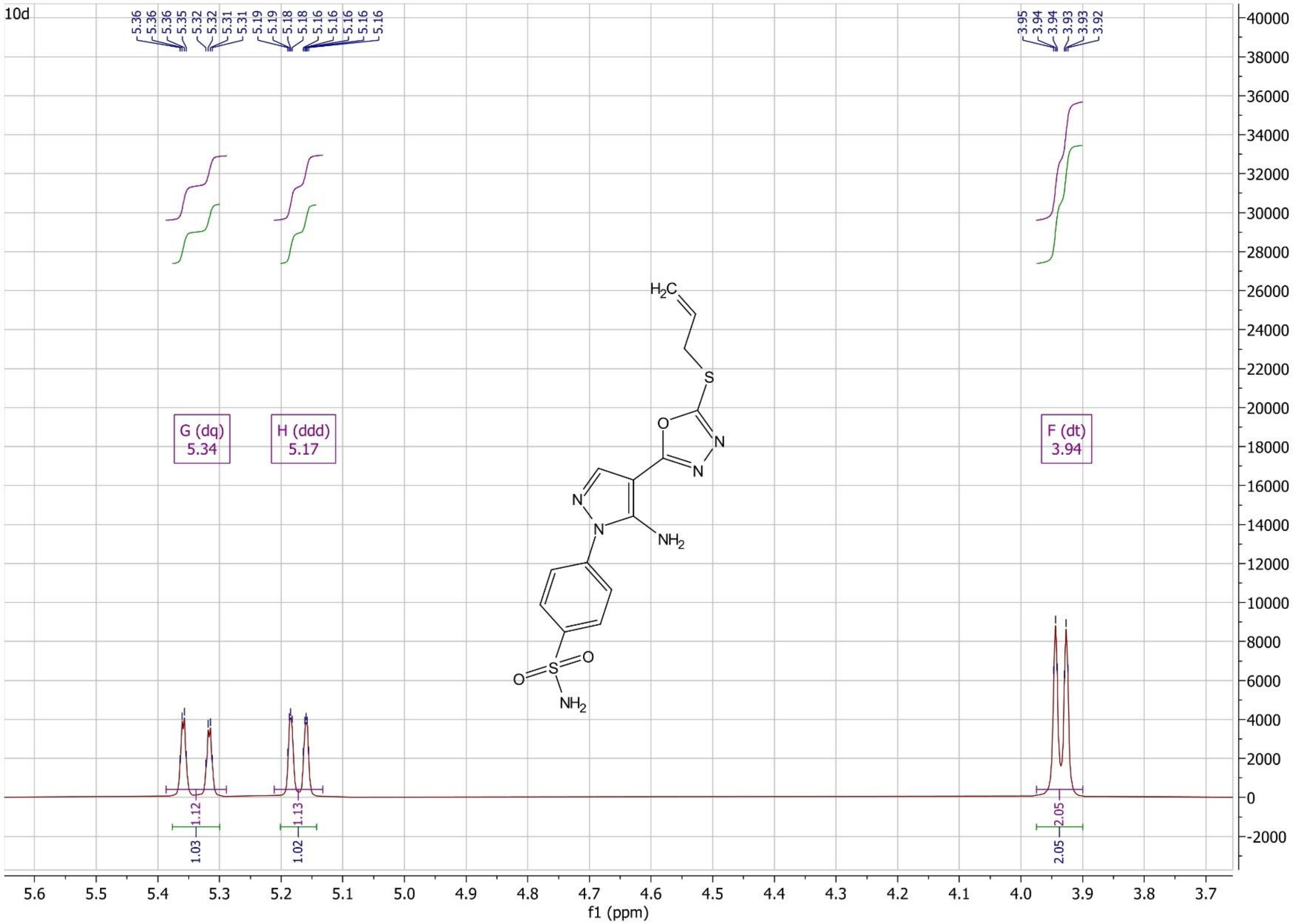

Figure S27. ^{1}H NMR spectrum of compound **10d**

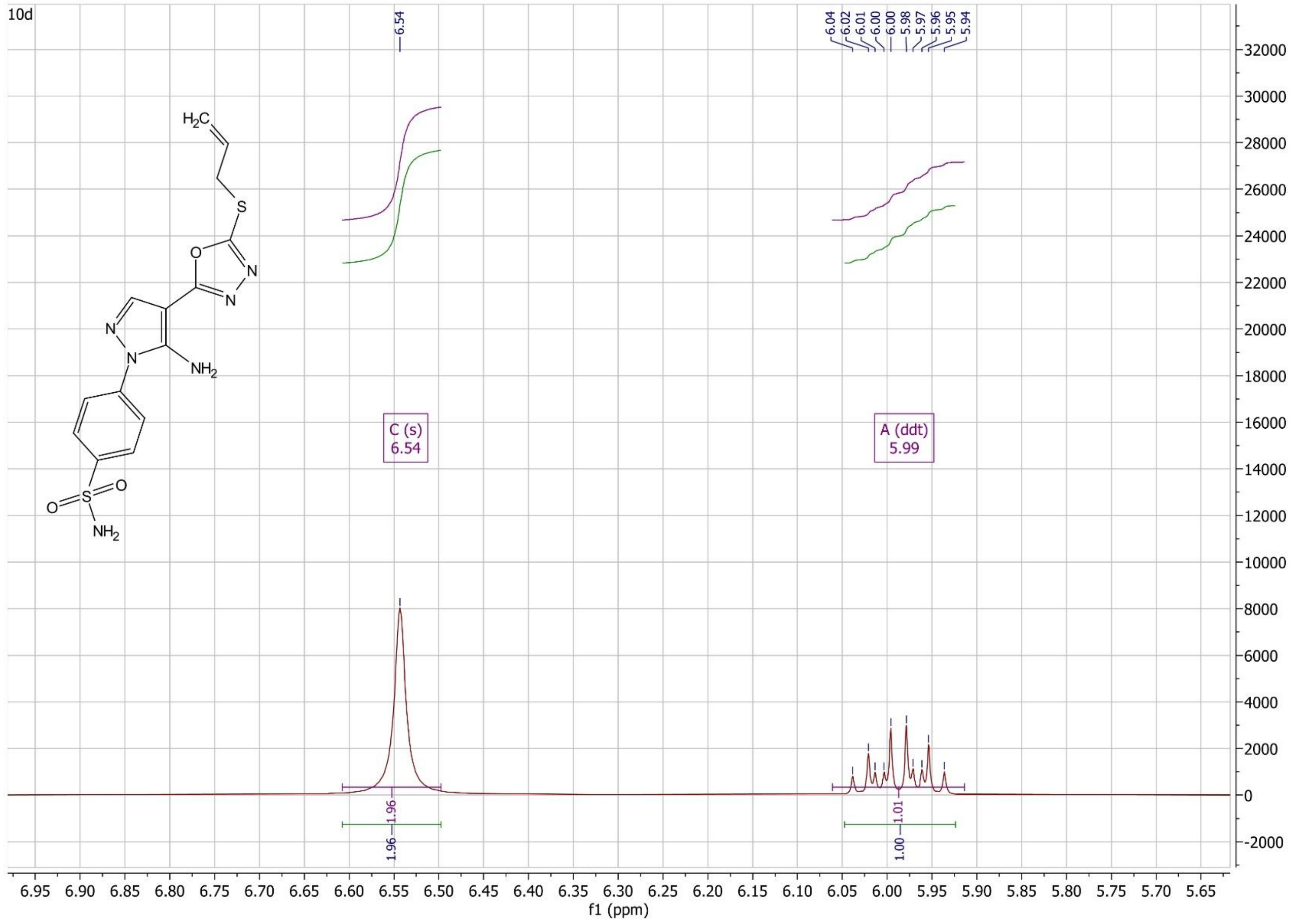

Figure S28. ^{1}H NMR spectrum of compound **10d**

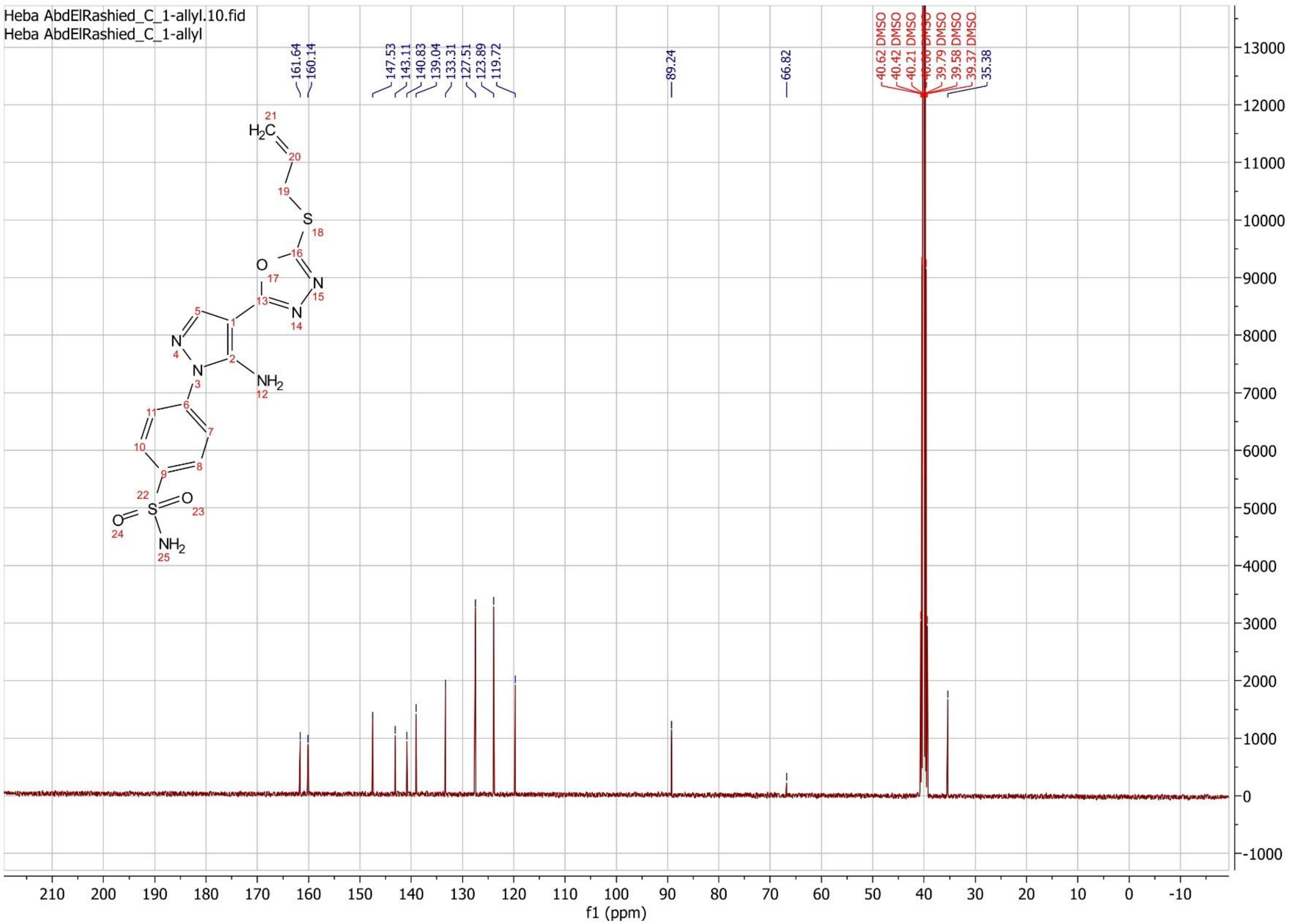

Figure S29. ^{13}C NMR spectrum of compound **10d**

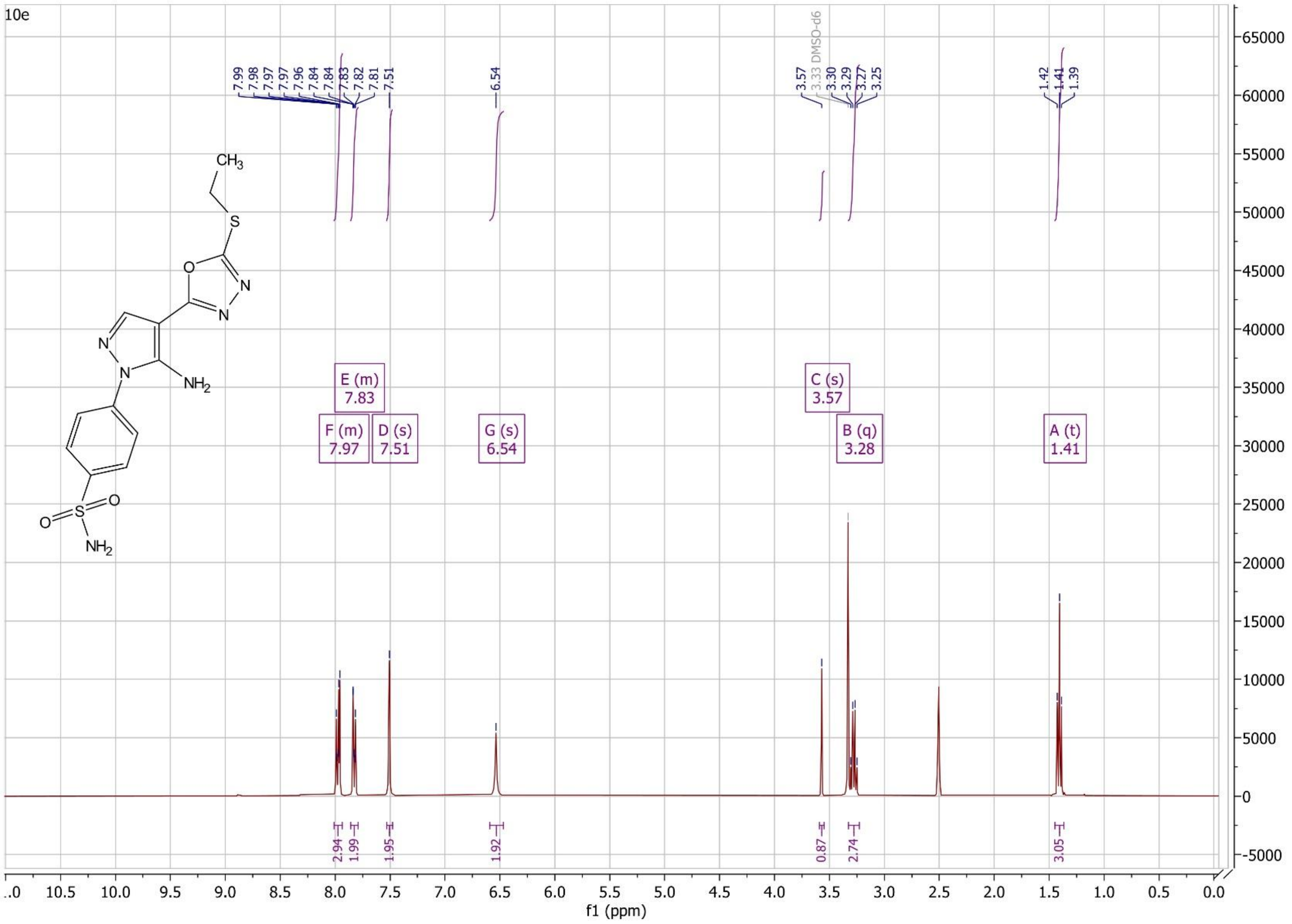

Figure S30. ^{1}H NMR spectrum of compound **10e**

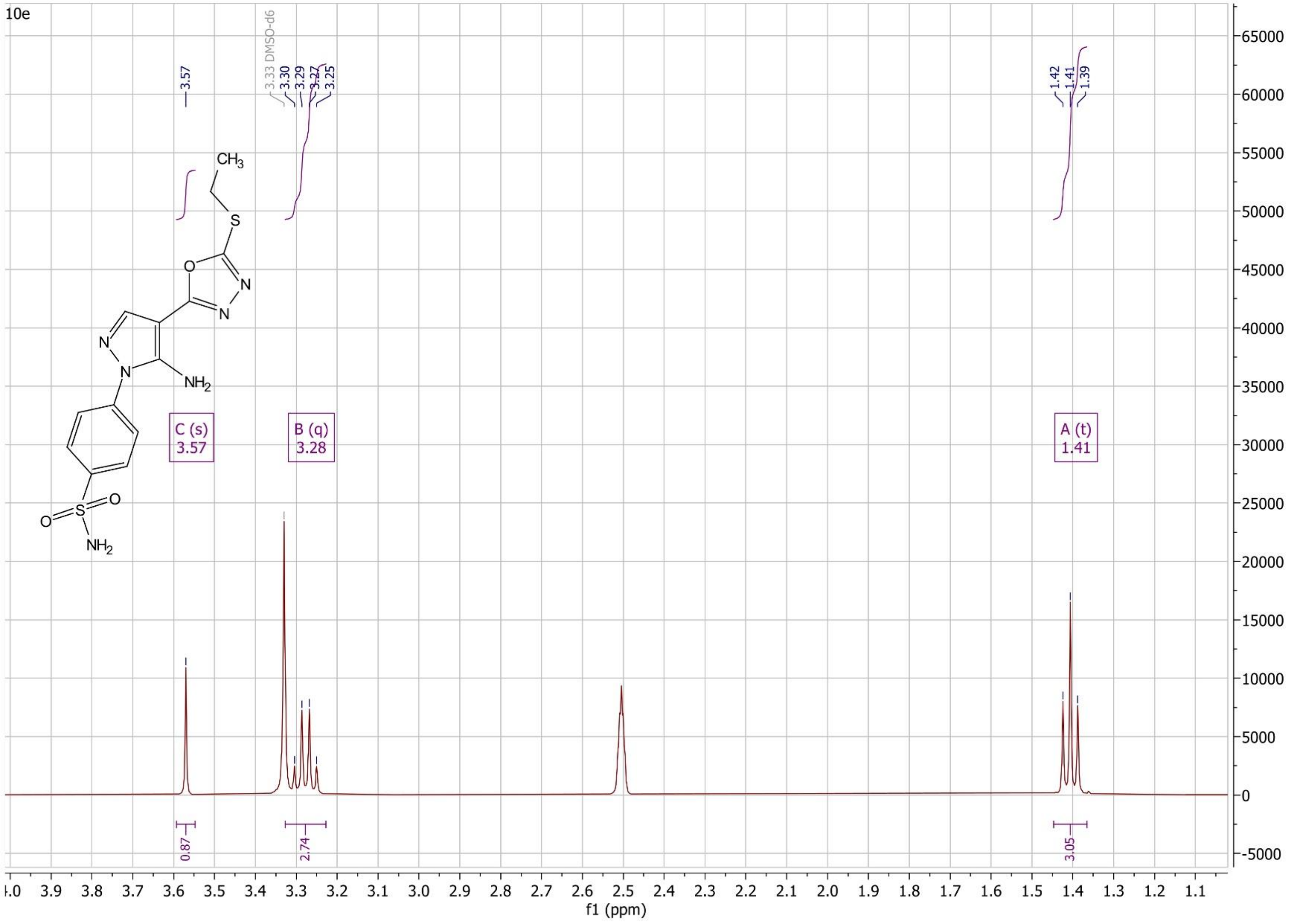

Figure S31. ^{1}H NMR spectrum of compound **10e**

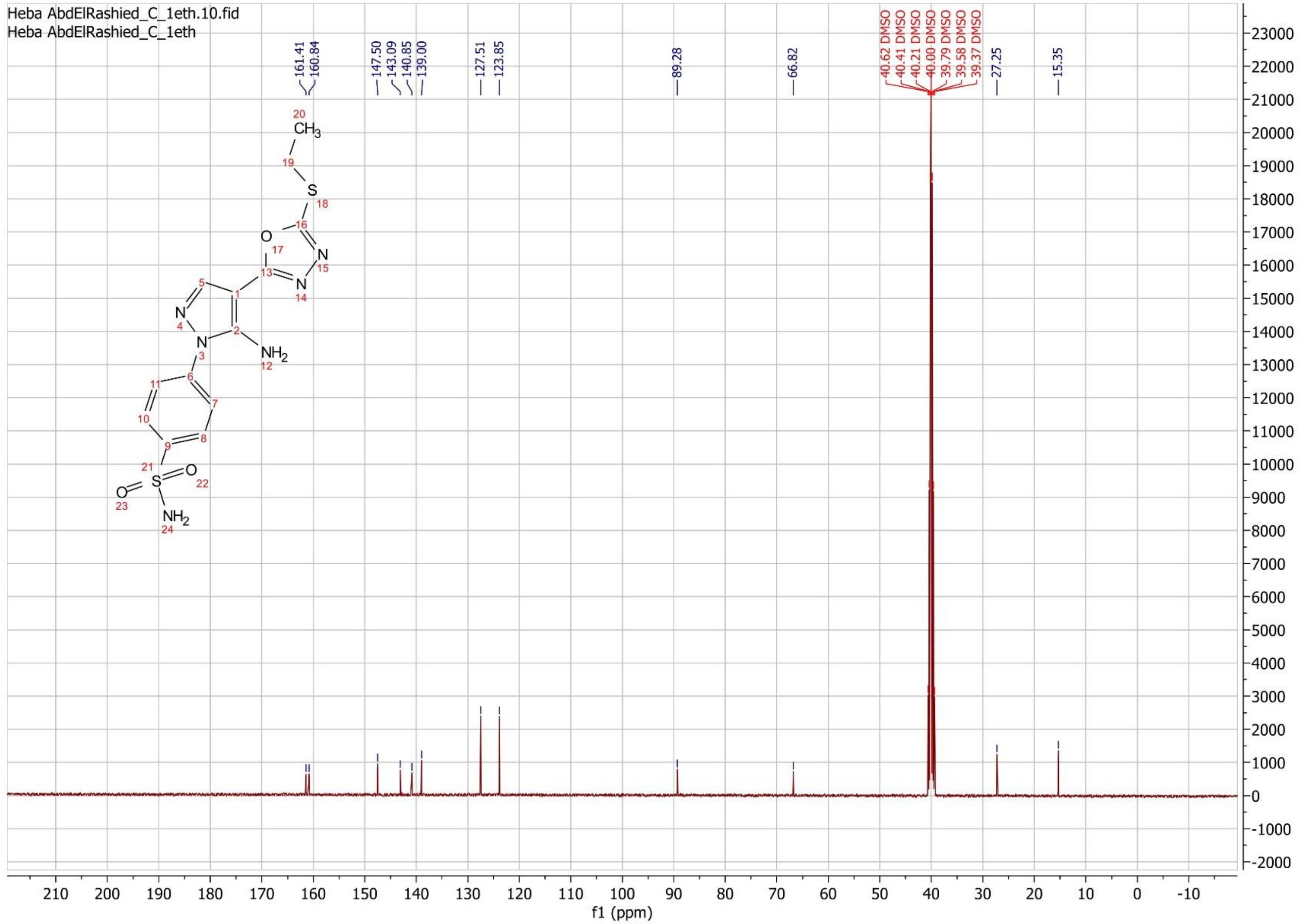

Figure S32. ^{13}C NMR spectrum of compound **10e**

2. HRMS spectra of the final compounds

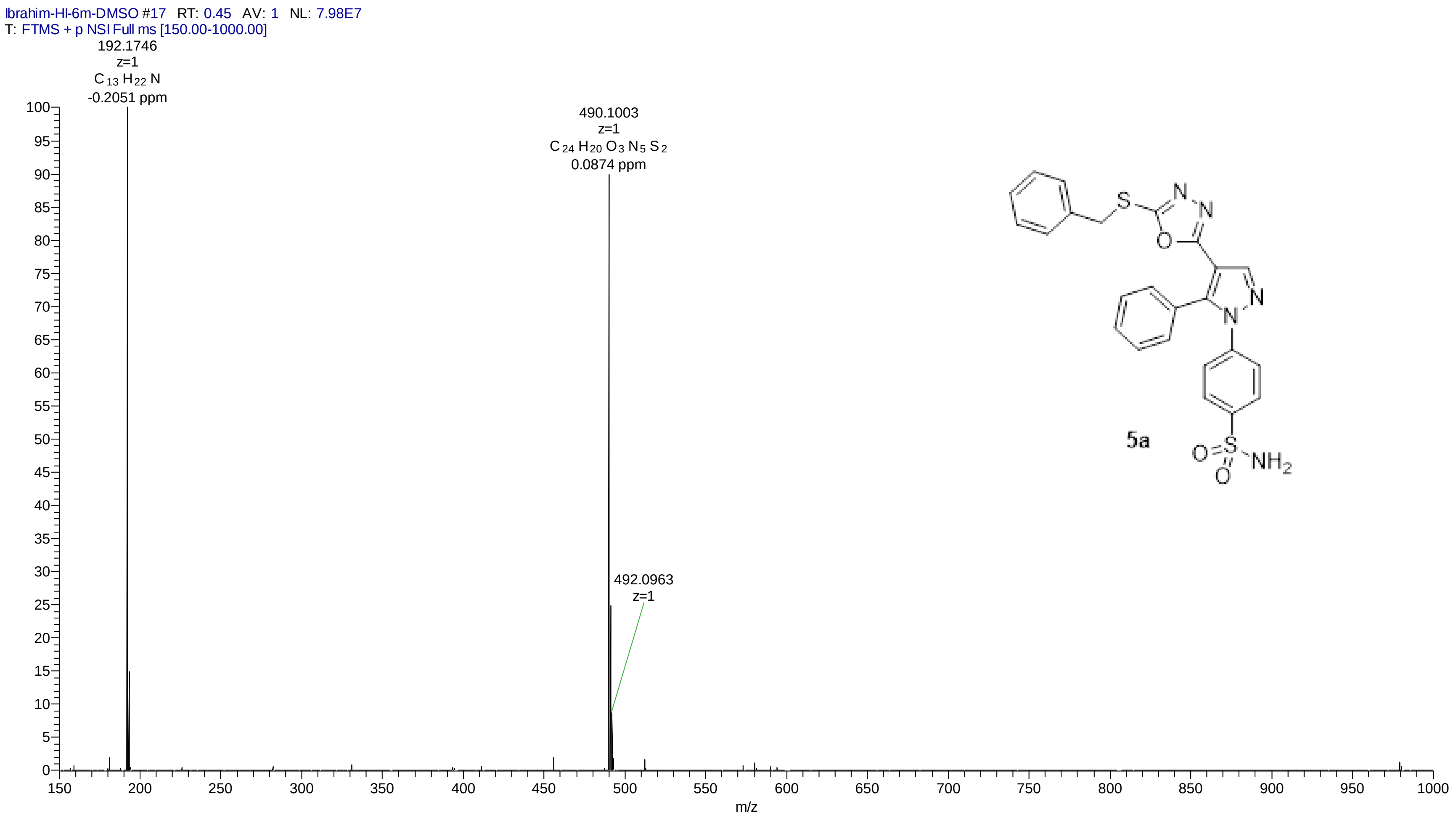

Figure S33. HRMS spectrum of compound **5a**

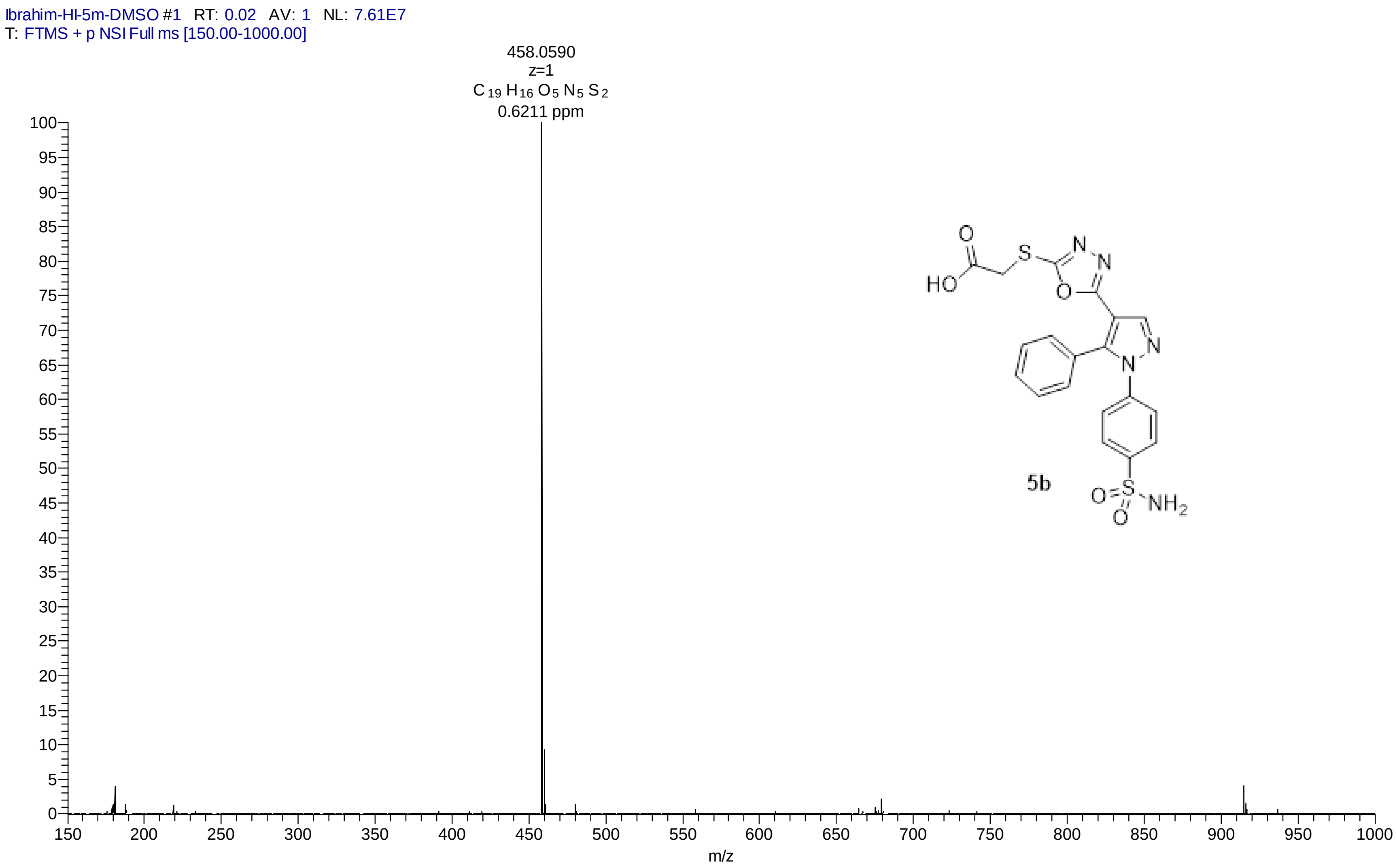

Figure S34. HRMS spectrum of compound **5b**

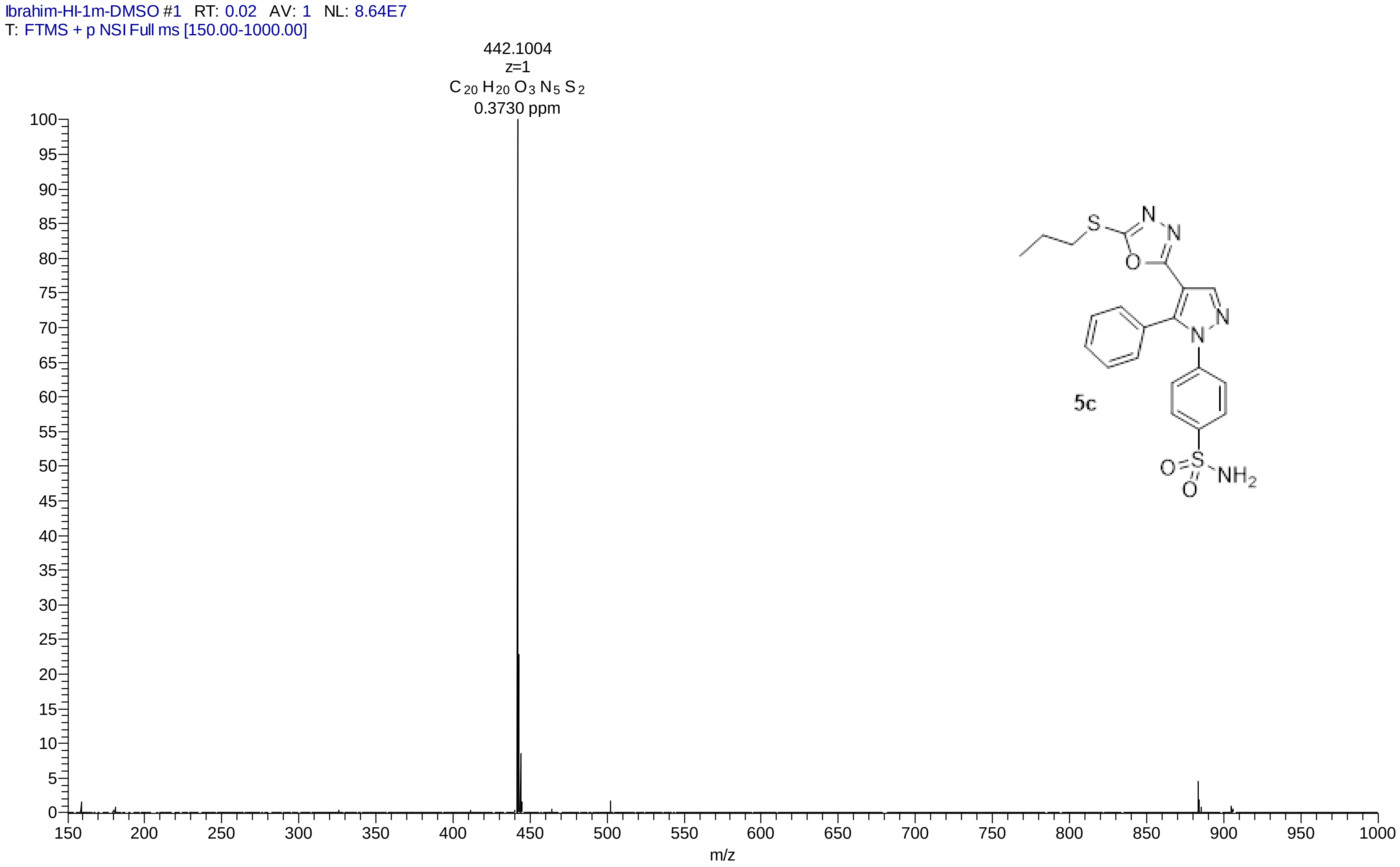

Figure S35. HRMS spectrum of compound **5c**

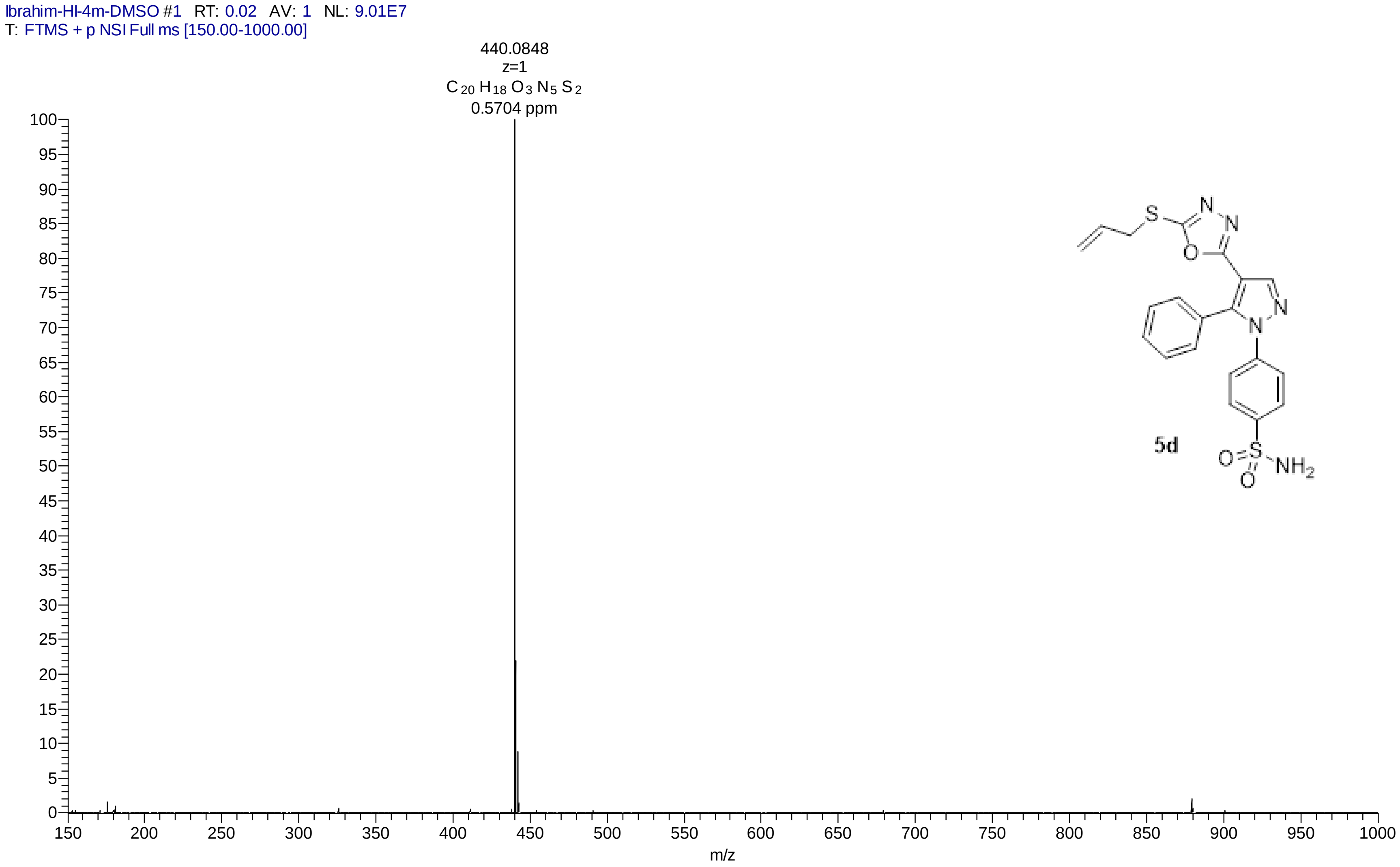

5d

Figure S36. HRMS spectrum of compound **5d**

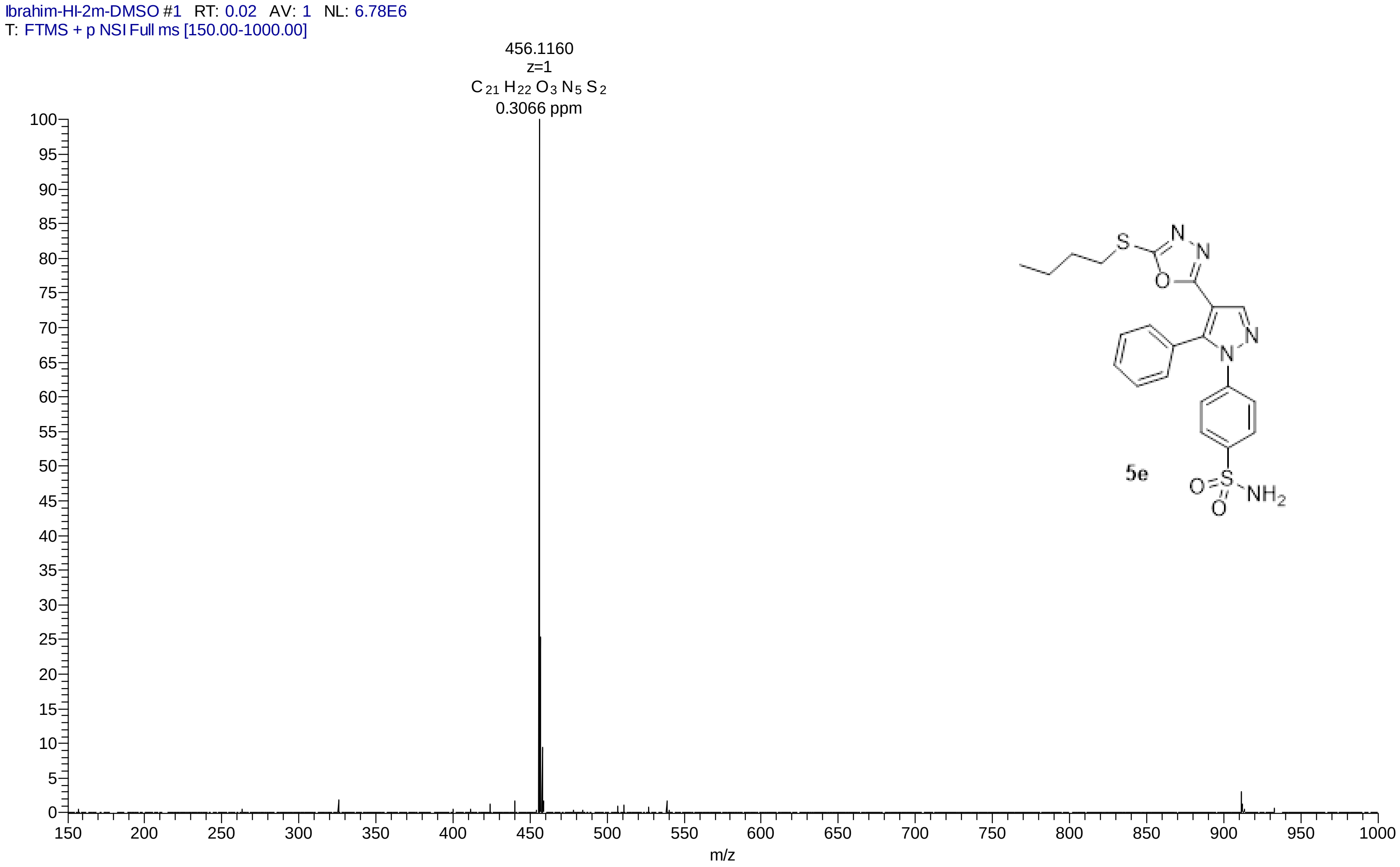

Figure S37. HRMS spectrum of compound **5e**

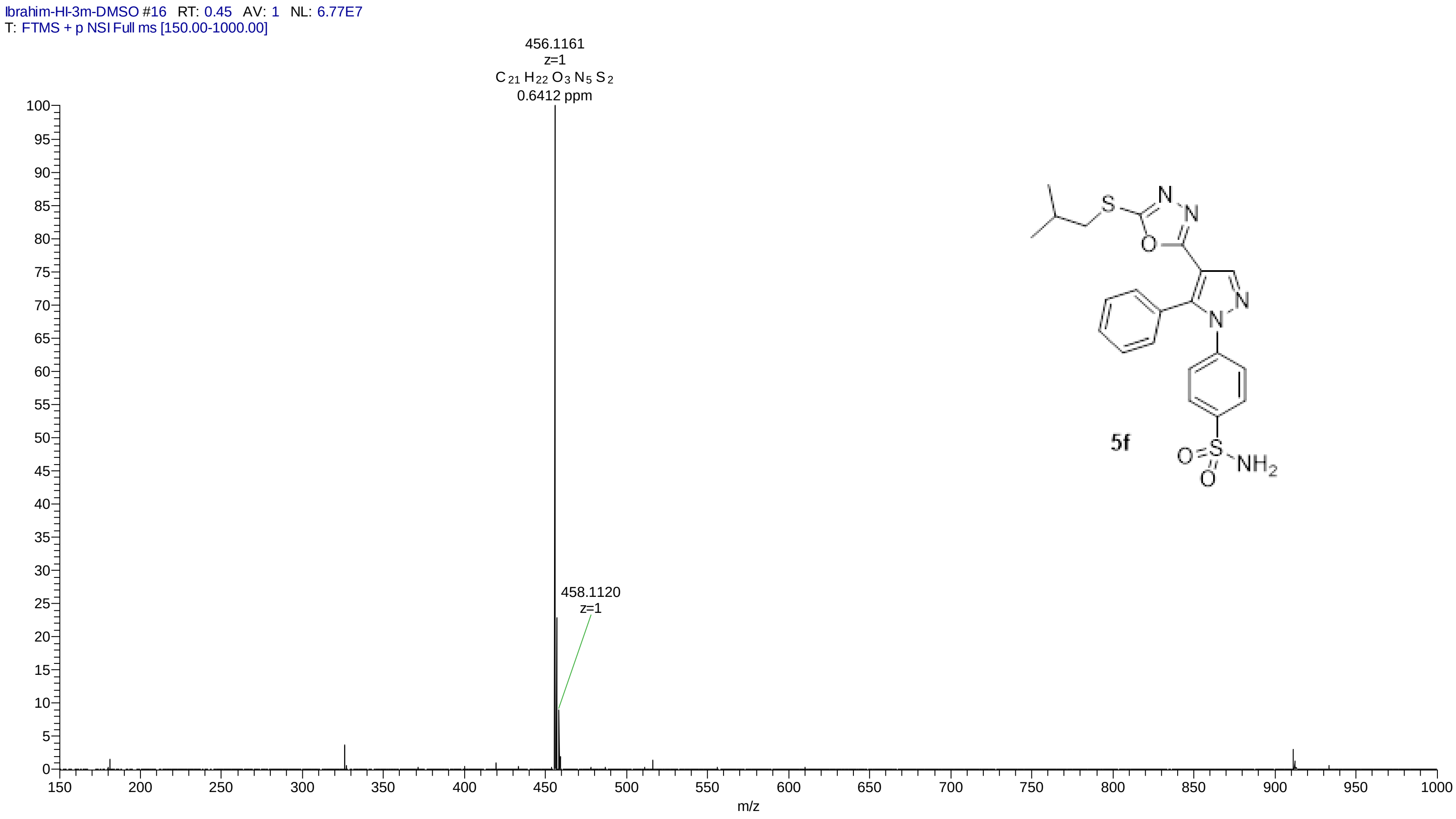

5f

Figure S38. HRMS spectrum of compound **5f**

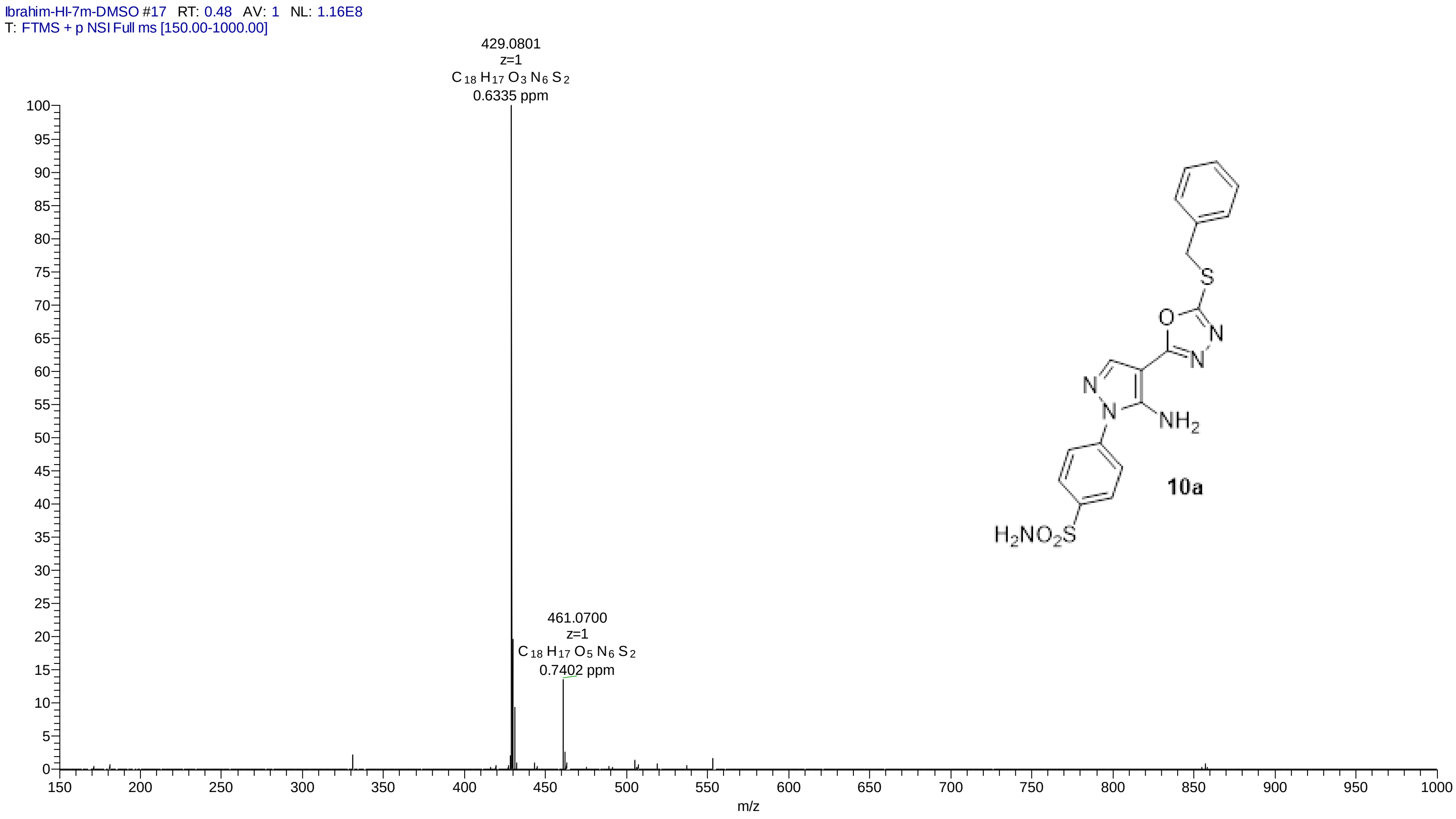

Figure S39. HRMS spectrum of compound **10a**

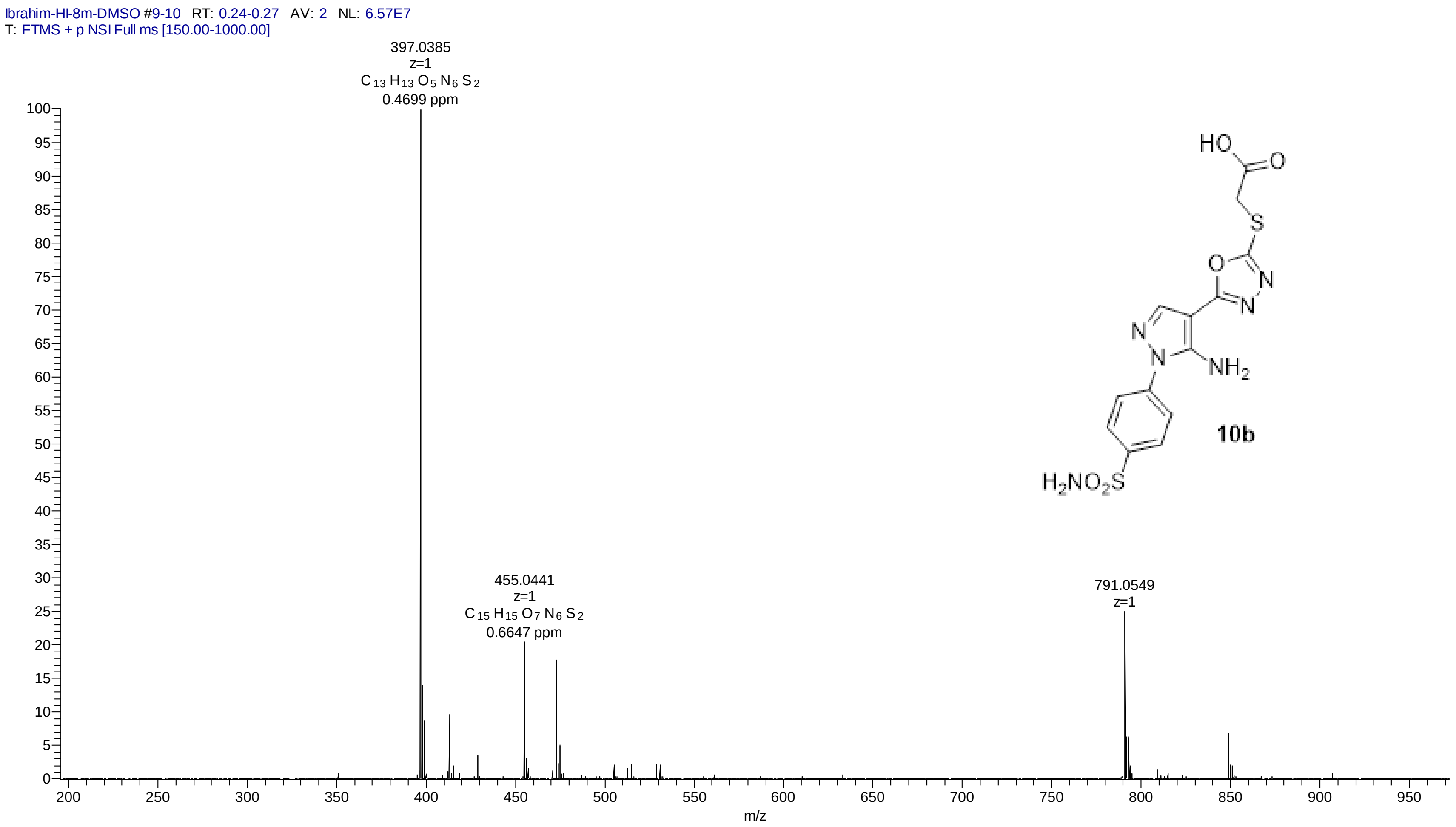

Figure S40. HRMS spectrum of compound **10b**

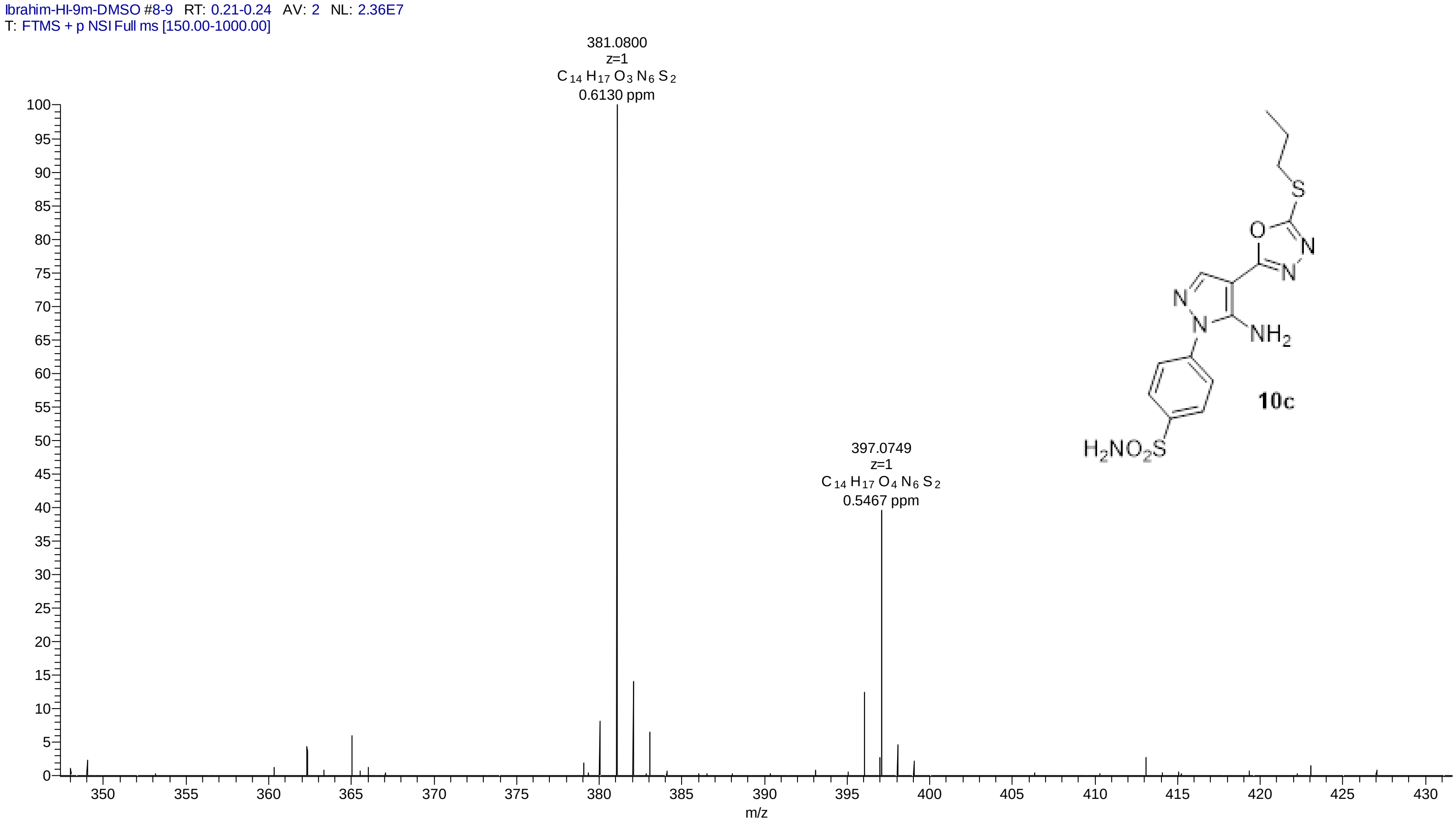

Figure S41. HRMS spectrum of compound **10c**

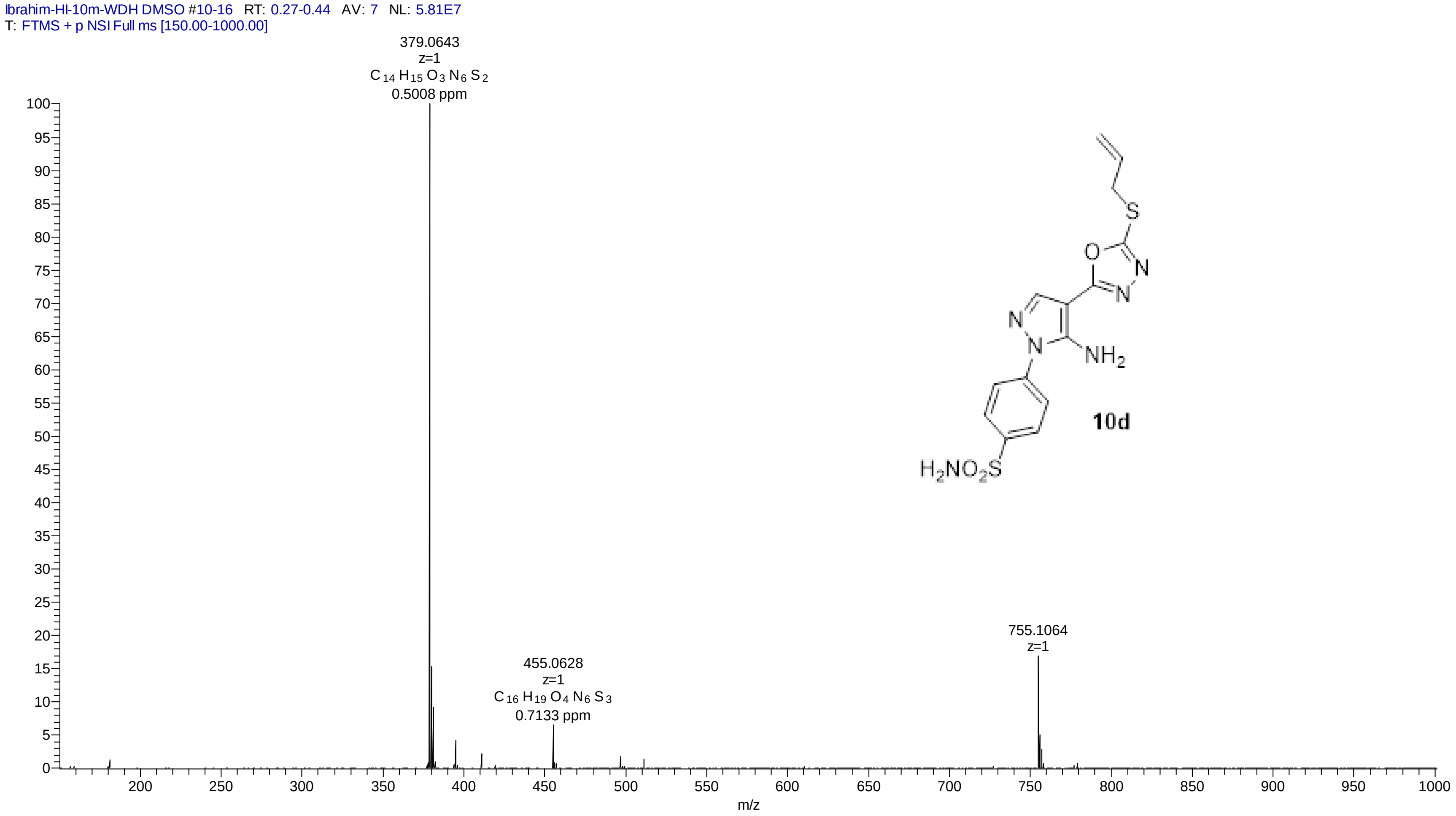

Figure S42. HRMS spectrum of compound **10d**

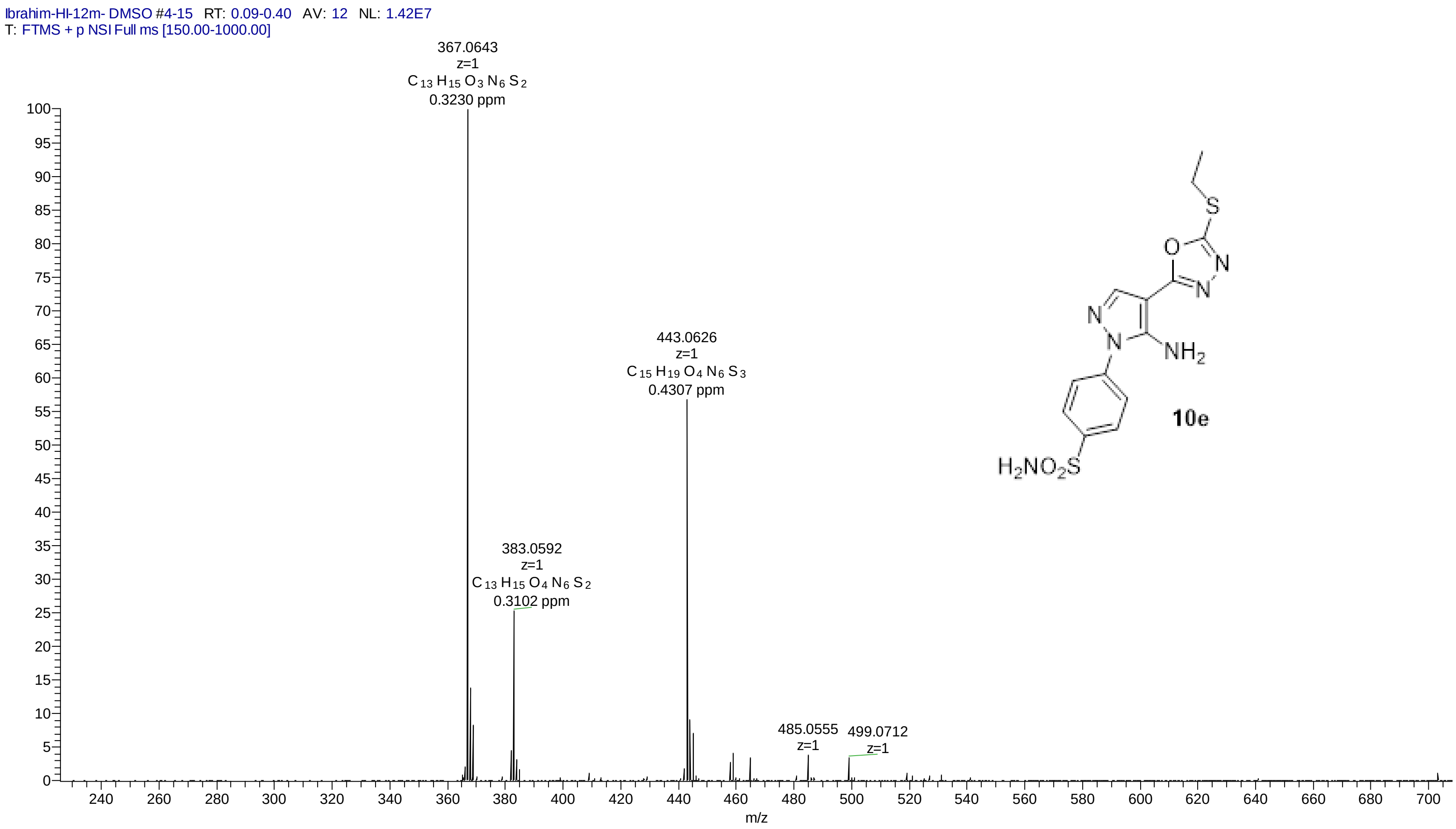

Figure S43. HRMS spectrum of compound **10e**

3. HPLC Purity Charts

HPLC Purity Charts

22.05.2026 10:38:55 Page 1 / 1

Analysenreport

Sample Information

Sample Name	:
Tray#	: 0
Vial#	: 5
Injection Volume	: 10
Data File	: HI-6m_220526.lcd
Method File	: MSP5-95_30min_1.0.lcm
Batch File	: B_220526.lcb
Report Format File	: Reportformat2.lsr
Date Acquired	: 22.05.2026 10:05:54
Date Processed	: 22.05.2026 10:35:57
Comment	: MeOH+H2O+0,05%TFA

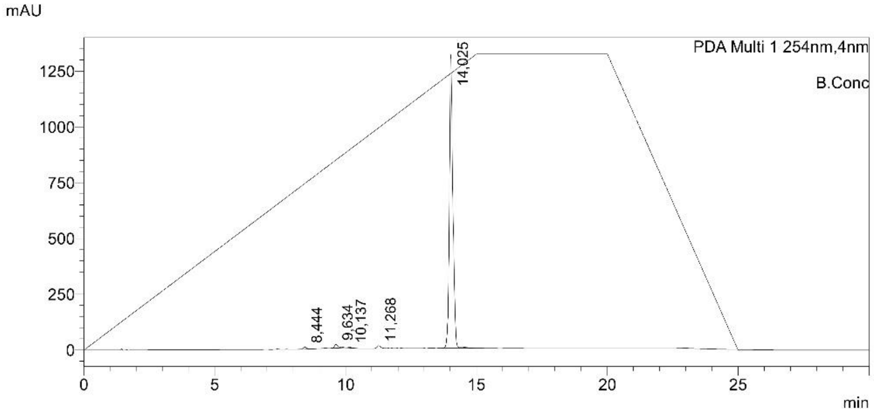

PDA Ch1 254nm

Peak#	Ret. Time	Area	Height	Area%
1	8,444	89344	9179	0,711
2	9,634	180615	16564	1,436
3	10,137	35520	4195	0,282
4	11,268	114529	12057	0,911
5	14,025	12153717	1317555	96,660
Total		12573726	1359549	100,000

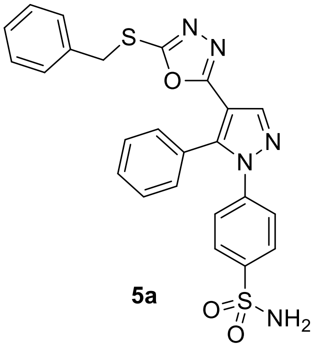

5a

D:\HPLC\Hani Ibrahim\HI-6m_220526.lcd

Figure S44. HPLC chromatogram of compound **5a**

Analysenreport

Sample Information

Sample Name	:
Tray#	: 2
Vial#	: 12
Injection Volume	: 10
Data File	: HI-5m_P1_F32.lcd
Method File	: MSP5-95_30min_1.0_96well.lcm
Batch File	: B_181225-1.lcb
Report Format File	: Reportformat2.lsr
Date Acquired	: 18.12.2025 23:20:57
Date Processed	: 18.12.2025 23:50:59
Comment	: MeOH/H2O/0,05%TFA

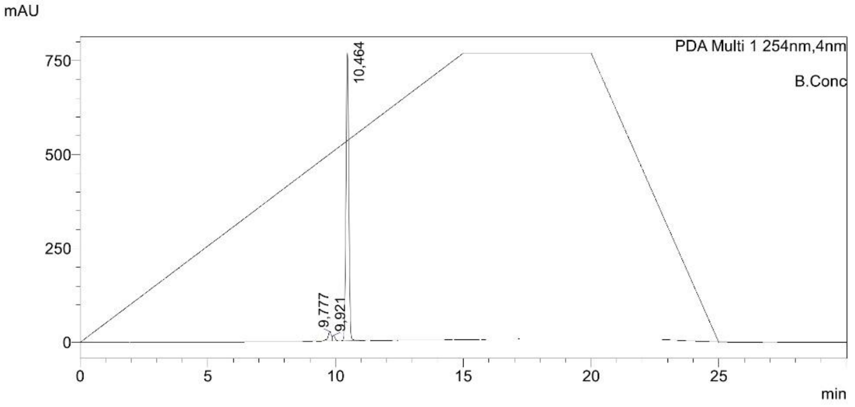

PDA Ch1 254nm

Peak#	Ret. Time	Area	Height	Area%
1	9,777	231899	25382	3,819
2	9,921	98584	14415	1,624
3	10,464	5741107	765245	94,557
Total		6071590	805042	100,000

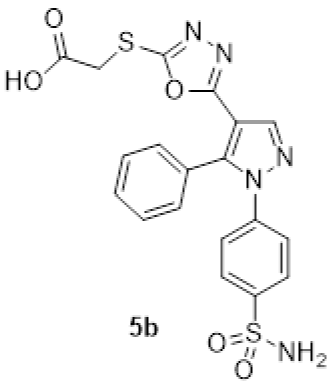

5b

Figure S45. HPLC chromatogram of compound **5b**

Analysenreport

Sample Information

Sample Name	:
Tray#	: 0
Vial#	: 1
Injection Volume	: 2
Data File	: HI_1m_a.lcd
Method File	: MSP5-95_30min_1.0.lcm
Batch File	: B_270426_1.lcb
Report Format File	: Reportformat2.lsr
Date Acquired	: 27.04.2026 09:09:28
Date Processed	: 27.04.2026 09:39:32
Comment	: MeOH+H2O+0,05%TFA

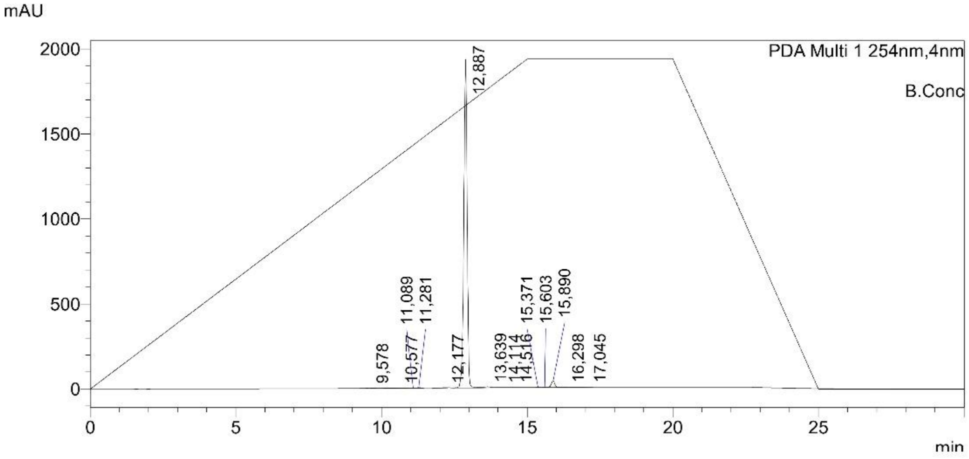

PDA Ch1 254nm

Peak#	Ret. Time	Area	Height	Area%
1	9,578	33349	2262	0,207
2	10,577	31704	1138	0,197
3	11,089	35637	1975	0,221
4	11,281	32497	3145	0,202
5	12,177	37216	2102	0,231
6	12,887	15305060	1933884	95,077
7	13,639	93952	5744	0,584
8	14,114	33710	1780	0,209
9	14,516	52427	2631	0,326
10	15,371	30587	1775	0,190
11	15,603	20684	2090	0,128
12	15,890	331324	40348	2,058
13	16,298	19902	1465	0,124
14	17,045	39546	945	0,246
Total		16097595	2001284	100,000

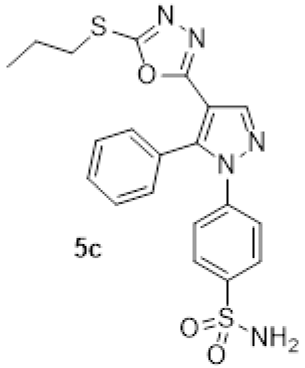

5c

D:\HPLC\Hani Ibrahim\HI_1m_a.lcd

Figure S46. HPLC chromatogram of compound **5c**

Analysenreport

Sample Information

Sample Name	:
Tray#	: 0
Vial#	: 2
Injection Volume	: 2
Data File	: HI_4m.lcd
Method File	: MSP5-95_30min_1.0.lcm
Batch File	: B_270426_1.lcb
Report Format File	: Reportformat2.lsr
Date Acquired	: 27.04.2026 09:40:01
Date Processed	: 27.04.2026 10:10:05
Comment	: MeOH+H2O+0,05%TFA

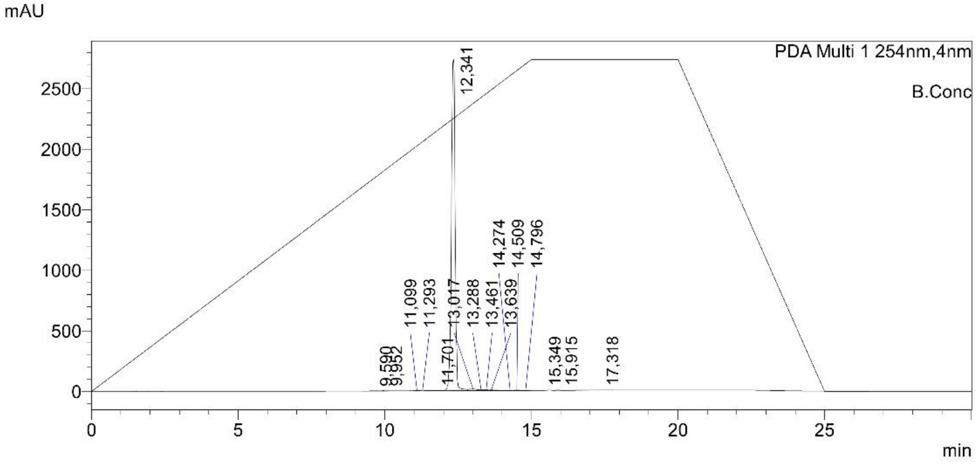

PDA Ch1 254nm

Peak#	Ret. Time	Area	Height	Area%
1	9,590	27722	2154	0,109
2	9,952	31119	2555	0,123
3	11,099	32996	3256	0,130
4	11,293	62112	6950	0,245
5	11,701	12090	1189	0,048
6	12,341	24469952	2734494	96,389
7	13,017	279453	12615	1,101
8	13,288	63979	6762	0,252
9	13,461	45205	5592	0,178
10	13,639	117495	5666	0,463
11	14,274	36846	2525	0,145
12	14,509	49174	2891	0,194
13	14,796	32030	1994	0,126
14	15,349	31547	1444	0,124
15	15,915	29253	1717	0,115
16	17,318	65605	735	0,258
Total		25386578	2792538	100,000

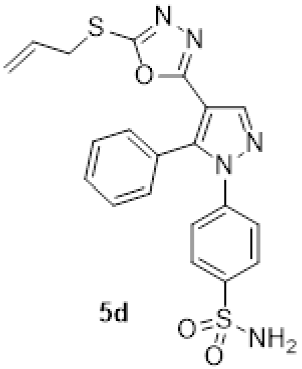

5d

Figure S47. HPLC chromatogram of compound **5d**

Analysenreport

Sample Information

Sample Name	:
Tray#	: 1
Vial#	: 4
Injection Volume	: 10
Data File	: HI-2m_P1_F4.lcd
Method File	: MSP5-95_30min_1.0_96well.lcm
Batch File	: B_171225-1.lcb
Report Format File	: Reportformat2.lsr
Date Acquired	: 17.12.2025 19:42:44
Date Processed	: 17.12.2025 20:12:47
Comment	: MeOH/H2O/0,05%TFA

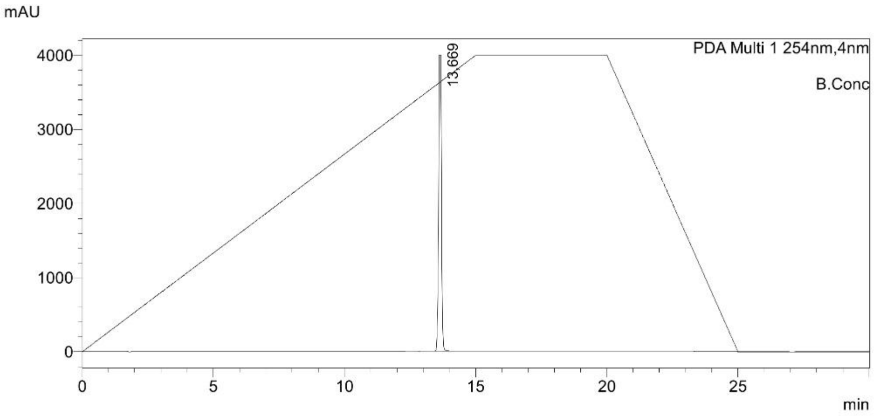

PDA Ch1 254nm

Peak#	Ret. Time	Area	Height	Area%
1	13,669	31931576	3992208	100,000
Total		31931576	3992208	100,000

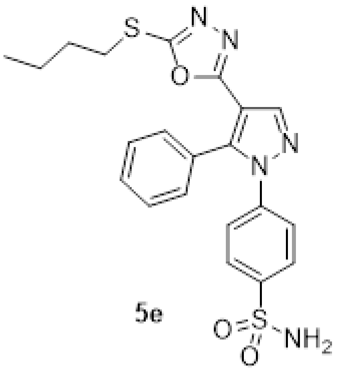

5e

D:\HPLC\Hani Ibrahim\HI-2m_P1_F4.lcd

Figure S48. HPLC chromatogram of compound **5e**

Analysenreport

Sample Information

Sample Name	:
Tray#	: 2
Vial#	: 2
Injection Volume	: 10
Data File	: HI-3m_P1_F22.lcd
Method File	: MSP5-95_30min_1.0_96well.lcm
Batch File	: B_181225-1.lcb
Report Format File	: Reportformat2.lsr
Date Acquired	: 18.12.2025 18:15:29
Date Processed	: 18.12.2025 18:45:31
Comment	: MeOH/H2O/0,05%TFA

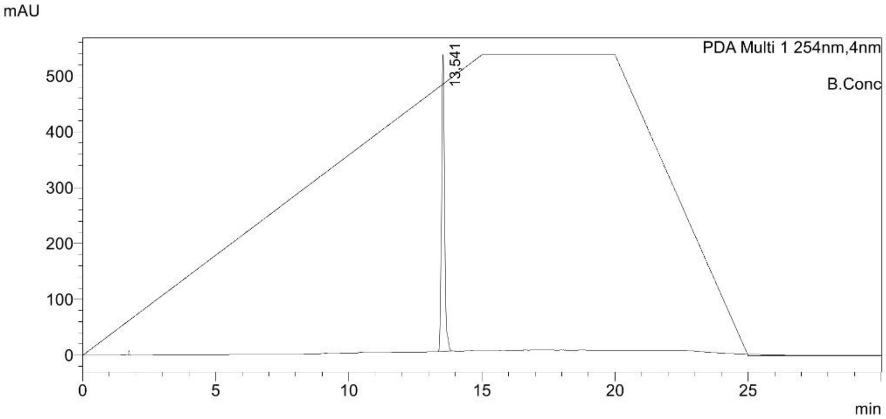

PDA Ch1 254nm

Peak#	Ret. Time	Area	Height	Area%
1	13,541	4269852	531291	100,000
Total		4269852	531291	100,000

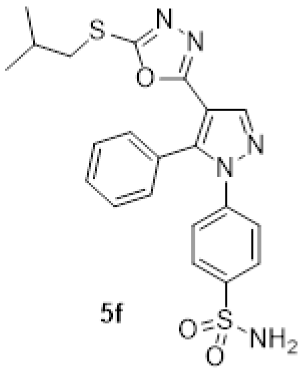

5f

D:\HPLC\Hani Ibrahim\HI-3m_P1_F22.lcd

Figure S49. HPLC chromatogram of compound **5f**

Analysenreport

Sample Information

Sample Name	:
Tray#	: 0
Vial#	: 6
Injection Volume	: 10
Data File	: HI-11m_220526_WDH.lcd
Method File	: MSP5-95_30min_1.0.lcm
Batch File	: B_220526.lcb
Report Format File	: Reportformat2.lsr
Date Acquired	: 22.05.2026 12:38:35
Date Processed	: 22.05.2026 13:08:39
Comment	: MeOH+H2O+0,05%TFA

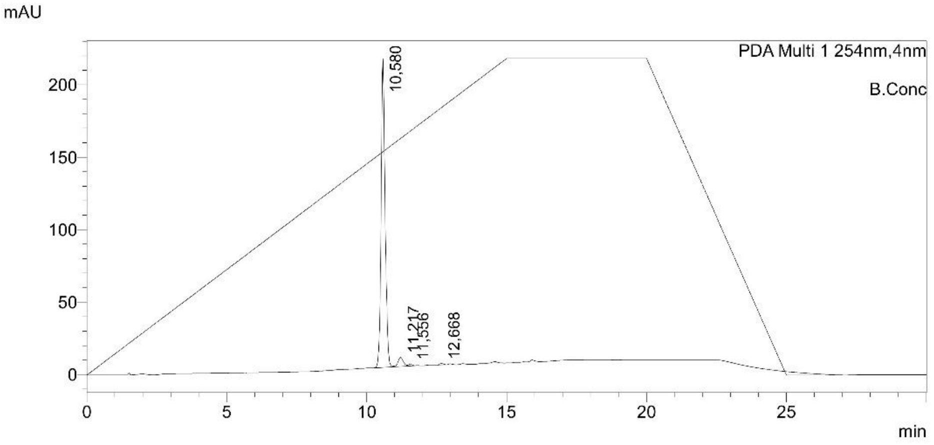

PDA Ch1 254nm

Peak#	Ret. Time	Area	Height	Area%
1	10,580	2119097	213165	95,369
2	11,217	81661	6209	3,675
3	11,556	11582	1334	0,521
4	12,668	9647	1122	0,434
Total		2221987	221830	100,000

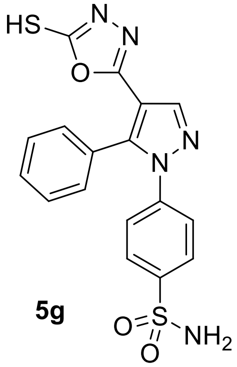

5g

D:\HPLC\Hani Ibrahim\HI-11m_220526_WDH.lcd

Figure S50. HPLC chromatogram of compound **5g**

Analysenreport

Sample Information

Sample Name	:
Tray#	: 1
Vial#	: 10
Injection Volume	: 1
Data File	: HI-7m.lcd
Method File	: MSP5-95_30min_1,0.lcm
Batch File	: B_010725.lcb
Report Format File	: Reportformat2.lsr
Date Acquired	: 01.07.2025 11:55:08
Date Processed	: 01.07.2025 12:25:11
Comment	: MeOH/H2O/0,05%TFA

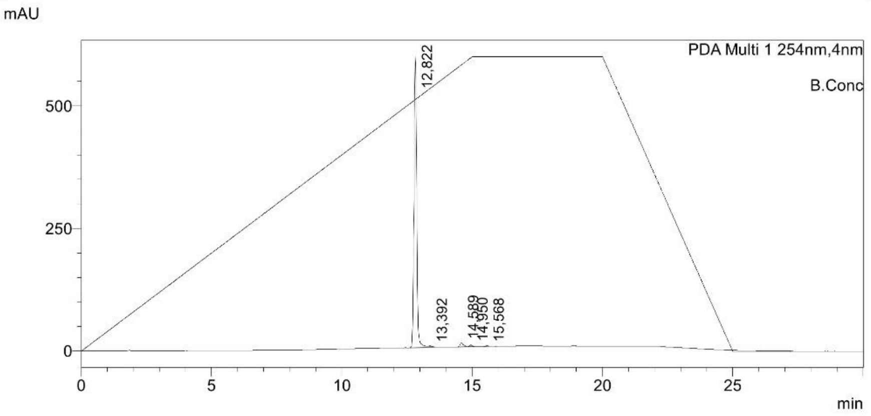

PDA Ch1 254nm

Peak#	Ret. Time	Area	Height	Area%
1	12,822	4370046	593371	97,217
2	13,392	8274	1361	0,184
3	14,589	75577	8178	1,681
4	14,950	30394	3392	0,676
5	15,568	10839	1649	0,241
Total		4495131	607952	100,000

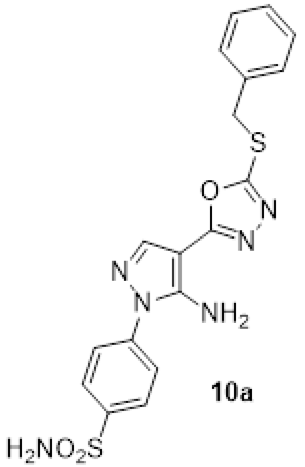

10a

D:\HPLC\Hani Ibrahim\HI-7m.lcd

Figure S51. HPLC chromatogram of compound **10a**

Analysenreport

Sample Information

Sample Name	:
Tray#	: 1
Vial#	: 21
Injection Volume	: 10
Data File	: HI-8m_P1_F16.lcd
Method File	: MSP5-95_30min_1.0_96well.lcm
Batch File	: B_181225-1.lcb
Report Format File	: Reportformat2.lsr
Date Acquired	: 18.12.2025 15:42:49
Date Processed	: 18.12.2025 16:12:50
Comment	: MeOH/H2O/0,05%TFA

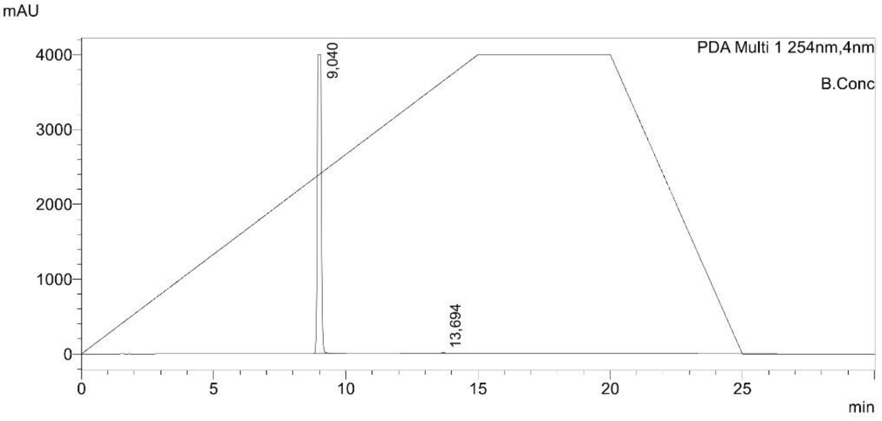

PDA Ch1 254nm

Peak#	Ret. Time	Area	Height	Area%
1	9,040	40173352	3995969	99,669
2	13,694	133384	15324	0,331
Total		40306736	4011293	100,000

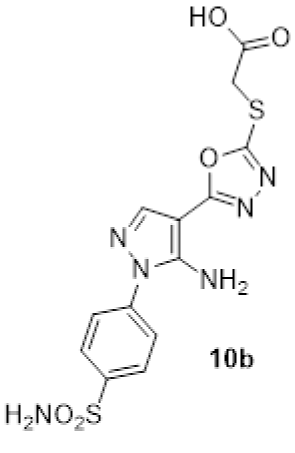

10b

D:\HPLC\Hani Ibrahim\HI-8m_P1_F16.lcd

Figure S52. HPLC chromatogram of compound **10b**

Analysenreport

Sample Information

Sample Name	:
Tray#	: 2
Vial#	: 27
Injection Volume	: 10
Data File	: HI-9m_P1_F47.lcd
Method File	: MSP5-95_30min_1.0_96well.lcm
Batch File	: B_181225-1.lcb
Report Format File	: Reportformat2.lsr
Date Acquired	: 19.12.2025 06:59:09
Date Processed	: 19.12.2025 07:29:12
Comment	: MeOH/H2O/0,05%TFA

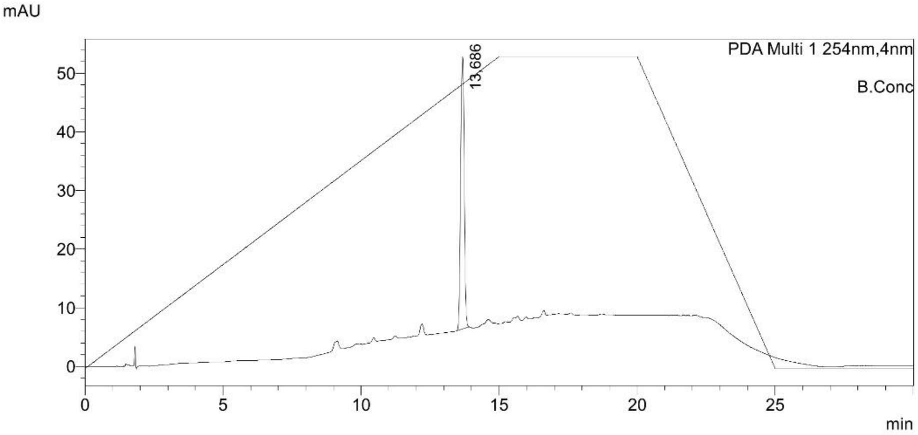

PDA Ch1 254nm

Peak#	Ret. Time	Area	Height	Area%
1	13,686	431991	46411	100,000
Total		431991	46411	100,000

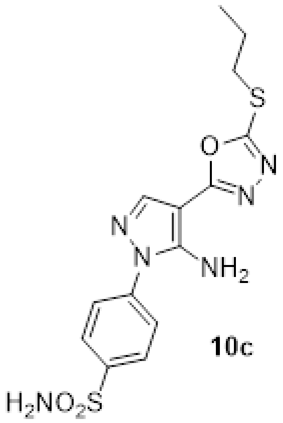

10c

D:\HPLC\Hani Ibrahim\HI-9m_P1_F47.lcd

Figure S53. HPLC chromatogram of compound **10c**

Analysenreport

Sample Information

Sample Name	:
Tray#	: 1
Vial#	: 13
Injection Volume	: 1
Data File	: HI-10m.lcd
Method File	: MSP5-95_30min_1,0.lcm
Batch File	: B_010725.lcb
Report Format File	: Reportformat2.lsr
Date Acquired	: 01.07.2025 13:26:44
Date Processed	: 01.07.2025 13:56:47
Comment	: MeOH/H2O/0,05%TFA

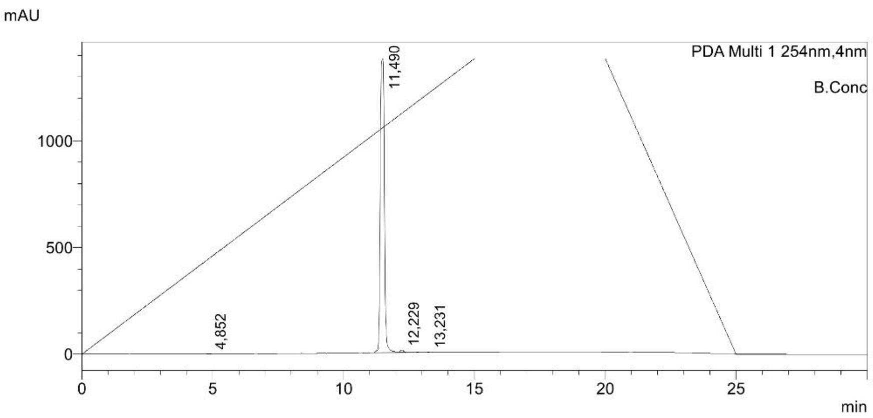

PDA Ch1 254nm

Peak#	Ret. Time	Area	Height	Area%
1	4,852	23303	2029	0,154
2	11,490	15039060	1378645	99,344
3	12,229	69789	9407	0,461
4	13,231	6157	956	0,041
Total		15138308	1391037	100,000

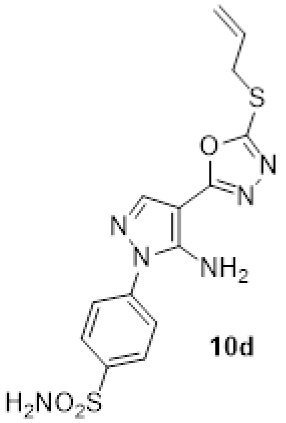

10d

D:\HPLC\Hani Ibrahim\HI-10m.lcd

Figure S54. HPLC chromatogram of compound **10d**

Analysenreport

Sample Information

Sample Name	:
Tray#	: 2
Vial#	: 20
Injection Volume	: 10
Data File	: HI-12m_P1_F40.lcd
Method File	: MSP5-95_30min_1.0_96well.lcm
Batch File	: B_181225-1.lcb
Report Format File	: Reportformat2.lsr
Date Acquired	: 19.12.2025 03:25:17
Date Processed	: 19.12.2025 03:55:22
Comment	: MeOH/H2O/0,05%TFA

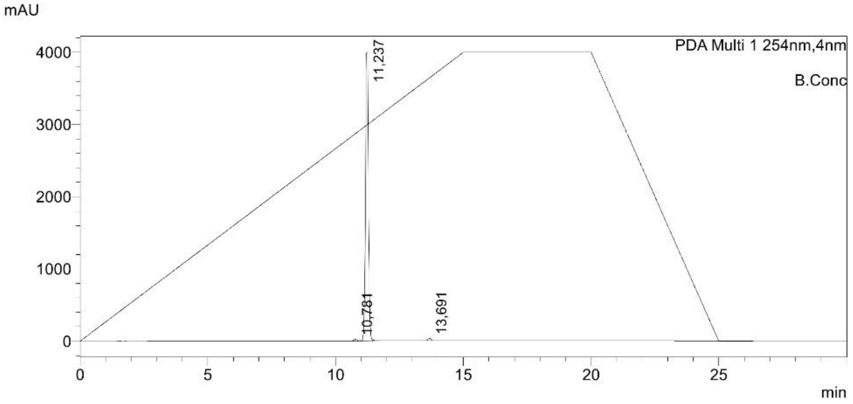

PDA Ch1 254nm

Peak#	Ret. Time	Area	Height	Area%
1	10,781	199656	22801	0,618
2	11,237	31783489	3993901	98,457
3	13,691	298418	32243	0,924
Total		32281563	4048945	100,000

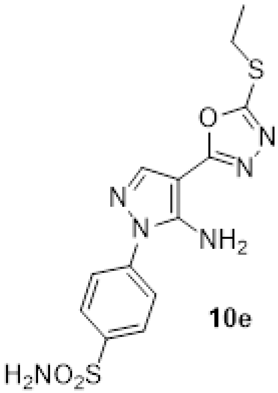

10e

D:\HPLC\Hani Ibrahim\HI-12m_P1_F40.lcd

Figure S55. HPLC chromatogram of compound **10e**

4. X-ray crystallographic analysis

X-RAY CRYSTALLOGRAPHIC ANALYSIS

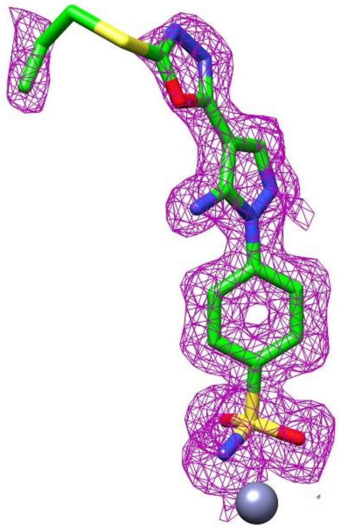

Figure S56. Electron density of inhibitor **10d** (green, PDB: 9T4S) bound to zinc (grey) in hCA II active site. $2F_o$-F_c maps and contoured to the 1.0 σ level.

Table S1. Summary of Data Collection and Atomic Model Refinement Statistics for hCAII

	**hCAII + 10M**
PDB ID	9T4S
Wavelength (Å)	0.87313
Space Group	P21
Unit cell (a, b, c, α, β, γ)(Å,°)	42.29, 41.33, 71.93<br>90.00, 104.38, 90.00
Limiting resolution (Å)	1.32-40.97 (1.32-1.34)
Unique reflections	53263 (2254)
Rmerge (%)	3.3 (20.8)
Rmeas (%)	5.2 (41.6)
Redundancy	2.1 (1.9)
Completeness overall(%)	94.5 (81.3)
$\langle I/\sigma(I) \rangle$	13.1 (2.7)
CC (1/2)	0.999 (0.801)
**Refinement statistics**	
Resolution range(Å)	1.32-40.97
Rfactor (%)	12.50
Rfree(%)	16.04
r.m.s.d. bonds(Å)	0.0118
r.m.s.d. angles (°)	1.9299
**Ramachandran statistics** (%)	
Most favored	96.9
additionally allowed	3.1
outlier regions	0.0
**Average B factor (Å^2)**	
All atoms	15.920
inhibitor	23.124
solvent	27.615

5. Biological evaluation

COX-1 IC_{50} Estimation Using BioVision Kit (K548-100)

This protocol is specifically adapted for the **BioVision COX-1 Inhibitor Screening Kit (Fluorometric)**, Catalog #K548-100 (revision 06/20). This kit is also sold as Abcam #ab204698 following BioVision's acquisition by Abcam in 2021 . The protocol incorporates your specified compound concentrations: **100, 10, 1, 0.1, and 0.01 µg/mL**.

I. Experimental Design and Plate Layout

Before starting, plan your 96-well plate layout. Each concentration should be tested in **triplicate (n=3)** for statistical validity. A recommended layout:

Row	Column 1	Column 2	Column 3	Column 4	Column 5	Column 6
A	Blank	Blank	EC	EC	IC	IC
B	100 µg/mL	100 µg/mL	100 µg/mL	0.1 µg/mL	0.1 µg/mL	0.1 µg/mL
C	10 µg/mL	10 µg/mL	10 µg/mL	0.01 µg/mL	0.01 µg/mL	0.01 µg/mL
D	1 µg/mL	1 µg/mL	1 µg/mL			

Required Controls:

- **Blank:** No enzyme, no inhibitor (for background subtraction)
- **Enzyme Control (EC):** Enzyme + solvent without inhibitor (100% activity)
- **Inhibitor Control (IC):** Enzyme + SC560 (positive control, provided with kit)

II. Reagent Preparation (Critical Steps)

⚠ **Important:** Briefly spin all small vials before opening to collect contents at the bottom. Keep COX-1 enzyme on ice at all times during use, but avoid prolonged ice storage .

A. COX-1 Enzyme Reconstitution

Reconstitute the COX-1 vial with **110 µL of sterile ddH_2O**. Mix gently. Aliquot and store at **-80°C** for long-term use (2 months). For short-term (2 weeks), store at -20°C .

B. Arachidonic Acid Substrate (Prepare fresh immediately before use)

1. Reconstitute the Arachidonic Acid vial with **55 µL of 100% Ethanol**. Vortex for 15-30 seconds.
2. Add **5 µL** of this solution to **5 µL of NaOH** (provided). Vortex briefly.
3. Dilute this mixture **10-fold** by adding **90 µL ddH_2O**. Vortex. **Use within 1 hour on ice** .

C. Diluted COX Cofactor (Prepare fresh immediately before use)

Dilute COX Cofactor **200-fold** by adding **2 µL** of COX Cofactor to **398 µL** of COX Assay Buffer. Mix well. **Stable for 1 hour at room temperature** - do not store .

III. Test Compound Serial Dilution

Prepare your compound at **10X the desired final concentration** in COX Assay Buffer. Since 10 µL of diluted compound will be added to a 100 µL total reaction volume, prepare solutions at:

Desired Final Conc. (µg/mL)	Prepare 10X Stock (µg/mL)	Dilution Instructions
100	1000	Highest concentration stock
10	100	1:10 dilution of 1000 µg/mL
1	10	1:10 dilution of 100 µg/mL
0.1	1	1:10 dilution of 10 µg/mL
0.01	0.1	1:10 dilution of 1 µg/mL

Solvent Note: If your compound is dissolved in DMSO, ensure the final DMSO concentration is consistent across all wells (typically ≤1%). Prepare a **Solvent Control** if DMSO effects are a concern .

IV. Reaction Setup

A. Master Mix Preparation (for 20 wells, prepare ~10% excess)

For each well, prepare Reaction Master Mix as follows :

Component	Volume per Well (µL)	Volume for 20 Wells (µL)
COX Assay Buffer	76	1520
COX Probe	1	20
Diluted COX Cofactor	2	40
COX-1 Enzyme	1	20
**Total**	**80**	**1600**

Note for Blank Wells: Replace COX-1 Enzyme with 1 µL COX Assay Buffer.

B. 96-Well Plate Loading

1. Add **10 µL** of each diluted test compound into assigned wells (triplicate).
2. Add **10 µL** COX Assay Buffer to Enzyme Control (EC) wells.

3. For Inhibitor Control (IC): Add **2 µL SC560 + 8 µL COX Assay Buffer** (SC560 provided with kit at unknown concentration - follow kit datasheet for final concentration).
4. Add **80 µL** Reaction Master Mix to each well (except blanks - use master mix without enzyme).

C. Reaction Initiation

Critical timing: Pre-set your plate reader before initiating reactions.

Using a multichannel pipette, quickly add **10 µL** of diluted Arachidonic Acid/NaOH solution to each well to start all reactions simultaneously .

Final well volume = 100 µL (10 µL compound + 80 µL master mix + 10 µL arachidonic acid)

V. Fluorescence Measurement

Instrument Settings

- **Excitation:** 535 nm
- **Emission:** 587 nm
- **Mode:** Kinetic
- **Temperature:** 25°C
- **Duration:** 10-30 minutes
- **Read interval:** Every 15-30 seconds

Data Collection

Record fluorescence values (RFU - Relative Fluorescence Units) at multiple time points. The reaction should show a linear increase in fluorescence over time.

VI. Data Analysis for IC_{50} Calculation

A. Calculate Reaction Slopes

For each well, select two time points (T_1 and T_2) within the **linear range** of the kinetic curve :

$\Delta RFU = RFU(T_2) - RFU(T_1)$

Slope = $\Delta RFU / \Delta T$ (where $\Delta T = T_2 - T_1$ in minutes)

B. Calculate Percent Inhibition

For each compound concentration, average the slopes of triplicate wells :

Inhibition % = [(Slope_EC – Slope_Sample) / Slope_EC] × 100

Where:

- **Slope_EC** = Average slope of Enzyme Control (no inhibitor)
- **Slope_Sample** = Average slope of compound-treated well

C. IC50 Determination

1. Plot **Inhibition (%)** on the Y-axis against **Log_{10}(Concentration)** on the X-axis (in µg/mL)
2. Perform **non-linear regression** using a four-parameter logistic (4PL) model:

Y = Bottom + (Top – Bottom) / (1 + 10^((LogIC50 – X) × HillSlope))

3. Most analysis software can perform this calculation:
   - **GraphPad Prism:** "Log(inhibitor) vs. response -- Variable slope"
   - **Microsoft Excel:** Use Solver add-in or online IC50 calculators
   - **R/Python:** Use `drm()` from `drc` package or `scipy.optimize.curve_fit`

D. Quality Control Checks

Parameter	Acceptance Criteria
Enzyme Control (EC) slope	Positive linear response ($R^2 > 0.95$)
SC560 (IC) inhibition	Should match kit expected IC50 (~6.45 nM for SC560)
Blank wells	Fluorescence near baseline
Triplicate CV	< 15% at each concentration

VII. Expected Results and Interpretation

Your final IC50 value is the concentration at which 50% of COX-1 enzyme activity is inhibited.

Typical IC50 ranges:

- **Strong inhibitor:** < 1 µg/mL
- **Moderate inhibitor:** 1-50 µg/mL
- **Weak inhibitor:** > 50 µg/mL

For comparison, the kit's positive control **SC560** typically shows IC50 ≈ 6.45 nM (approximately 0.002 µg/mL for this compound) .

VIII. Troubleshooting Guide

Problem	Possible Cause	Solution
Low fluorescence signal	Enzyme inactive or degraded	Use fresh enzyme aliquot; keep on ice
High background	Contamination	Check pipetting technique; use fresh reagents
Non-linear kinetics	Substrate depleted	Shorter measurement time; verify arachidonic acid preparation
Poor reproducibility	Inconsistent reaction start	Use multichannel pipette; pre-program plate reader
DMSO inhibition >10%	Solvent effect	Include solvent control; reduce final DMSO to ≤0.5%

IX. Notes and References

- This kit is also sold as **Abcam #ab204698** - both catalog numbers refer to the same product
- The assay detects Prostaglandin G_2, the intermediate product generated by COX-1 enzyme activity
- Ovine COX-1 is provided in this kit
- For COX-2 specific screening, use BioVision Kit #K547-100 or Abcam #ab283401

Storage: Store all components at -20°C, protected from light

COX-2 IC_{50} Estimation

COX-2 Inhibitor Screening Kit (Fluorometric) (Catalog #K547-100), here is a detailed procedure for estimating the IC50 of your compound using the requested serial dilution of **100, 10, 1, 0.1, and 0.01 µg/mL**.

This protocol combines official kit instructions with standard practices for inhibitor screening.

A. Protocol Overview

The assay measures the ability of your compound to inhibit the COX-2 enzyme. The kit includes a fluorescent probe that detects the enzymatic product. In the presence of an inhibitor, the fluorescent signal decreases. The IC50 is the concentration of your compound that reduces this signal by 50%.

B. Reagent Preparation (From Kit Components)

Before starting, prepare the following kit components as described. All components should be stored at -20°C and protected from light unless otherwise specified.

- **COX-2 Enzyme:** Briefly spin the vial to collect contents at the bottom. Reconstitute with **110 µL of sterile deionized water (ddH$_2$O)**. Mix gently. Aliquot into single-use tubes and store at -80°C. Keep on ice while in use and discard any leftover enzyme after use.
- **Arachidonic Acid (Substrate):** Briefly spin the vial. Reconstitute with **55 µL of 100% Ethanol**. Vortex for 15-30 seconds to ensure complete dissolution.
- **COX Cofactor (Diluted):** Immediately before use, dilute the COX Cofactor 200-fold. For example, add **2 µL of COX Cofactor** to **398 µL of COX Assay Buffer**. Mix well. This diluted solution is unstable, so prepare fresh for each experiment.
- **Arachidonic Acid/NaOH Working Solution (Diluted):** This activates the substrate. Prepare immediately before use.
    1. Add **5 µL of the reconstituted Arachidonic Acid** to **5 µL of the supplied NaOH**. Vortex briefly.
    2. Add **90 µL of ddH$_2$O** to this mixture and vortex. This is your 10X working solution. Keep it on ice.

C. Compound Preparation (Serial Dilution)

You will prepare your compound at **10X the final desired test concentration** because 10 µL of it will be added to a 100 µL total reaction volume.

1. **Solvent:** Dissolve your compound in an appropriate solvent (typically DMSO or ethanol) at a high concentration.
2. **10X Stock Solutions:** Prepare a 5-point serial dilution in **COX Assay Buffer** at 10X the concentrations listed below.

Final Conc. in Well (μg/mL)	Required 10X Stock Conc. (μg/mL)
100	1000
10	100
1	10
0.1	1
0.01	0.1

- **Note:** If your compound is dissolved in a solvent like DMSO, ensure the final concentration of the solvent in the assay well (e.g., 0.1-1% v/v) does not interfere with the enzyme. Run a **solvent control** (wells with the same amount of solvent but no compound) if this is a concern.

D. Plate Layout Design

Design your 96-well plate to include the following in triplicate for statistical reliability:

- **Test Wells (S):** Your compound at each of the 5 concentrations.
- **Enzyme Control (EC):** No inhibitor. This represents 100% enzyme activity. Use 10 μL of COX Assay Buffer.
- **Inhibitor Control (IC):** Use the kit's positive control, **Celecoxib**. The kit provides a 100 μL stock. According to the protocol, add **2 μL of Celecoxib** to **8 μL of COX Assay Buffer** for the Inhibitor Control well.
- **Blank:** Use Assay Buffer to correct for background fluorescence (no enzyme).

E. Step-by-Step Assay Protocol

1. **Add Compounds to Plate:** To your assigned wells, add **10 μL** of the appropriate solution:
    - **Test Wells (S):** 10 μL of each 10X test compound stock.
    - **Enzyme Control (EC):** 10 μL of COX Assay Buffer.
    - **Inhibitor Control (IC):** 10 μL of the prepared Celecoxib solution.
    - **Blanks:** 10 μL of COX Assay Buffer.
2. **Prepare the Reaction Master Mix:** For **each well** (calculate for your total number of wells + 10% excess), combine the following components in a tube on ice:

Component	Volume per Well
COX Assay Buffer	76 μL
COX Probe	1 μL
Diluted COX Cofactor (from Step B.3)	2 μL
COX-2 Enzyme (reconstituted from Step B.1)	1 μL

- **Note:** For the **Blank wells**, replace the 1 μL of COX-2 Enzyme with 1 μL of COX Assay Buffer in the master mix.

3. **Start the Reaction:** Add **80 µL** of the appropriate Reaction Master Mix to each well. Gently tap the plate to mix.
4. **Initiate with Substrate:** Use a multichannel pipette to quickly add **10 µL** of the **Diluted Arachidonic Acid/NaOH Working Solution** (from Step B.4) to all wells to start the reaction simultaneously.
5. **Measure Fluorescence:** Immediately place the plate in a fluorescence microplate reader. Measure the kinetics at **Ex/Em = 535/587 nm** at 25°C for 5-10 minutes.

F. Data Analysis for IC50 Calculation

1. **Calculate the Rate (ΔRFU/min):** For each well, determine the change in Relative Fluorescence Units (RFU) over the linear range of the reaction (e.g., between 1 and 5 minutes).
2. **Correct for Background:** Subtract the Blank value (rate) from all Test, EC, and IC well values to get the corrected rate.
3. **Calculate Percent Inhibition:** Use the following formula:

   ```
   % Inhibition = [(Rate of EC - Rate of S) / Rate of EC] × 100
   ```

   - **Rate of EC:** Corrected rate of the Enzyme Control (no inhibitor).
   - **Rate of S:** Corrected rate of your Test sample at a given concentration.
4. **Plot and Determine IC50:**
   - Plot the **Log of the final compound concentration (µg/mL)** on the X-axis (e.g., 100, 10, 1, 0.1, 0.01).
   - Plot the **% Inhibition** on the Y-axis.
   - Perform a non-linear regression analysis using software (e.g., GraphPad Prism, Origin Pro) with a **log(inhibitor) vs. response -- Variable slope (four-parameters)** equation. The software will calculate the IC50 value for you.

Summary of Critical Steps

- **Enzyme Handling:** The COX-2 enzyme is sensitive. Keep it on ice only for short periods (<30 min) and avoid freeze-thaw cycles.
- **Timing:** The Arachidonic Acid/NaOH and Diluted Cofactor solutions must be prepared **immediately before** the assay as they are unstable.
- **Replicates:** Always run samples in **triplicate or duplicate** to ensure your IC50 calculation is accurate.
- **Positive Control:** The kit includes **Celecoxib**, a known COX-2 inhibitor. Running this control validates your assay.

A. *In vitro* COX-1 inhibitory Activity

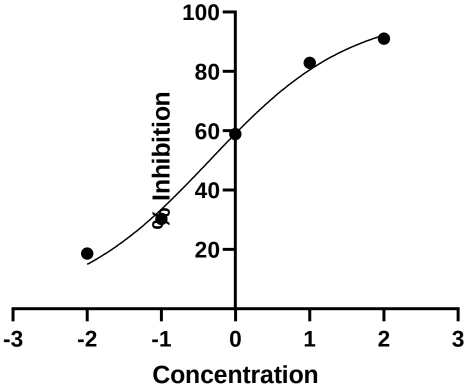

(5a)

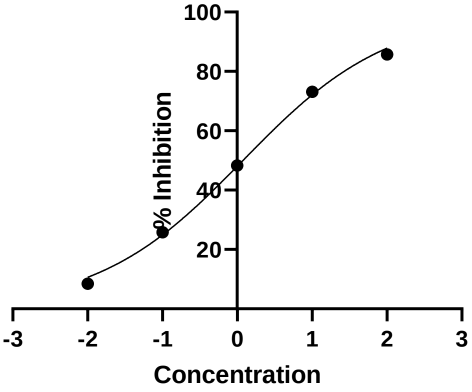

(5b)

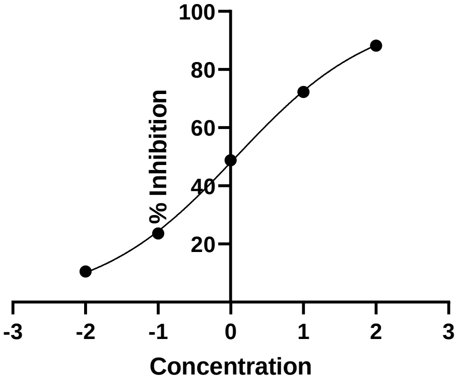

(5c)

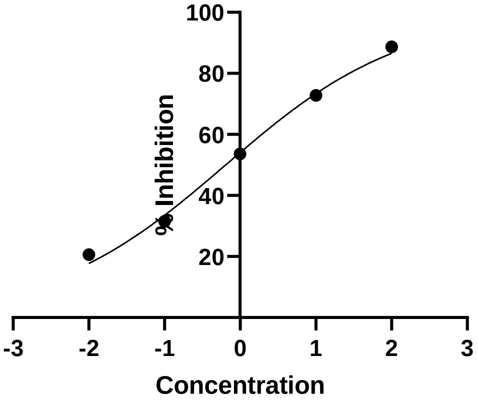

(5d)

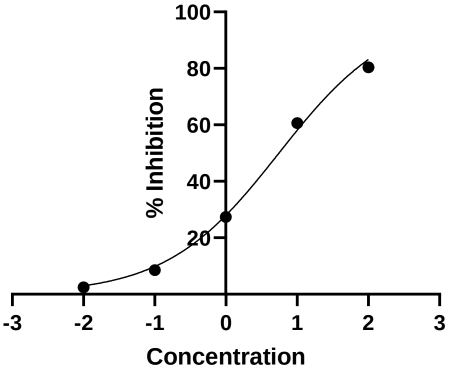

(5e)

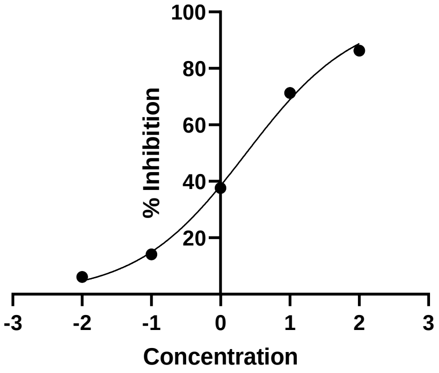

(5f)

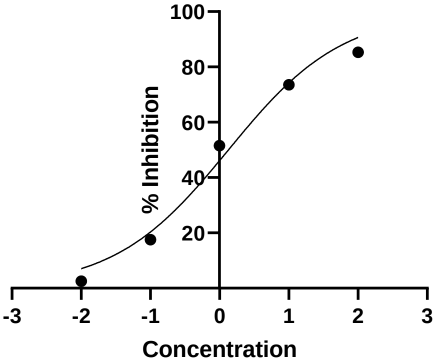

(5g)

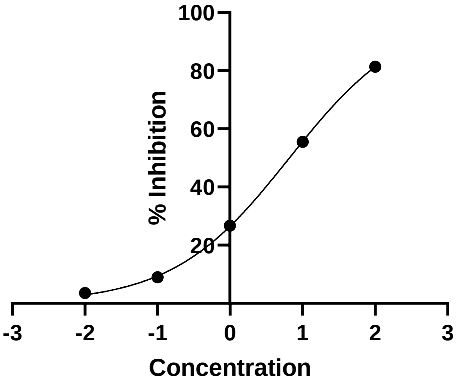

(10a)

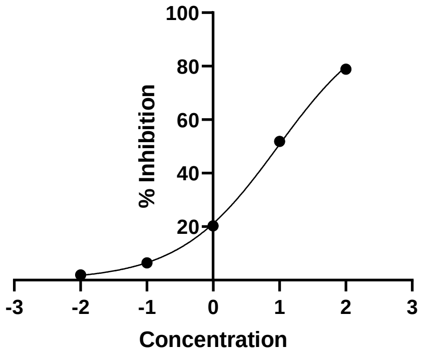

(10b)

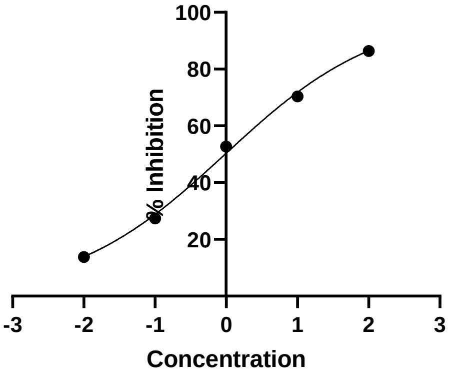

(10c)

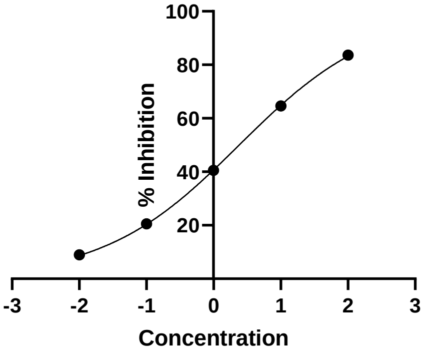

(10d)

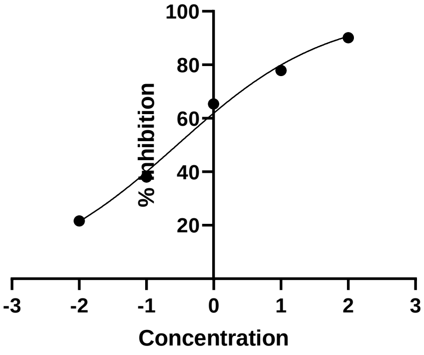

(10e)

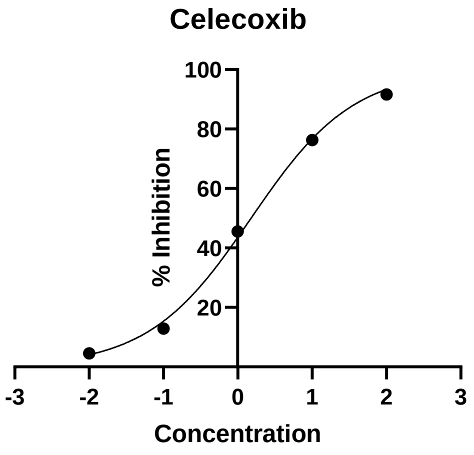

Celecoxib

Figure S57. *In vitro* COX-1 inhibitory Activity of target compounds and celecoxib standard

B. *In vitro* COX-2 inhibitory Activity

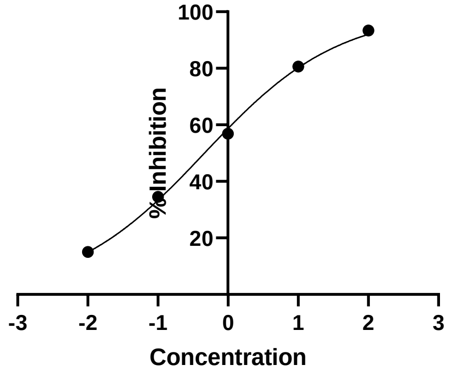

(5a)

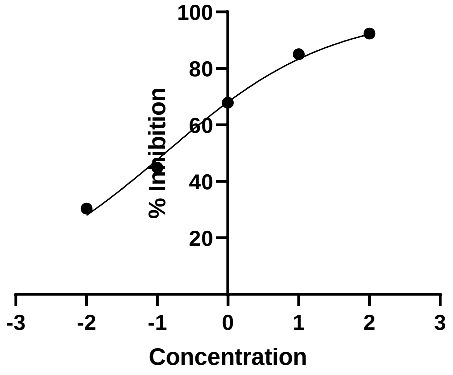

(5b)

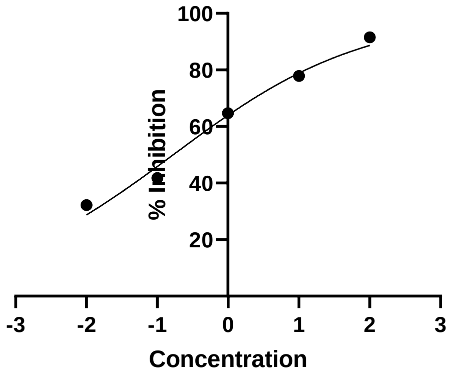

(5c)

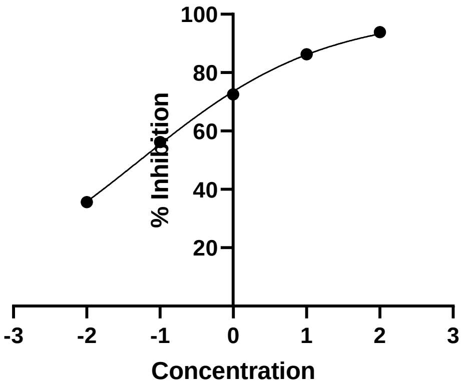

(5d)

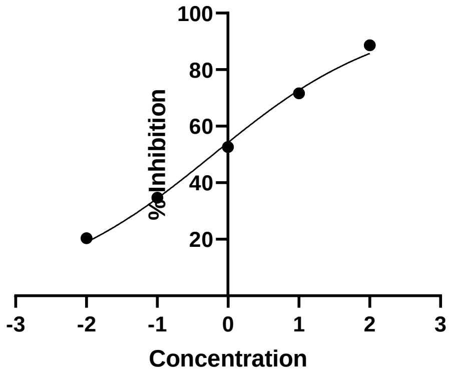

(5e)

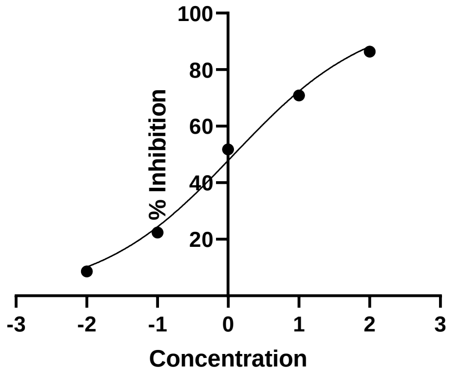

(5f)

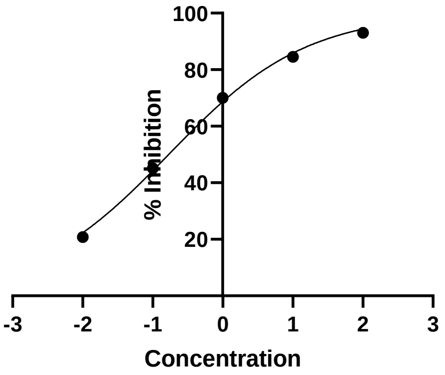

(5g)

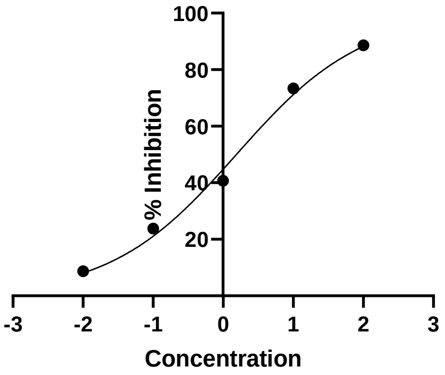

(10a)

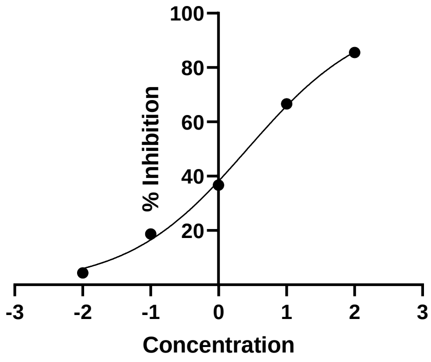

(10b)

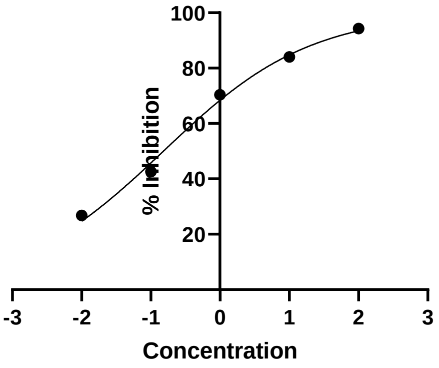

(10c)

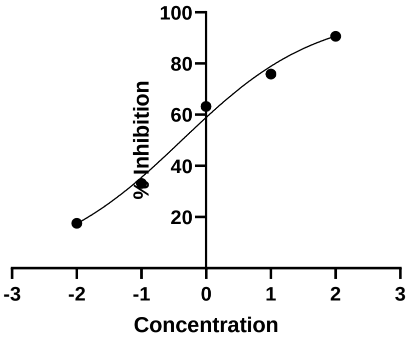

(10d)

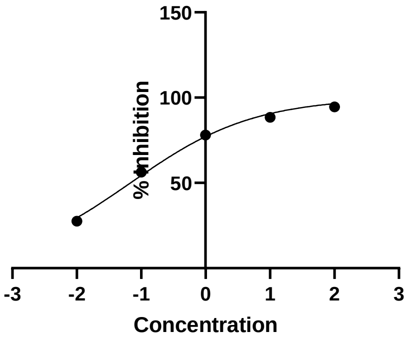

(10e)

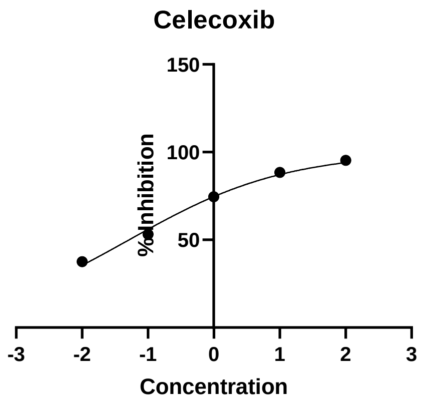

celecoxib

Figure S58. *In vitro* COX-2 inhibitory Activity of target compounds and celecoxib standard

C. *In vitro* CA inhibitory Activity

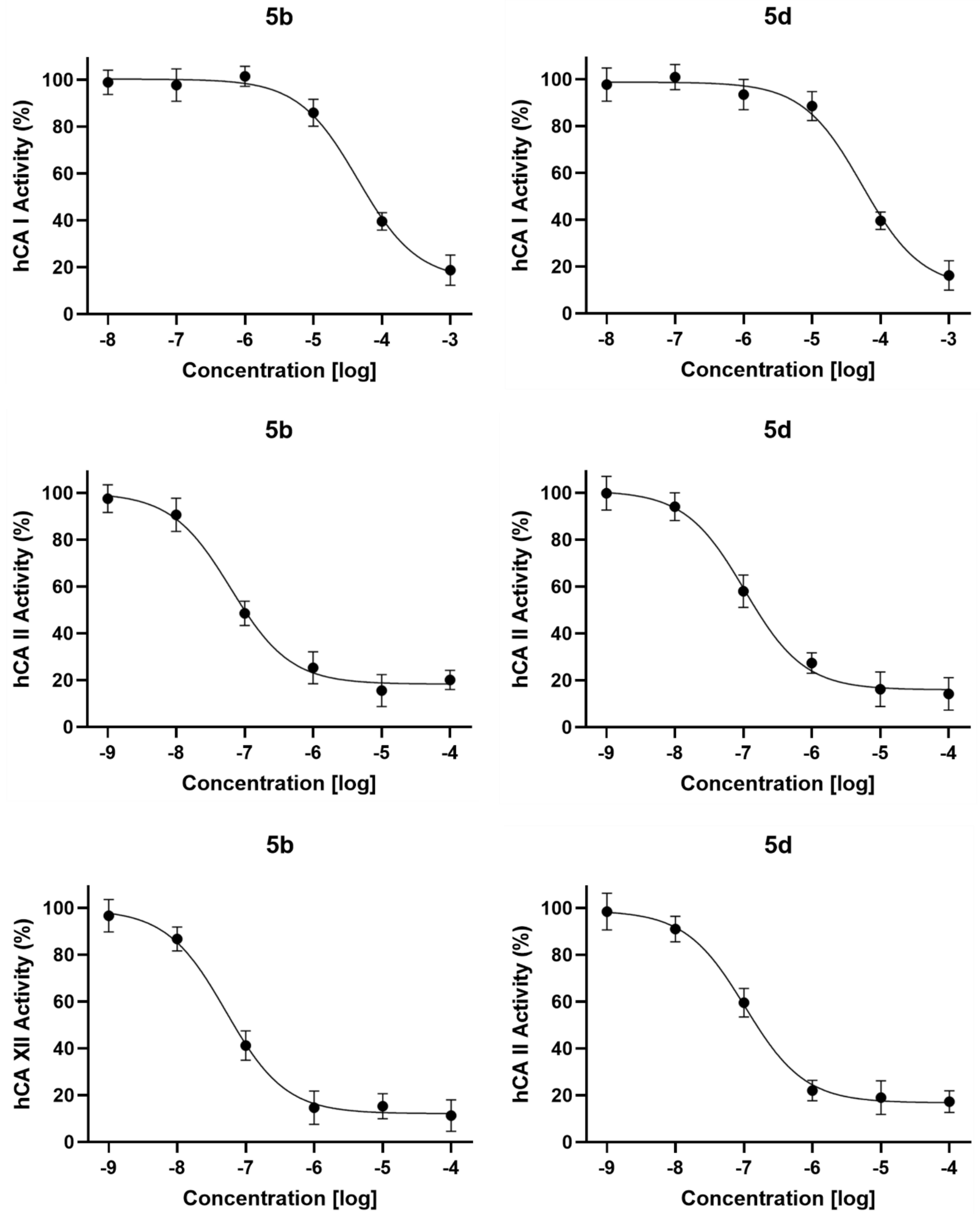

Figure S59. Inhibition plots of the most representative compounds 5b and 5d against hCA I, II, and XII.

In vivo animal studies

A- Evaluation of analgesic activity

Animals

Male albino mice weighing 30–40 g were used for the evaluation of analgesic activity. Animals were housed under controlled environmental conditions (24 ± 2 °C, 12 h light/dark cycle) with free access to standard diet and water, and were acclimatized by daily handling for 3 min over a period of four weeks prior to the experiment. All experimental procedures were conducted in accordance with the approved ethical guidelines for animal care and use. Animals were randomly divided into four groups (n = 3 per group). Test groups received intraperitoneal (i.p.) injections of compounds 5b or 5d at a dose of 50 mg/kg, while the control group received normal saline (i.p.). A separate standard group received Ibuprufen as a reference analgesic drug at a dose of 25 mg/kg (i.p.). Analgesic activity was assessed using the hot-plate test, in which animals were individually placed on a heated surface maintained at 40 °C. Antinociceptive activity was determined by recording the latency time to hind paw licking or jumping response. Baseline latency was measured before treatment, and animals exhibiting abnormal baseline responses were excluded from the study. To avoid tissue injury, a cutoff latency time was applied throughout the experiment. Latency measurements were recorded at 10, 20, 30, 40, 50, 60, 70, 80, and 90 min following drug administration.

Statistical Analysis.

All data are presented as the mean ± standard error of the mean (SEM) for n = 3 independent replicates. Statistical evaluations were performed using GraphPad Prism software (Version 8.0). For the analgesic activity time-course, data were analyzed using a two-way repeated-measures (RM) ANOVA followed by Tukey's post-hoc test for multiple comparisons to evaluate the simple effects of treatment within each time point. Sphericity was not assumed, and the Geisser–Greenhouse correction was applied where appropriate. A *p*-value of less than 0.05 was considered statistically significant.

Table S2. Detailed statistical analysis for the analgesic study.

Time (min)	Tukey's multiple comparisons test	Mean Difference	95% Confidence Interval (Cl)	Exact *P* value
10	Normal vs. Ibuprufen	-5.7	-7.096 to -4.304	<.001
	Normal vs. 5b	-1.7	-3.149 to -0.2512	0.029
	Normal vs. 5d	-4.7	-6.035 to -3.365	0.001

20	Normal vs. Ibuprufen	-7	-8.000 to -6.000	<.001
	Normal vs. 5b	-2	-2.616 to -1.384	0.004
	Normal vs. 5d	-5	-6.705 to -3.295	0.006
30	Normal vs. Ibuprufen	-6	-6.836 to -5.164	<.001
	Normal vs. 5b	-3	-3.609 to -2.391	<.001
	Normal vs. 5d	-4	-4.836 to -3.164	<.001
40	Normal vs. Ibuprufen	-7	-7.809 to -6.191	<.001
	Normal vs. 5b	-5	-5.628 to -4.372	<.001
	Normal vs. 5d	-7	-8.263 to -5.737	<.001
50	Normal vs. Ibuprufen	-7	-7.530 to -6.470	<.001
	Normal vs. 5b	-6	-7.221 to -4.779	<.001
	Normal vs. 5d	-5	-5.609 to -4.391	<.001
60	Normal vs. Ibuprufen	-9.333	-11.97 to -6.695	0.004
	Normal vs. 5b	-4	-4.316 to -3.684	<.001
	Normal vs. 5d	-6	-7.705 to -4.295	0.004
70	Normal vs. Ibuprufen	-6	-7.703 to -4.297	0.003
	Normal vs. 5b	-4	-4.534 to -3.466	<.001
	Normal vs. 5d	-6	-6.890 to -5.110	<.001
80	Normal vs. Ibuprufen	-6	-7.385 to -4.615	0.002
	Normal vs. 5b	-3	-3.405 to -2.595	<.001
	Normal vs. 5d	-3	-4.221 to -1.779	0.006
90	Normal vs. Ibuprufen	-8	-8.534 to -7.466	<.001
	Normal vs. 5b	-6	-7.458 to -4.542	0.002
	Normal vs. 5d	-5	-6.037 to -3.963	<.001

In vivo animal studies

B- Evaluation of anti-inflammatory activity

Animals

Thirty adult male albino rats, weighing 170–200 g were obtained from Nahda University Animal House, Beni-Sueif, Egypt. The animals were housed in a room with controlled temperature (22 ± 1 °C), humidity (60%), and illumination (12 hours of light and dark). This allowed the animals to acclimate. Water and food were available at all times during the trial. Rats were kept in opaque, well-ventilated cages made of propylene, with free access to regular feed. Handling of animals and animal care were performed in accordance with the guidelines.

Experimental design

After one week adaptation, healthy rats were randomly divided into five groups, each of 3 rats as follow: Group 1 (control group); Group II (ibuprofen group); Group III (carrageenan group); Group IV (compound **5b** group); Group V (compound **5d** group).

Each of ibuprofen (25mg/kg), carrageenan (0.1 mL of 1% carrageenan for each rat), compound **5b** (50mg/kg), and compound **5d** (50mg/kg) were suspended in 2% Tween 80 and administrated orally 30 minutes before induction of the inflammation, while the control group received only 2% Tween 80, 30 minutes before induction of the inflammation.

Method

The right leg was subplantarly injected with 0.1 mL of 1% carrageenan solution in 0.9% saline to cause paw edema. Using a screw gauge micrometer, the thickness of the paw edema in the right hind paw was measured at 1, 2, 3, 4, 5, and 6 hours, and the results were compared to the left hind paw thickness of each rat. For every time interval, the average value difference between the treated and control groups was computed and statistically assessed. The normal distribution of quantitative data was represented by means ± standard error (SEM). The anti-inflammatory activity was expressed as percentage inhibition of edema volume in treated animals in comparison with the control group according to the following equation:

$$\% \text{ of edema inhibition} = \frac{(V_R - V_L)\text{ control} - (V_R - V_L)\text{ treated}}{(V_R - V_L)\text{ control}} \times 100$$

Where V_R represents the right paw thickness, V_L represents the left paw thickness, $(V_R - V_L)$ control represents the mean increase in paw thickness in the control group of rats and $(V_R - V_L)$ treated represents the mean increase in paw thickness in rats treated with the tested compounds.

Statistical Analysis. All data are expressed as the mean ± standard error of the mean (SEM) of 3 independent replicates (n = 3). Statistical evaluations were performed using GraphPad Prism software (Version 8.0). For the carrageenan-induced paw edema model, differences in paw volume over the 6-hour observation period were analyzed using a two-way repeated-measures analysis of variance (RM ANOVA). To evaluate the simple effects of treatments within each specific time point, Tukey's post-hoc multiple comparisons test was utilized. A *p*-value of less than 0.05 was considered statistically significant.

Table S3. Detailed statistical analysis for carrageenan-induced Paw Edema study.

**Time (h)**	**Comparison**	**Mean difference (Effect size in mL)**	**95% Confidence Interval (Cl)**	**Exact *P* value**
1	Carrageenan vs. Ibuprofen group	-0.09	-0.1473 to -0.03271	0.017
1	Carrageenan group vs. Compound 5b group	0.1	0.06526 to 0.1347	0.003
1	Carrageenan group vs. Compound 5d group	0.1013	0.06632 to 0.1364	0.003
2	Carrageenan vs. Ibuprofen group	0	-0.02974 to 0.02974	>.999
2	Carrageenan group vs. Compound 5b group	0.21	0.1932 to 0.2268	<.001
2	Carrageenan group vs. Compound 5d group	0.09	0.07663 to 0.1034	<.001
3	Carrageenan vs. Ibuprofen group	0.28	0.2563 to 0.3037	<.001
3	Carrageenan group vs. Compound 5b group	0.59	0.5567 to 0.6233	<.001
3	Carrageenan group vs. Compound 5d group	0.59	0.5625 to 0.6175	<.001
4	Carrageenan vs. Ibuprofen group	0.37	0.3627 to 0.3773	<.001
4	Carrageenan group vs. Compound 5b group	0.6787	0.6367 to 0.7206	<.001
4	carrageenan group vs. Compound 5d group	0.59	0.5717 to 0.6083	<.001
5	Carrageenan vs. Ibuprofen group	1.293	1.219 to 1.367	<.001
5	Carrageenan group vs. Compound 5b group	1.387	1.324 to 1.451	<.001
5	Carrageenan group vs. Compound 5d group	1.29	1.260 to 1.320	<.001
6	Carrageenan vs. Ibuprofen group	1.449	1.264 to 1.634	<.001
6	Carrageenan group vs. Compound 5b group	1.359	1.176 to 1.542	<.001
6	Carrageenan group vs. Compound 5d group	1.359	1.210 to 1.508	<.001

In vivo animal studies

C- Evaluation of ulcerogenic activity

Animals

We acquired adult male albino rats weighing between 170 and 200 g from the Nahda University Animal House located in Beni-Sueif, Egypt. The animals were housed in a room with controlled temperature (22 ± 1 °C), humidity (60%), and illumination (12 hours of light and dark). This allowed the animals to acclimate. Water and food were available at all times during the trial. Rats were kept in opaque, well-ventilated cages made of propylene, with free access to regular feed.

Experimental design

After one week of adaptation, four sets of experiments were performed. Each set contained thirty-six healthy rats that were randomly divided into four groups, each of 6 rats as follow: Group 1 (control group, 2% Tween 80+saline); Group II (ibuprofen group, 25mg/kg); Group III (Compound **5b**, 50mg/kg); and Group IV (Compound **5d**, 50mg/kg).

Method

Before the drugs were administered, all the rats were devoid of food for eighteen hours, but not of water. After that, 2% Tween 80 was used to suspend ibuprofen, Compound **5b**, and compound **5d** at the appropriate doses, which were then given orally to the animals. Animals in the first set of experiments were sacrificed by decapitation one hour after the drugs were administered; the stomachs were taken out, gathered, and opened along the larger curvature. They were then cleansed with distilled water and gently dipped in normal saline (0.9%).

Animal in the second and third set of experiment were sacrificed after 3 and 6 hours respectively, while animals in the fourth set were orally administered another two doses of drugs (once daily) for the following two days, then were sacrificed in the fourth day after 24 hours after the last dose of the drugs.

The mucosal damage of each stomach was examined with a magnifying lens for the presence of macroscopically visible lesions. The number of lesions in each stomach, if any, was counted and

recorded. The ulcerogenic effect was evaluated according to Meshali's method and ulcer index was calculated according to the method of Robert et al.

The degree of ulcerogenic effect was expressed in terms of:

i- Percentage of incidence of ulcers in each group of animals divided by 10.

ii- The average number of ulcers per stomach.

iii- The average severity of ulcers by visual observation: The ulcer scores were: 0 = no ulcer, 1 = mucosal erythema only, 2 = mild mucosal edema, slight bleeding or slight erosion, 3 = moderate edema, bleeding ulcers or erosions, 4 = sever ulceration, erosions, edema and tissue necrosis.

The ulcer index was expressed as summation value of above three values.

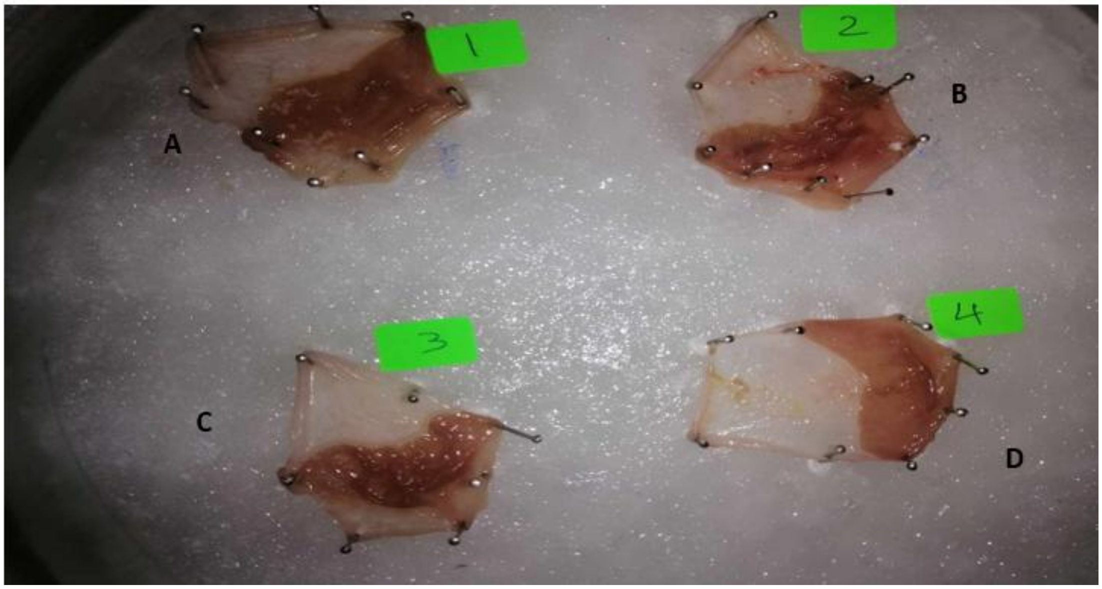

Figure S60. Ulcerogenic effect of A): control group; B): Ibuprofen group; C): Compound **5b** group; D): Compound **5d** group in rats after 24 hrs of their administration.

In vivo animal studies

D- Animal Glaucoma Model

For conducting the study, eight adult male New Zealand albino rabbits (weights ranged from 1900-2500 g) were obtained from the Medical Experimental Research Centre (MERC) of Mansoura University. Per the NIH Guidelines for the Care and Use of Laboratory Animals and strictly adherent to the ARRIVE guidelines (Animal Research: Reporting In Vivo Experiments), the study protocol was approved by the Research Ethics Committee of the Faculty of Pharmacy, Cairo University, Egypt (REC-FOPCU, approval no. PC3428). Animals were acclimatized in individual cages in a pathogen-free environment and controlled laboratory setting at a temperature range (22−24 °C) and light−dark cycle (12/12 h) for one week. Animals were allowed free access to drinking tap water and food. All animals were examined before the study to ensure good medical ophthalmic conditions and numbered sequentially. The IOP was measured using a Schiotz tonometer in both eyes of each animal at time zero (T_0) just before glaucoma induction (basal IOP ranged from 16.5-25.1 mmHg). For each rabbit, a glaucoma model was induced in both eyes by intravitreal injection of 0.05 mL of NaCl (5%). Ten minutes later, IOP was assessed in both eyes (IOP ranged from 81.4 to 109.3 mmHg). Then, rabbits were immediately and randomly divided into groups A and B (n=4) employing the Rand function in Excel (version 16.82). Topical administration of 0.05 ml of compound **5b** prepared as 1% solution and 0.1 ml of compound **5d** prepared as 0.05% solution into the conjunctival cul-de-sac of the right eye of each rabbit in the respective group with the instillation of the corresponding dose of the vehicle drops in the left eye, which was used as a control. IOP was measured bilaterally at 60, 90, 120, and 240 min after drug instillation. Each eye received one drop of oxybuprocaine hydrochloride (0.2%) just before IOP measurements.

Statistical Analysis Data are expressed as mean ± standard error of the mean (SEM), with a sample size of four rabbits per group (n = 4). An initial comprehensive assessment was conducted using a **three-way analysis of variance** (**ANOVA**) to examine the main effects and potential interactions among Time, Treatment (Treated vs. Sham), and Compound (**5b** vs. **5d**). To further characterize the pharmacodynamic behavior of each derivative while avoiding the statistical constraints associated with direct inter-compound comparisons, the dataset was

subsequently stratified by compound and analyzed using separate **two-way ANOVAs** for each individual derivative.

Using **Bonferroni's multiple comparison test**, post hoc comparisons between the treated (Rt) and contralateral sham (Lt) eyes at specific time intervals (0, 10, 60, 90, 120, and 240 minutes) were carried out. All statistical analyses and graphical representations were generated using GraphPad Prism software (version 8.0.2). A probability value (*P*) of less than 0.05 was regarded as indicative of statistical significance.

Table S4. Detailed statistical analysis for intraocular pressure assay.

Time (min)	Bonferroni's multiple comparisons test	Mean Difference	95% Confidence Interval (Cl)	Exact *P* Value
0	5b - Rt (Treated) vs. 5b - Lt (Sham)	0	-16.30 to 16.30	>0.9999
	5d - Rt (Treated) vs. 5d - Lt (Sham)	0.775	-11.82 to 13.37	>0.9999
10	5b - Rt (Treated) vs. 5b - Lt (Sham)	1.6	-14.70 to 17.90	>0.9999
	5d - Rt (Treated) vs. 5d - Lt (Sham)	3.85	-8.744 to 16.44	>0.9999
60	5b - Rt (Treated) vs. 5b - Lt (Sham)	-20.9	-37.20 to -4.597	0.0061
	5d - Rt (Treated) vs. 5d - Lt (Sham)	-5.85	-18.44 to 6.744	>0.9999
90	5b - Rt (Treated) vs. 5b - Lt (Sham)	-15.8	-32.10 to 0.5033	0.0621
	5d - Rt (Treated) vs. 5d - Lt (Sham)	-5.725	-18.32 to 6.869	>0.9999
120	5b - Rt (Treated) vs. 5b - Lt (Sham)	-12.45	-28.75 to 3.853	0.2394
	5d - Rt (Treated) vs. 5d - Lt (Sham)	-11.98	-24.57 to 0.6193	0.0705
240	5b - Rt (Treated) vs. 5b - Lt (Sham)	-11.43	-27.73 to 4.878	0.3492
	5d - Rt (Treated) vs. 5d - Lt (Sham)	-9.675	-22.27 to 2.919	0.2327

Table S5. Baseline body weights and randomization allocation scheme for rabbits in the exploratory IOP assay (*n* =4 per group).

	Group A	Group B
Rab. (1) 2.200 gram	☑	
Rab (2) 2.300 gram		☑
Rab. (3) 2.500 gram	☑	
Rab (4) 1.900 gram		☑
Rab (5) 2.200 gram		☑
Rab. (6) 2.250 gram	☑	
Rab (7) 2.200 gram		☑
Rab (8) 2.200 gram	☑	

Table S6. Individual raw IOP measurements for rabbits in group A over time.

**Group A [one drop of reference drug]**		
Rab. A(6) Wt. 2.250 gram	Rt eye (mmHg)	Lt eye sham (mmHg)
T0	19.6	19.6
After 10 min.	100.1	95.4
After 60 min.	34.4	46.9
After 90 min.	37.2	43.4
After 120 min.	37.2	43.4
After 240 min.	23.1	40.4
Rab. A(3) 2.500 gram	Rt eye (mmHg)	Lt eye sham (mmHg)
T0	16.5	16.5
After 10 min.	95.9	98
After 60 min.	37.2	69.3
After 90 min.	37.2	69.3
After 120 min.	37.2	59.1
After 240 min.	23.1	37.2
Rab. A(1) 2.200 gram	Rt eye (mmHg)	Lt eye sham (mmHg)
T0	23.1	23.1
After 10 min.	89.5	105.7
After 60 min.	29.4	50.6
After 90 min.	27.2	43.4
After 120 min.	27.2	40.2
After 240 min.	23.1	27.2
Rab A(8) 2.200 gram	Rt eye (mmHg)	Lt eye sham (mmHg)
T0	19.6	19.6

After 10 min.	101.4	81.4
After 60 min.	27	44.8
After 90 min.	23.1	31.8
After 120 min.	23.1	31.8
After 240 min.	19.6	29.8

Table S7. Mean and standard deviation (SD) of IOP measurements over time for rabbits in group A (*n*=4).

Group A [one drop of reference drug]						
Rt eye (mmHg)						
	T0	10 min	60 min	90 min	120 min	240 min
	19.6	100.1	34.4	37.2	37.2	23.1
	16.5	95.9	37.2	37.2	37.2	23.1
	23.1	89.5	29.4	27.2	27.2	23.1
	19.6	101.4	27	23.1	23.1	19.6
Mean	19.7	96.73	32	31.18	31.18	22.23
SD	2.70	5.36	4.64	7.16	7.16	1.75
Lt eye (mmHg)						
	T0	10 min	60 min	90 min	120 min	240 min
	19.6	95.4	46.9	43.4	43.4	40.4
	16.5	98	69.3	69.3	59.1	37.2
	23.1	105.7	50.6	43.4	40.2	27.2
	19.6	81.4	44.8	31.8	31.8	29.8
Mean	19.7	95.13	52.9	46.98	43.63	33.65
SD	2.70	10.14	11.19	15.86	11.42	6.18

Table S8. Individual raw IOP measurements for rabbits in group B over time.

Group B [two drops of reference drug]		
Rab B(2) 2.300 gram	Rt eye (mmHg)	Lt eye sham (mmHg)
T0	16.5	16.5
After 10 min.	107.4	88
After 60 min.	29.4	40.2
After 90 min.	27.2	37.2
After 120 min.	27.2	37.2
After 240 min.	19.6	24.4
Rab B(4) 1.900 gram	Rt eye (mmHg)	Lt eye sham (mmHg)
T0	19.6	16.5
After 10 min.	98.2	95.2
After 60 min.	37.2	37.2
After 90 min.	37.2	37.2

After 120 min.	23.1	39.4
After 240 min.	19.6	31.8
Rab B(5) 2.200 gram	Rt eye (mmHg)	Lt eye sham (mmHg)
T0	25.1	23.1
After 10 min.	109.3	109.3
After 60 min.	43.4	48.8
After 90 min.	37.2	43.4
After 120 min.	27.2	37.2
After 240 min.	19.6	27.2
Rab B(7) 2.200 gram	Rt eye (mmHg)	Lt eye sham (mmHg)
T0	23.1	25.1
After 10 min.	81.7	88.7
After 60 min.	43.4	50.6
After 90 min.	40.2	46.9
After 120 min.	31.8	43.4
After 240 min.	23.1	37.2

Table S9. Mean and standard deviation (SD) of IOP measurements over time for rabbits in group B (*n*=4).

**Group B [two drops of reference drug]**						
Rt eye (mmHg)						
	T0	10 min	60 min	90 min	120 min	240 min
	16.5	107.4	29.4	27.2	27.2	19.6
	19.6	98.2	37.2	37.2	23.1	19.6
	25.1	109.3	43.4	37.2	27.2	19.6
	23.1	81.7	43.4	40.2	31.8	23.1
Mean	21.08	99.15	38.35	35.45	27.33	20.48
SD	3.80	10.91	5.75	5.68	3.55	1.75
Lt eye (mmHg)						
	T0	10 min	60 min	90 min	120 min	240 min
	16.5	88	40.2	37.2	37.2	24.4
	16.5	95.2	37.2	37.2	39.4	31.8
	23.1	109.3	48.8	43.4	37.2	27.2
	25.1	88.7	50.6	46.9	43.4	37.2
Mean	20.3	95.3	44.2	41.18	39.3	30.15
SD	4.46	9.88	6.51	4.81	2.92	5.60

6. Molecular Modeling

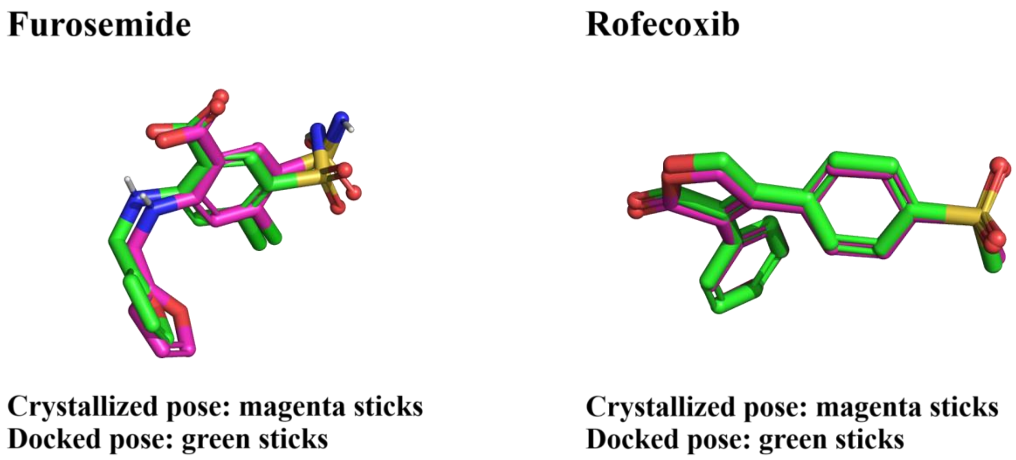

Figure S61. Validation of the docking protocol using furosemide (PDB 1Z9Y) and rofecoxib (PDB 5KIR)

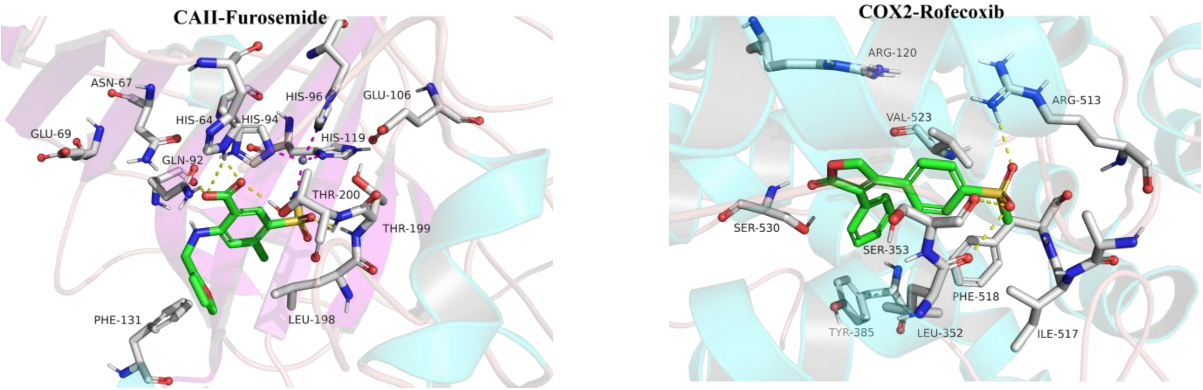

Figure S62. Docked poses of *h*CAII–furosemide and COX-2–rofecoxib

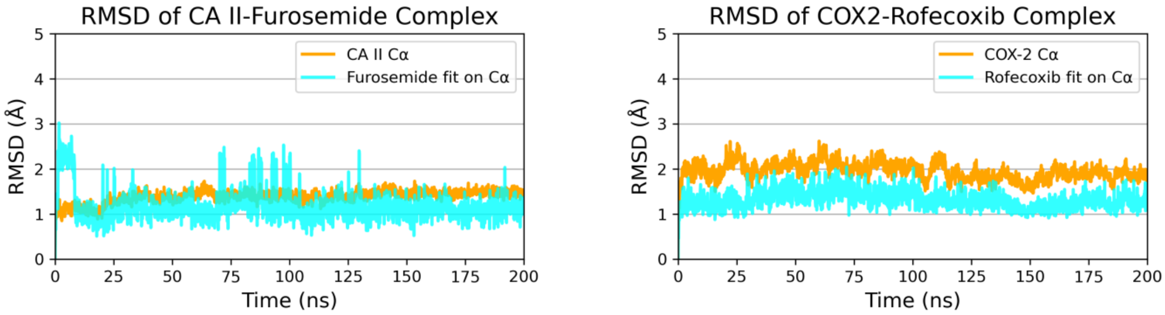

Figure S63. MD simulations of docked furosemide and rofecoxib poses

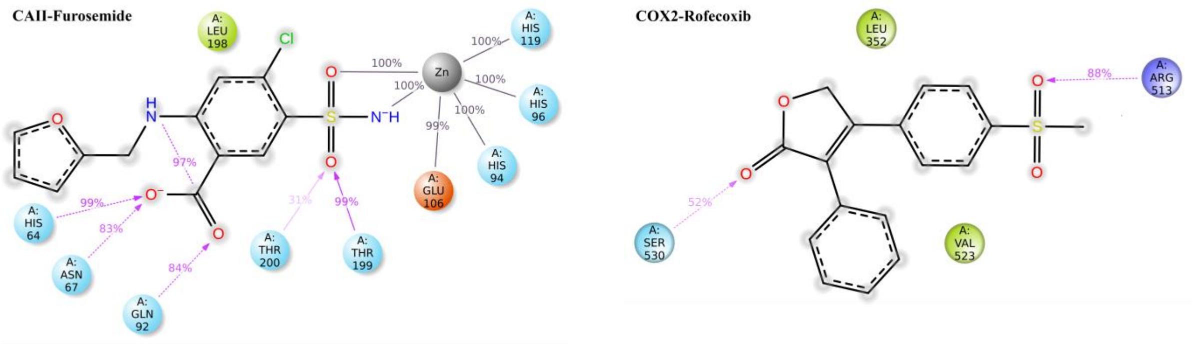

Figure S64. Interaction occupancy rates of furosemide and rofecoxib with amino acid residues

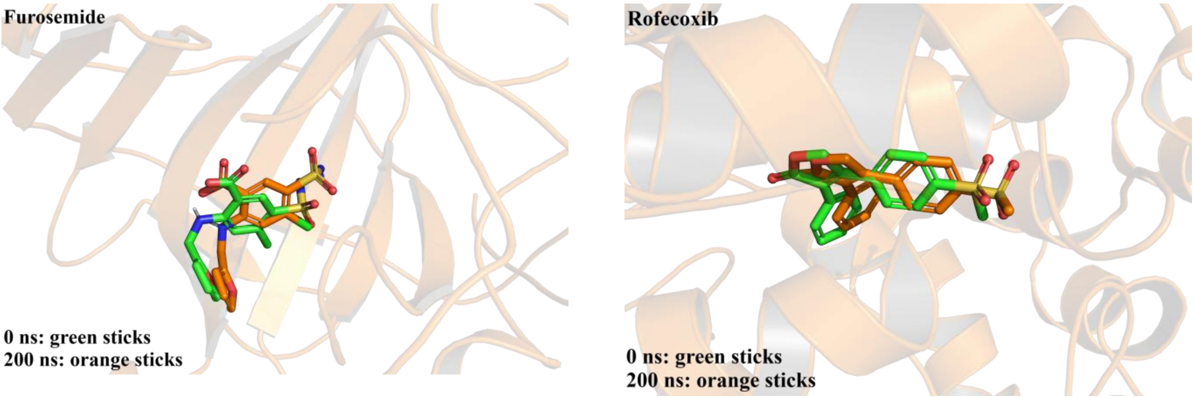

Figure S65. Snapshots of furosemide and rofecoxib at 0 ns and 200 ns of the simulation trajectories

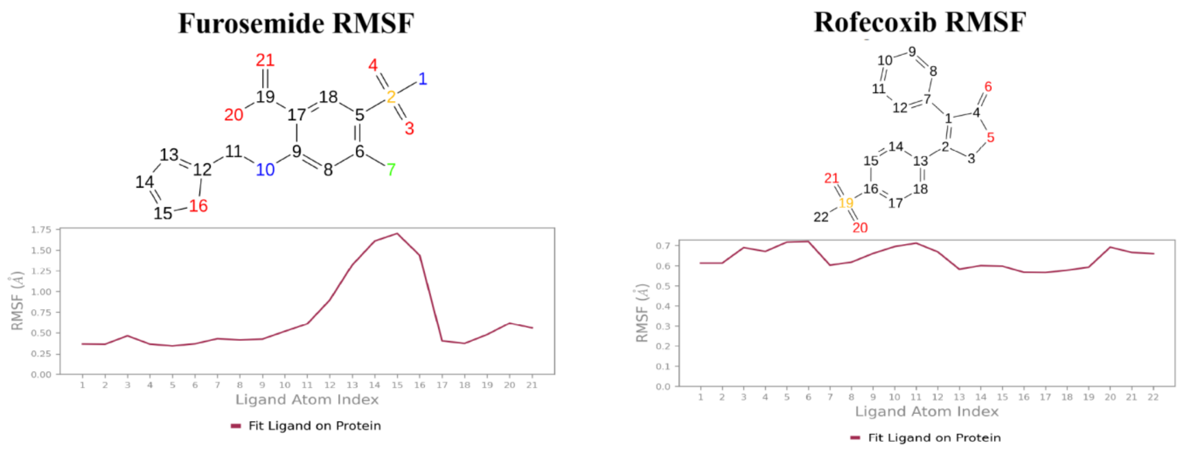

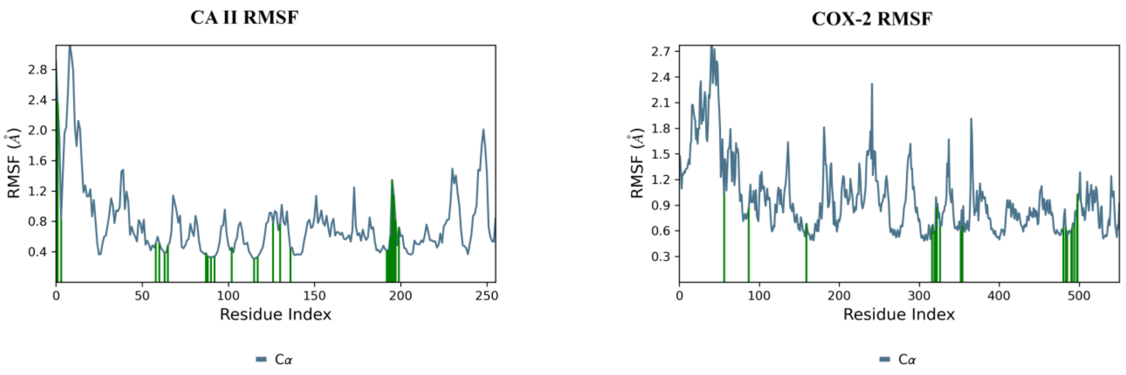

Figure S66. Root Mean Square Fluctuations (RMSF)

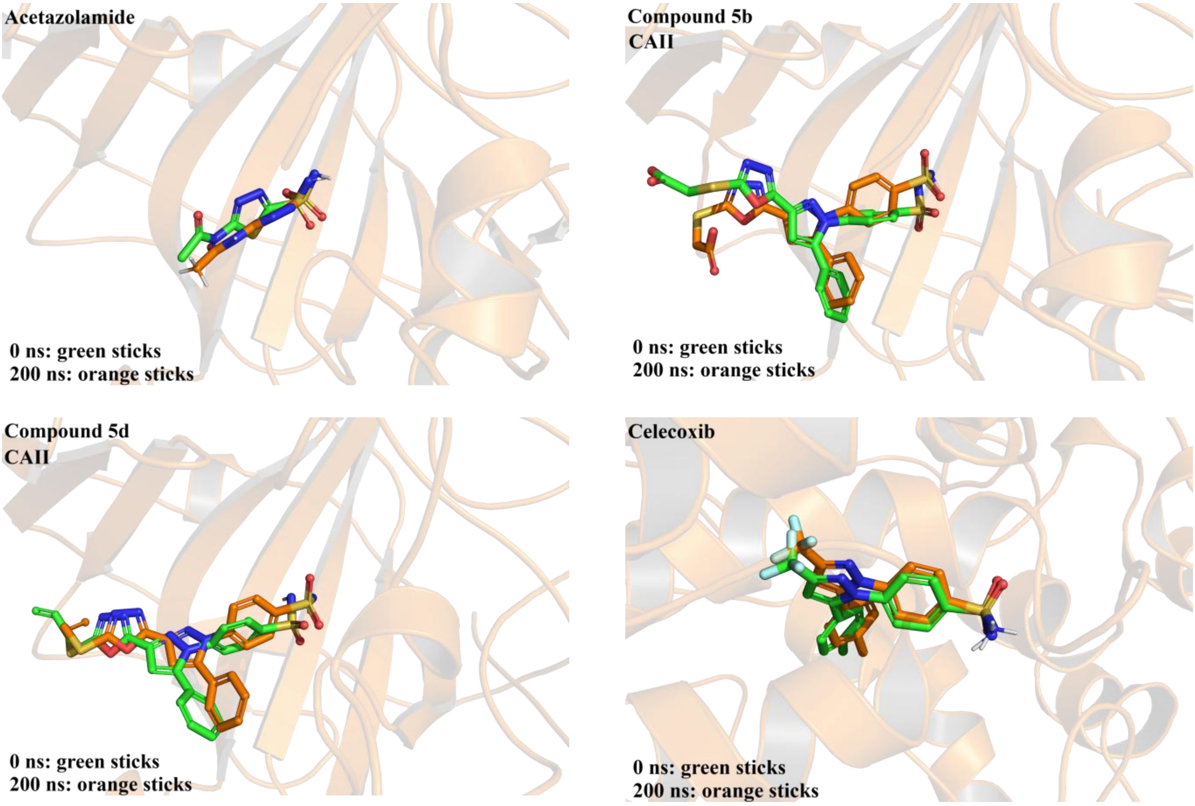

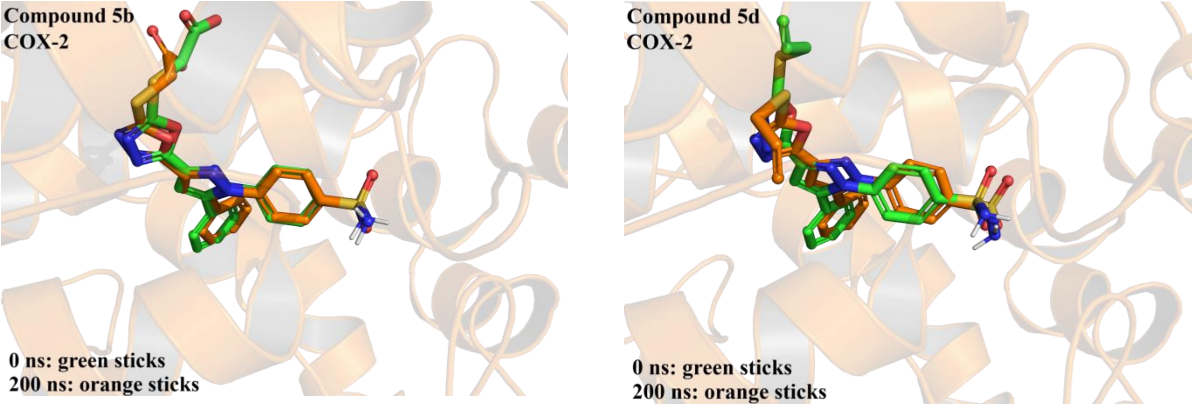

Figure S67. Snapshots of acetazolamide, celecoxib, 5b and 5d at 0 ns and 200 ns of the simulation trajectories

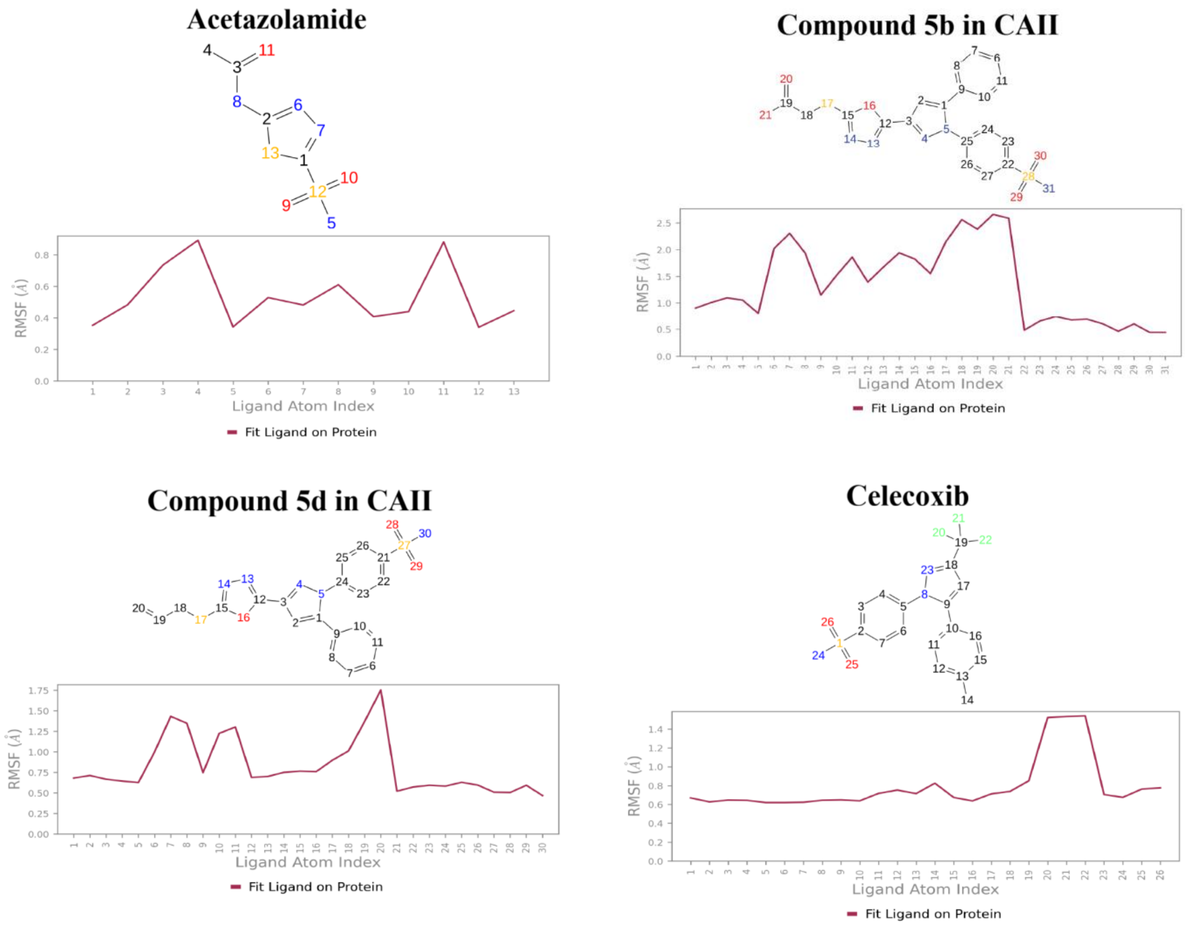

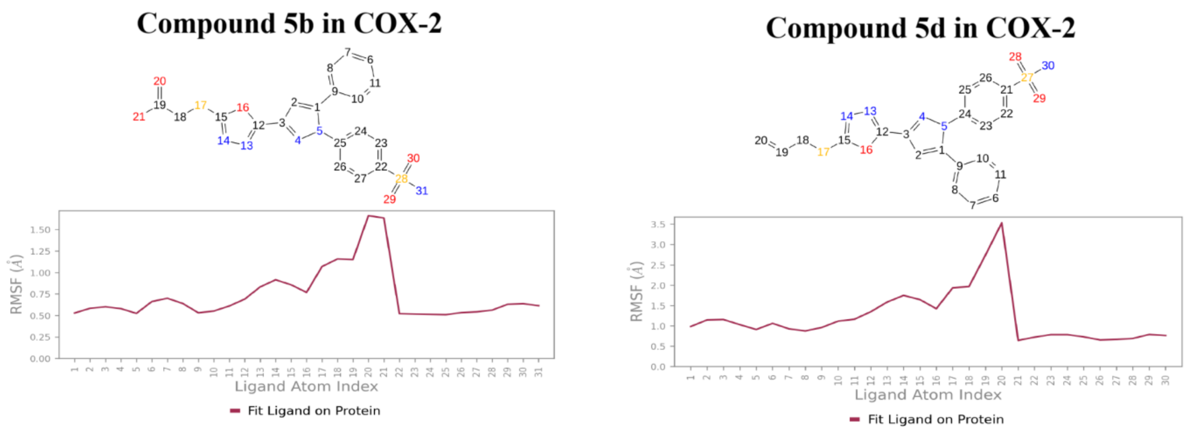

Figure S68. RMSF of acetazolamide, celecoxib, **5b** and **5d**

Table S10. *h*CAII-5b Interactions and Contact Distances within the Active Site

Interaction Type	Protein Group	Ligand Group	Distance (Å)
Hydrogen bond	Thr199 (backbone NH)	Sulfoxide oxygen	2.66
Hydrogen bond	Thr200 (backbone NH)	Sulfoxide oxygen	3.00
Hydrogen bond	Gln92 (side-chain NH2)	Pyrazole nitrogen	3.00
Hydrogen bond	Asn67 (side-chain NH2)	Oxadiazole nitrogen 1	2.57
Hydrogen bond	Asn67 (side-chain NH2)	Oxadiazole nitrogen 2	3.00
Electrostatic/coordination	Zn^{+2}	Sulfonamide NH^-	2.20
π–π stacking	Phe131 (phenyl ring)	phenyl ring	5.03
π–alkyl / hydrophobic	Leu198 (side-chain isobutyl)	phenyl ring	3.48

Table S11. *h*CAII-5d Interactions and Contact Distances within the Active Site

Interaction Type	Protein Group	Ligand Group	Distance (Å)
Hydrogen bond	Thr199 (backbone NH)	Sulfoxide oxygen	2.88
Hydrogen bond	Thr200 (backbone NH)	Sulfoxide oxygen	3.00

Hydrogen bond	Gln92 (side-chain NH2)	Pyrazole nitrogen	2.85
Hydrogen bond	Asn67 (side-chain NH2)	Oxadiazole nitrogen 1	3.00
Electrostatic/coordination	Zn^{+2}	Sulfonamide NH^{-}	2.25
π–π stacking	Phe131 (phenyl ring)	phenyl ring	5.33
π–alkyl / hydrophobic	Leu198 (side-chain isobutyl)	phenyl ring	3.94

Table S12. COX2-5b Interactions and Contact Distances within the Active Site

**Interaction Type**	**Protein Group**	**Ligand Group**	**Distance (Å)**
Hydrogen bond	Leu352 (backbone oxygen)	Sulfonamide NH2	2.95
Hydrogen bond	Gln192 (side-chain oxygen)	Sulfonamide NH2	2.90
Hydrogen bond	Phe518 (backbone NH)	Sulfoxide oxygen	3.00
Hydrogen bond	Arg513 (side-chain NH2)	Sulfoxide oxygen	2.91
π–cation	Arg513 (side-chain N+)	Pyrazole ring	5.88
π–alkyl / hydrophobic	Val523 (side-chain isopropyl)	phenyl ring	3.41

Table S13. COX2-5d Interactions and Contact Distances within the Active Site

**Interaction Type**	**Protein Group**	**Ligand Group**	**Distance (Å)**
Hydrogen bond	Leu352 (backbone oxygen)	Sulfonamide NH2	2.96
Hydrogen bond	Gln192 (side-chain oxygen)	Sulfonamide NH2	2.89
Hydrogen bond	Phe518 (backbone NH)	Sulfoxide oxygen	3.00
Hydrogen bond	Arg513 (side-chain NH2)	Sulfoxide oxygen	2.86
π–cation	Arg513 (side-chain N+)	Pyrazole ring	5.86
π–alkyl / hydrophobic	Val523 (side-chain isopropyl)	phenyl ring	3.47

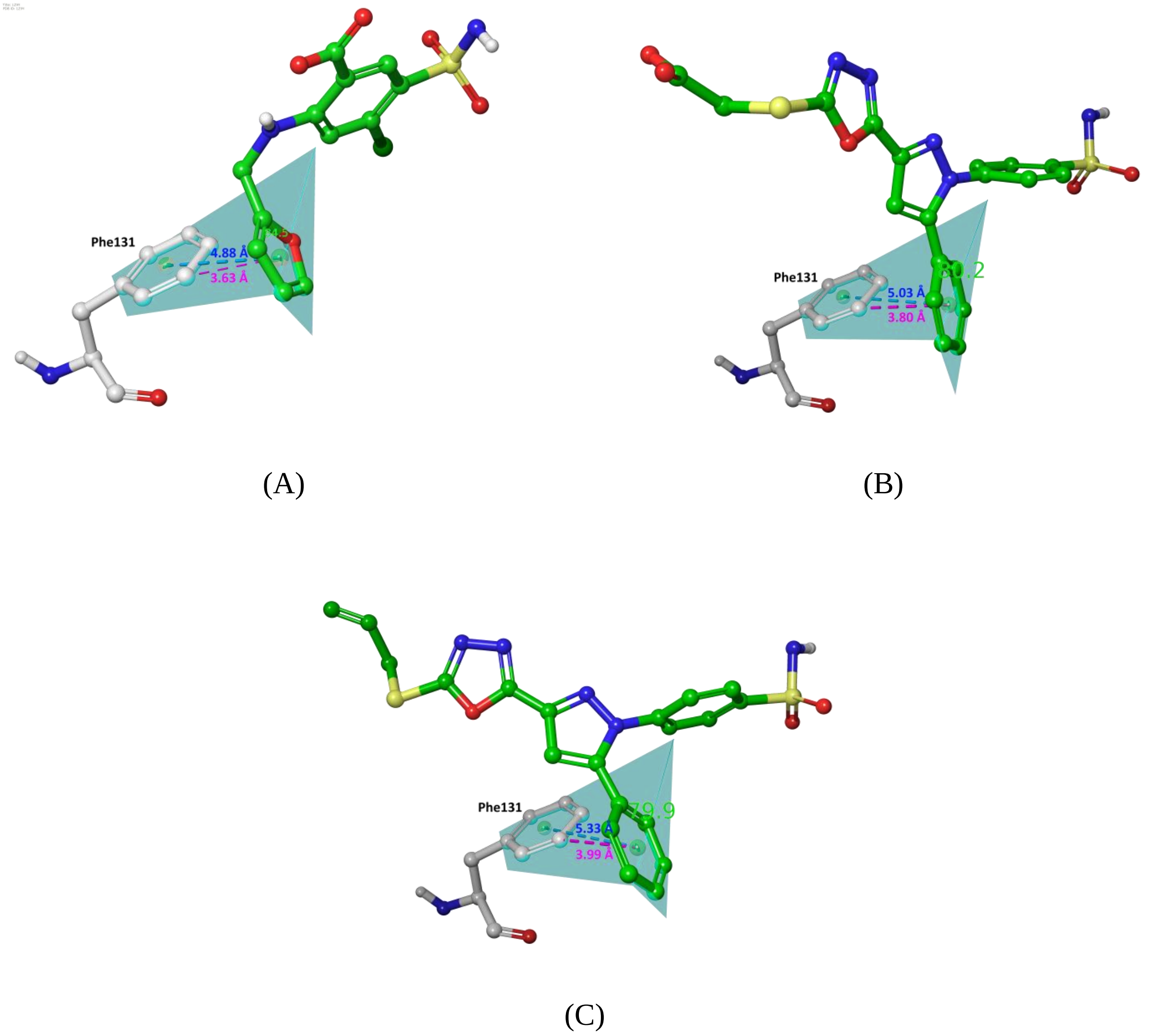

Figure S69. The spatial relationship between the phenyl ring of Phe131 in *h*CAII and the aromatic rings of (A) furosemide, (B) compound 5b and (C) compound 5d. The closet atom-centroid distance is colored pink while the centroid-centroid distance is colored blue. The angle between the planes of the rings is approximately 80°. These metrics support the formation of a T-shaped (edge to face) π- π stacking interactions.
